# Beyond apples and pears: sex-specific genetics of body fat percentage

**DOI:** 10.3389/fendo.2023.1274791

**Published:** 2023-10-05

**Authors:** Delnaz Roshandel, Tianyuan Lu, Andrew D. Paterson, Satya Dash

**Affiliations:** ^1^ Genetics and Genome Biology Program, The Hospital for Sick Children, Toronto, ON, Canada; ^2^ Department of Statistical Sciences, University of Toronto, Toronto, ON, Canada; ^3^ Divisions of Epidemiology and Biostatistics, Dalla Lana School of Public Health, University of Toronto, Toronto, ON, Canada; ^4^ Department of Medicine, University Health Network, and University of Toronto, Toronto, ON, Canada

**Keywords:** body fat percentage, obesity, genome-wide association study, testosterone, sex hormone binding globulin

## Abstract

**Introduction:**

Biological sex influences both overall adiposity and fat distribution. Further, testosterone and sex hormone binding globulin (SHBG) influence adiposity and metabolic function, with differential effects of testosterone in men and women. Here, we aimed to perform sex-stratified genome-wide association studies (GWAS) of body fat percentage (BFPAdj) (adjusting for testosterone and sex hormone binding globulin (SHBG)) to increase statistical power.

**Methods:**

GWAS were performed in white British individuals from the UK Biobank (157,937 males and 154,337 females). To avoid collider bias, loci associated with SHBG or testosterone were excluded. We investigated association of BFPAdj loci with high density cholesterol (HDL), triglyceride (TG), type 2 diabetes (T2D), coronary artery disease (CAD), and MRI-derived abdominal subcutaneous adipose tissue (ASAT), visceral adipose tissue (VAT) and gluteofemoral adipose tissue (GFAT) using publicly available data from large GWAS. We also performed 2-sample Mendelian Randomization (MR) using identified BFPAdj variants as instruments to investigate causal effect of BFPAdj on HDL, TG, T2D and CAD in males and females separately.

**Results:**

We identified 195 and 174 loci explaining 3.35% and 2.60% of the variation in BFPAdj in males and females, respectively at genome-wide significance (GWS, p<5x10^-8^). Although the direction of effect at these loci was generally concordant in males and females, only 38 loci were common to both sexes at GWS. Seven loci in males and ten loci in females have not been associated with any adiposity/cardiometabolic traits previously. BFPAdj loci generally did not associate with cardiometabolic traits; several had paradoxically beneficial cardiometabolic effects with favourable fat distribution. MR analyses did not find convincing supportive evidence that increased BFPAdj has deleterious cardiometabolic effects in either sex with highly significant heterogeneity.

**Conclusions:**

There was limited genetic overlap between BFPAdj in males and females at GWS. BFPAdj loci generally did not have adverse cardiometabolic effects which may reflect the effects of favourable fat distribution and cardiometabolic risk modulation by testosterone and SHBG.

## Introduction

Obesity is a chronic multisystem disease which affects more than 600 million adults and 100 million children (1). Cardiometabolic diseases/traits such as insulin resistance, dyslipidemia, type 2 diabetes (T2D) and coronary artery disease (CAD) are leading causes of morbidity and mortality in people with obesity (2).

Obesity/adiposity are highly heritable: more than 1000 genetic loci have been associated with adiposity and related traits (3, 4). Although obesity increases the risk of cardiometabolic disease, this can be further modulated by fat distribution (5–8). Some adiposity associated loci are paradoxically associated with improved cardiometabolic profile in part due to ‘favourable’ fat distribution (increased subcutaneous femoro-gluteal adiposity and/or reduced centripetal/visceral adiposity) (5, 6, 8–10).

**Figure 1:**
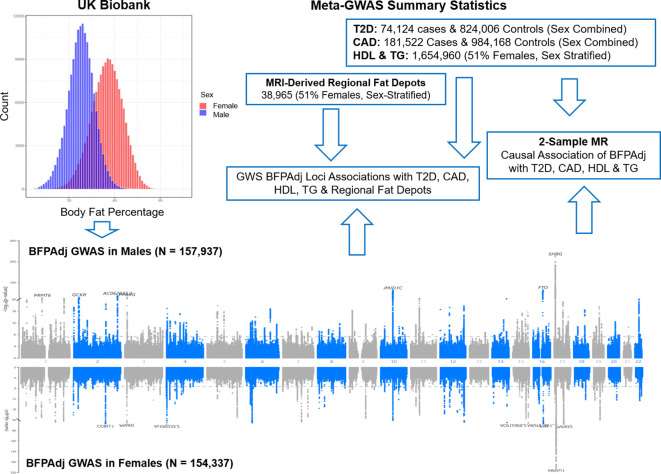
Graphical presentation of the study. Sex-stratified GWAS of body fat percentage adjusted for a number of covariates (BFPAdj) were performed in 157,937 males and 154,337 females from the UK Biobank. Independent GWS BFPAdj loci were investigated for association with T2D (21), CAD (22), HDL (23), TG (23) and MRI-derived regional fat depots (10) using publicly available summary statistics from their corresponding largest meta-GWAS. Two-sample Mendelian randomization (MR) was used to investigate the causal association of BFPAdj with T2D, CAD, HDL and TG using summary statistics from the current GWAS of BFPAdj and publicly available summary statistics from the largest meta-GWAS of T2D (21), CAD (22), HDL and TG (23).

Body mass index (BMI) is commonly used to diagnose obesity but is an imperfect measure of overall adiposity (11). A previous GWAS of body fat percentage (BFP) in the UK Biobank identified 12 loci (12). Sex and sex hormones differentially impact adiposity (13). On average females have higher BFP with ‘favourable’ fat distribution. Genetic and observational data indicate that increased testosterone has beneficial effects on adiposity/metabolic traits in men, but may be deleterious in women (13–18). Sex hormone binding globulin (SHBG) modulates bioavailable sex hormone concentration and may independently influence adiposity and cardiometabolic traits (13). Heritability of BFP was estimated around 0.27 in both males and females with high genetic correlation (>90%) between the two sexes. Similarly, heritability of SHGB was estimated around 0.17-0.19 in males and females with high genetic correlation (>90%) (19, 20). However, heritability of testosterone was estimated 0.12 and 0.07 in males and females, respectively, with very low genetic correlation ~10% (19, 20). Given these important sex differences, we undertook sex-stratified genome-wide association studies (GWAS) of BFP adjusted for total testosterone and SHBG in the UK Biobank (BFPAdj). We hypothesized this would increase statistical power to detect sex-specific BFPAdj loci at genome-wide significance (GWS). Given the differences between sexes in fat distribution and cardiometabolic risk, we further investigated the association of identified loci with fat distribution, high density lipoprotein cholesterol (HDL), triglyceride (TG), T2D and CAD and undertook Mendelian randomization (MR) to investigate cardiometabolic effects of BFPAdj (**Figure 1**).

## Methods

Ethics approval for this study was obtained in the Hospital for Sick Children (HSC #1000073707).

### Study population

#### Inclusion criteria

All white British subjects (Unique Data Identifier (UDI) 22006-0.0 = 1) from the UK Biobank with no missing data for BFP (UDI 23099-0.0), age when attended assessment centre (UDI 21003-0.0), serum albumin (UDI 30600-0.0), serum SHBG (UDI 30830-0.0) or serum testosterone (UDI 30850-0.0). Sex was defined using UDI 22001-0.0 (female = 0, male = 1).

#### Exclusion criteria

Sex chromosome aneuploidy (UDI 22019-0.0 = 1), exclusion from kinship inference process, those with ten or more third-degree relatives (UDI 22021-0.0 = 10 or -1), people on medical treatments that may interfere with sex hormones (N = 1,683 males & 1,628 females, list of medications available in **Table S1**, and females with testosterone levels >10 nmol/L (N = 28,471).

### Association of covariates with BFP

Multivariable linear regression was used for testing the association of covariates (i.e. age, age^2^, serum albumin, centered albumin^2^, serum SHBG, centered SHBG^2^, serum testosterone, centered testosterone^2^) with BFP in males and females separately using R v3.5. To test BFP mean and variance difference in males and females, t.test and var.test were used respectively in R v4.2.1 (24).

### GWAS

GWAS (Chr1-22 & X) were performed using REGENIE (v3.1.1) (25) on the research analysis platform (RAP). In step 1, only genotyped single nucleotide polymorphisms (SNPs) with minor allele count (MAC) >80 (minor allele frequency ~ 0.001) and Hardy-Weinberg Equilibrium (HWE) p >1E-15 were included in the analysis. SNPs with inter-chromosome linkage disequilibrium (LD) (25) were excluded. In step 2, all SNPs comprising centrally imputed to the Haplotype Reference Consortium (HRC) or the UK10K + 1000 Genomes phase 3 panel if the SNP was not available in HRC with MAC >80 and high imputation quality (INFO > 0.5) were included.

BFP (UDI 23099-0.0) measured by Tanita BC418MA body composition analyser was the outcome. Previous analyses indicates that BFP assessed by bioimpedance is strongly associated with dual energy X-ray absorptiometry (DEXA) based measures of adiposity (Pearson correlation coefficient = 0.92) (26). Age (UDI 21003-0.0) and its quadratic term (centered age^2^), serum albumin (UDI 30600-0.0) and its quadratic term (centered albumin^2^), serum SHBG (UDI 30830-0.0) and its quadratic term (centered SHBG^2^), serum testosterone (UDI 30850-0.0) and its quadratic term (centered testosterone^2^), and first ten genetic principal components (PCs, UDI 22009-0.1-10) were included in the model as covariates. The GWAS was performed in males and females separately.

**Figure 2:**
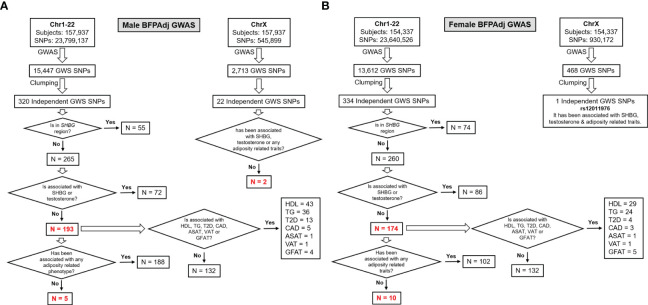
Study design and major findings. **(A)** Males, **(B)** Females. Sex-stratified GWAS of BFPAdj were performed in 157,937 males and 154,337 females from the UK Biobank. Clumping was performed to identify independent GWS SNPs. The SNPs located on the *SHBG* region and those associated with SHBG or testosterone at GWS were excluded (13). The remaining SNPs were examined for association with adiposity related phenotypes, T2D (21), CAD (22), HDL (23), TG (23) and MRI-derived regional fat depots using publicly available summary statistics from their corresponding largest meta-GWAS.

To identify independent GWAS signals, GWS SNPs (p< 5E-8) were clumped with r^2^ set at 0.1 and radius set at 500kb (less than 250kb away from an index variant) in PLINK v2 using a random sample of 5000 participants included in the GWAS as reference. These loci were excluded from further investigation if they were associated with SHBG or testosterone in their corresponding sex (p<5E-8) (13) to avoid collider bias (27) (**Figure 2**, **Table S2**).

To calculate the variance in BFPAdj explained by the identified SNPs in each sex, we performed clumping with a much stricter r^2^ (< 0.001) and used linear regression in R v4.2.1 (24) with the same covariates included in the GWAS to test the association of individual SNPs with BFPAdj. The variance explained by each SNP was calculated by subtracting the base model (only covariates in the model) R^2^ from the full model (SNP + all covariates) R^2^. Subsequently, the total variance explained was calculated as sum of the variance explained by individual SNPs.

Association of identified independent loci were investigated with T2D (21), CAD (22), sex-stratified HDL (23) and TG (23); and MRI-derived abdominal subcutaneous adipose tissue (ASAT), visceral adipose tissue (VAT) and gluteofemoral adipose tissue (GFAT) adjusted for BMI and height in their corresponding sex (10). For SNPs having paradoxical effect on BFPAdj and lipid levels, we also investigated their association with waist-hip ratio (WHR) adjusted for body mas index (BMI) (3) (**Figure 2**, **Table S2**).

To identify novel loci, we investigated if the SNPs were associated with BFP (12), BMI (3), WHR (3), or any other adiposity related phenotypes [e.g. appendicular lean mass (28) and body fat distributed to the arms, legs and trunk (29)] previously, by examining the NHGRI-EBI GWAS catalogue [https://www.ebi.ac.uk/gwas, accessed Nov 2022 (30)] and Neale’s round 2 GWAS results (http://www.nealelab.is/uk-biobank; UKBB GWAS Imputed v3 - File Manifest Release 20180731).

We also performed a separate GWAS (Chr1-22) including both sexes to investigate SNP x Sex interaction using REGENIE. Identical SNP inclusion criteria and covariates (including sex) were used in the model. The results of the test for interaction effect (i.e. ADD-INT_SNPxSex=0) were reported.

### HLA imputation

Association of 362 four-digit HLA haplotypes (UDI 22182-0.0) were tested with BFP in males and females separately using linear regression in R v4.2.1 (24) with age, age^2^, albumin, albumin^2^, SHBG, SHBG^2^, testosterone, testosterone^2^ and first ten genetic PCs as covariates in the model.

### Mendelian randomization

Two-sample MR was used to investigate the causal effect of BFPAdj (exposure) on T2D, CAD, HDL and TG (outcomes) in males and females separately using MR-Base platform (31). For exposure, we used summary statistics from our current analyses and performed the clumping with r^2^<0.001 and a 5000 random sample of UK Biobank participants as LD reference. For outcomes, we used publicly available summary statistics from the published GWAS as explained in the main text (**Table S2**) (21–23). Palindromic SNPs with intermediate allele frequencies were excluded. We used five methods for MR analysis including MR Egger, inverse variance weighted (IVW), weighted median, simple mode and weighted mode. These five methods have different assumptions regarding the validity of SNPs employed as instruments. Specifically, the IVW method assumes that all SNPs are valid instruments, which should satisfy the three core instrumental variable assumptions of MR: relevance, independence, and no horizontal pleiotropy (32, 33). In contrast, the weighted median method mandates that no less than 50% of the weight in the analysis originates from valid instruments (34). On the other hand, the mode-based methods require that most substantial subset of instruments that converge on the same causal effect should be valid instruments (35). However, we primarily focused on MR Egger results. MR Egger relaxes “no horizontal pleiotropy” assumption (the effects of the SNPs on the outcome not mediated by the exposure) allowing the net-horizontal pleiotropic effect across all SNPs to be unbalanced or directional. It returns an unbiased causal effect even if the “no horizontal pleiotropy” assumption is violated for all SNPs. Heterogeneity was tested with MR Egger and IVW methods. Horizontal pleiotropy was tested using MR Egger intercept. The Wald ratio method was used for single SNP MR and the IVW method was used for leave-one-out analysis (31). We also conducted horizontal pleiotropic outlier detection implemented in MR-PRESSO (36) to examine whether there exist instruments whose effects are not consistent with the overall causal effect estimate.

We derived the F-statistic for each MR estimate with.


$$F=\frac{N-k-1}{k}\frac{R^{2}}{1-R^{2}}$$

where *N* = the sample size of the exposure GWAS; *k* = the number of SNPs used as instruments; *R* ^2^ = the proportion of variance in the exposure explained by all SNPs used as instruments. An F-statistic >10 was considered evidence against weak instrument bias (37). Furthermore, we used Steiger filtering to examine whether each instrument has the expected direction of effect (38), which assumes that an instrument should explain more variance in the exposure than the outcome. We repeated MR analyses after removing instruments that did not withstand Steiger filtering, using MR Egger, inverse variance weighted (IVW), weighted median, simple mode, and weighted mode methods. Lastly, as we used exposure GWAS and outcome GWAS that can have partial sample overlap, we additionally implemented latent causal variable (LCV) model to distinguish genetic or residual correlation from causation (39). Using GWAS summary statistics of HapMap3 variants, LCV infers a genetic causality proportion (gcp) between an exposure and an outcome, where a gcp >0.6 indicates potential causal effects of the exposure on the outcome (39).

## Results

### Males

#### Autosomal GWAS

157,937 males were included in the analysis (**Table S3**). One male was excluded from analysis as his whole-body fat plus fat-free mass was greater than his weight. BFP was normally distributed with mean (SD) of 25.3 (5.8) % (**Figure S1A**). In the multivariable analysis, age was associated with higher BFP whereas albumin, SHBG and testosterone were all associated with lower BFP. Age, albumin, SHBG, testosterone, and their quadratic terms together explained 13% of variation in BFP. Testosterone and its quadratic term explained 8% of variation in BFP (**Tables S4**, **S5**).

**Figure 3:**
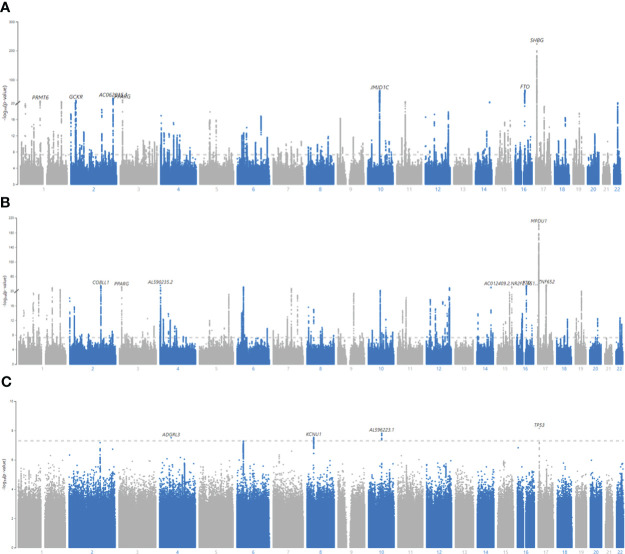
Manhattan plots for BFPAdj GWAS in males, females and SNP x Sex interaction. **(A)** Males, **(B)** Females, **(C)** All, SNP x Sex interaction. GWAS of BFPAdj was performed in males (N = 157,937) and females (N = 154,337) separately as well as in both males and females (N = 312,274). Age, centered age^2^, serum albumin, centered albumin^2^, serum SHBG, centered SHBG^2^, serum testosterone, centered testosterone^2^, and first ten genetic PCs were included in the model as covariates. SNP x Sex interaction was also included in the last GWAS including both sexes. The X axis shows the SNP location in the genome and the Y axis shows -log_10_ (p-value) regarding SNP association with BFPAdj in males **(A)**, females **(B)**, and SNP x Sex interaction in both sexes **(C)**. The plots were made using MyLocusZoom (https://my.locuszoom.org/).

23,799,137 SNPs on Chr1-22 were included in the GWAS (GC lambda = 1.20) (**Figure 3A**). 15,447 SNPs were associated with BFPAdj at GWS level (**Supplementary Males.xlsx File, Sheet A**). There were 320 independent GWS SNPs including 55 SNPs in *SHBG* (Chr7:7.4Mb; **Supplementary Males.xlsx File, Sheet B**). Less than half (119 out of 320) of these independent GWS SNPs were associated with BFPAdj in females, all with the same direction of effect as males (**Supplementary Males.xlsx File, Sheet C**). Five SNPs in *SHBG* region were missing from Ruth et al. analysis (13) of SHBG/Testosterone; but the rest (N = 50) were all associated with SHBG and/or testosterone. Of 265 SNPs in non-*SHBG* loci, 193 were not associated with SHBG or testosterone including an indel (Chr4:99262829, TG>T) missing in Ruth et al. analysis (13) (**Supplementary Males.xlsx File, Sheet D**; **Figures S2**, **S3**). Of these 193 loci, only 38 were associated with BFPAdj in females at GWS. The directions of effect were generally consistent in both sexes with effect sizes being smaller in females (**Figures S4**, **S5**). After, further clumping of these 193 SNPs with r^2^< 0.001, 161 SNP were left, and they explained 3.35% of the variation in BFPAdj in males (**Supplementary Males.xlsx File, Sheet J**).

#### Novel autosomal BFPAdj loci

Of these 193 SNPs, 188 were not associated with BFP in prior published GWAS (12) and 94 were not identified by Neale’s round 2 GWAS results of BFP in males (**Supplementary Males.xlsx File, Sheet I**; **Figure S6**). Five of these 193 autosomal SNPs, have not been associated with any adiposity related phenotypes (**Table 1**), fat depots (**Table S6**), HDL, TG, T2D or CAD (**Supplementary Males.xlsx File, Sheet E**) previously. Of these 5 SNPs, only rs768147154 was nominally associated with albumin (p = 0.004), SHBG (p = 0.005) and testosterone (p = 0.004). The other four SNPs were not associated with albumin, SHBG or testosterone (p >0.05) (**Table S7**).

**Table 1:** Newly identified loci for BFPAdj in males or females not previously associated with adiposity related phenotypes.

						Males					Females					SNPxSex
CHR	BP (HG19)	A0	A1	SNP	Nearest Gene/s	A1FREQ	INFO	BETA	SE	LOG10P	A1FREQ	INFO	BETA	SE	LOG10P	LOG10P
1	106,802,195	G	A	rs11184828	*PRMT6*	0.29	1.00	0.07	0.02	3.09	0.28	1.00	0.14	0.02	**8.65**	1.58
1	150,055,361	T	C	rs116819476	*VPS45*	0.97	0.91	-0.17	0.05	2.79	0.97	0.91	-0.33	0.06	**7.39**	1.56
1	222,061,973	C	T	rs11577023	*DUSP10*	0.69	0.99	0.06	0.02	2.66	0.69	0.99	0.12	0.02	**7.32**	1.57
2	54,881,621	C	T	rs2941584	*SPTBN1*	0.32	1.00	0.04	0.02	1.26	0.32	0.99	0.12	0.02	**7.68**	2.83
2	101,414,496	T	C	rs2309885	*NPAS2*	0.53	0.98	-0.10	0.02	**7.44**	0.53	0.98	-0.03	0.02	0.65	2.02
3	30,071,380	G	A	rs7426945	*RBMS3*	0.45	1.00	-0.05	0.02	2.35	0.45	1.00	-0.12	0.02	**8.06**	2.11
4	2,405,062	G	C	rs35802140	*ZFYVE28*	0.88	0.99	-0.07	0.03	1.79	0.89	0.99	-0.19	0.03	**8.73**	2.45
10	126,305,434	C	T	rs4962671	*LHPP, FAM53B*	0.53	0.99	0.10	0.02	**7.91**	0.53	0.99	0.03	0.02	0.98	1.55
10	131,430,686	A	G	rs524804	*MGMT*	0.58	1.00	0.07	0.02	3.46	0.58	1.00	0.13	0.02	**8.75**	1.21
11	66,820,856	A	C	rs117773425	*RHOD, SYT12*	0.98	0.94	-0.39	0.07	**7.68**	0.98	0.94	0.00	0.08	0.01	3.18
13	76,086,882	CTTTTTTT	C	rs531470369	*COMMD6*	0.35	0.96	-0.03	0.02	1.11	0.35	0.96	-0.12	0.02	**7.39**	2.59
16	25,247,974	T	C	rs151118254	*ZKSCAN2*	0.99	0.89	-0.17	0.12	0.85	0.99	0.89	-0.73	0.13	**7.42**	2.63
19	47,282,245	T	C	rs56385874	*SLC1A5*	0.82	1.00	-0.05	0.02	1.30	0.82	1.00	-0.17	0.03	**9.32**	2.55
20	38,490,795	T	TAGAG	rs768147154	*DHX35*	0.74	0.99	-0.12	0.02	**8.10**	0.74	0.99	-0.06	0.02	1.77	1.39
20	55,823,762	A	G	rs6127980	*BMP7*	0.88	1.00	-0.15	0.03	**7.31**	0.88	1.00	-0.03	0.03	0.48	2.13
23	43,017,461	C	T	rs5950969	*PINCR*	0.86	0.98	0.10	0.02	**7.46**	0.86	0.99	-0.02	0.03	0.25	–
23	83,562,659	G	A	rs73505165	*HDX*	0.50	0.99	0.07	0.01	**7.66**	0.50	0.99	-0.001	0.02	0.01	–

A0, Non-effect allele; A1, effect allele; A1FREQ, Frequency of A1 allele.

-log10 (p-values) >7.3 (p < 5E-8) are shown in bold.

#### Association of autosomal BFPAdj GWAS loci with cardiometabolic phenotypes

**Figure 4:**
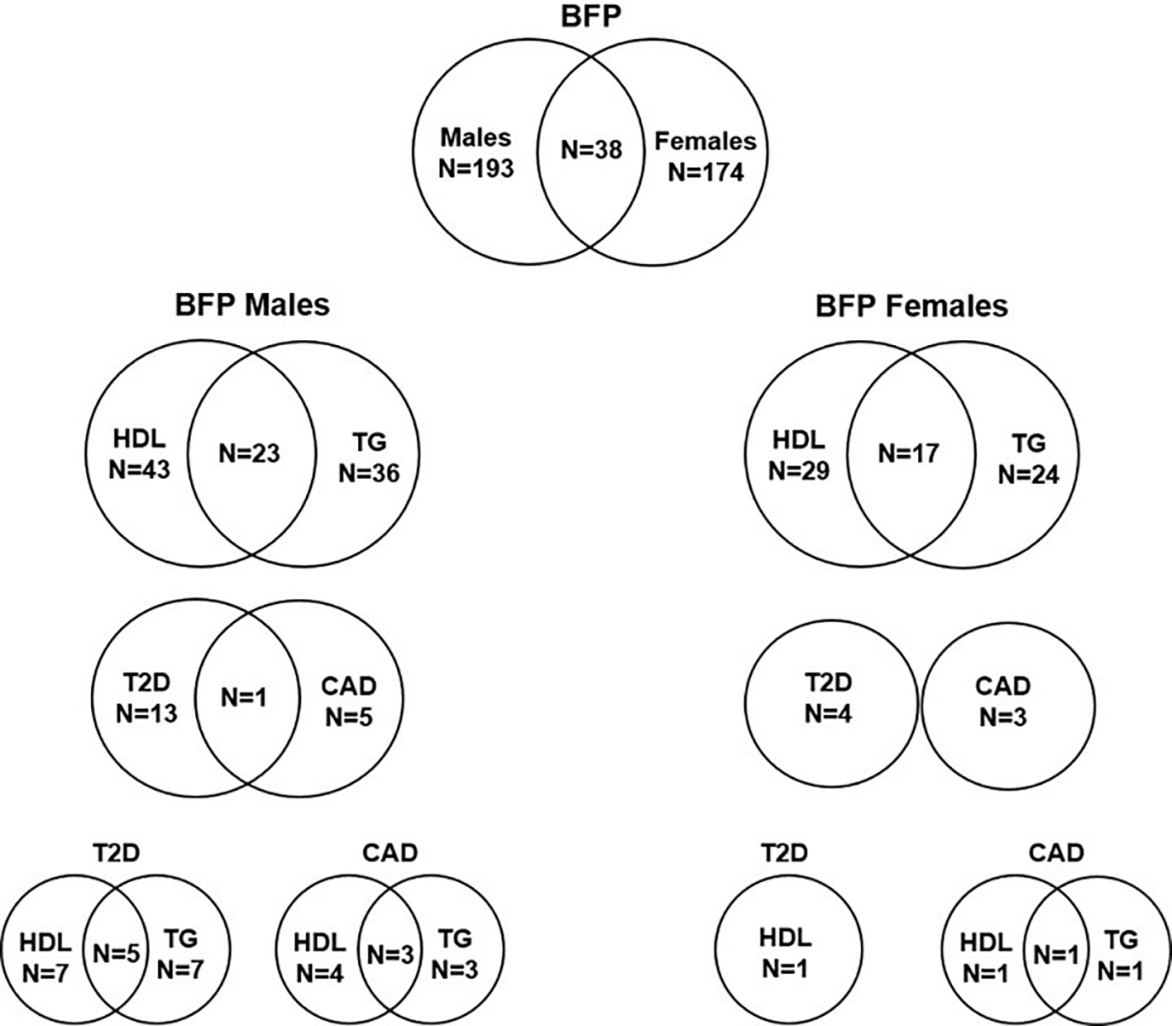
Number of independent SNPs associated with BFPAdj in males and females, and of them the number of SNPs associated with HDL, TG, T2D and CAD. The plot shows the number of independent GWS SNPs associated with BFPAdj in males or females, and those associated with BFPAdj in both sexes. It also shows the number of BFPAdj GWAS SNPs in each sex associated with T2D (21), CAD (22), HDL (23) or TG (23) as well as those associated with multiple phenotypes. The numbers in each circle shows the total number of SNPs associated with the corresponding trait and the numbers in the common area shows the number of SNPs associated with both phenotypes.

Of 193 autosomal SNPs associated with BFPAdj in males, the majority (N = 132, 68%) were not associated with cardiometabolic phenotypes. Sixty-one were associated with lipid levels, T2D, CAD or fat depots with some associated with multiple traits: HDL: 43, TG: 36, T2D: 13, CAD: 5, GFAT: 4, VAT: 1, and ASAT: 1 (**Table 2**, **Figure 4**, **Supplementary Males.xlsx File, Sheet E**).

**Table 2:** BFPAdj GWAS loci having paradoxical effect on HDL and TG.

A. Males																								
							Sex-Stratified														Sex-Combined			
							BFPAdj		WHR Adj BMI		HDL		TG		ASAT		VAT		GFAT		T2D		CAD	
SNP	CHR	BP (HG19)	Gene	A0	A1	A1FREQ	β	LOG10P	β	LOG10P	β	LOG10P	β	LOG10P	β	LOG10P	β	LOG10P	β	LOG10P	β	LOG10P	β	LOG10P
rs78058190	2	219,699,999	PRKAG3	A	G	0.95	0.26	7.51	0.008	0.51	0.09	73.09	-0.07	57.05	0.13	5.39	-0.07	1.96	0.15	7.32	-0.08	6.35	-0.07	6.85
rs112695539	2	227,004,358	NYAP2	T	G	0.96	-0.27	8.48	0.000	0.002	-0.03	13.82	0.03	12.43	-0.09	3.43	-0.05	1.24	-0.09	3.64	0.07	4.96	-0.004	0.11
rs150535373	3	12,410,328	PPARG	A	G	0.99	-0.53	11.21	-0.023	1.10	-0.05	10.48	0.05	12.47	-0.17	4.18	-0.01	0.00	-0.13	2.72	0.13	6.22	0.04	1.24
rs62271373	3	150,066,540	TSC22D2	A	T	0.94	0.24	9.03	-0.002	0.09	0.04	22.05	-0.03	15.38	0.05	1.54	0.01	0.27	0.1	4.96	-0.09	9.00	-0.06	5.96
rs71602277	4	157,714,979	PDGFC	TA	T	0.69	-0.12	8.99	NA	NA	-0.02	17.60	0.02	20.21	-0.01	0.47	0.01	0.57	-0.06	7.72	NA	NA	0.02	1.35
rs998584	6	43,757,896	VEGFA	A	C	0.52	0.12	9.84	-0.027	24.88	0.03	92.36	-0.04	111.77	0.03	1.85	-0.06	9.01	0.07	11.92	-0.04	7.64	-0.04	11.54
rs9641894	7	130,465,054	KLF14	G	T	0.58	0.11	8.66	0.008	2.73	0.02	27.41	-0.01	13.15	0.004	0.17	0.02	1.19	0.003	0.06	-0.05	13.41	-0.02	3.42
rs4741016	9	1,036,132	DMRT2	C	T	0.77	0.12	7.43	0.005	0.80	0.01	11.96	-0.01	8.51	0.02	0.66	-0.003	0.14	0.01	0.27	-0.02	2.89	-0.004	0.27
rs7133378*	12	124,409,502	DNAH10	A	G	0.68	-0.17	17.79	0.016	8.42	-0.03	44.92	0.02	20.64	-0.03	1.70	0.02	0.92	-0.05	5.33	0.03	5.47	0.03	8.40
rs1716407*	12	124,515,218	ZNF664	A	G	0.41	0.14	12.98	-0.015	7.55	0.02	23.10	-0.02	17.96	0.02	1.08	-0.02	0.89	0.05	7.00	-0.03	5.10	-0.02	6.00
rs12454712	18	60,845,884	BCL2	C	T	0.62	-0.11	7.95	-0.001	0.14	-0.01	15.80	0.01	14.42	0.01	0.68	-0.01	0.62	-0.02	1.08	0.05	11.62	0.02	3.30
rs56361048^†^	19	33,885,318	PEPD	C	T	0.86	-0.16	9.01	-0.009	1.52	-0.02	19.52	0.02	17.66	0.02	0.74	-0.05	3.21	-0.02	0.70	0.05	6.89	0.02	1.70
rs7258937^†^	19	33,938,800	PEPD	T	C	0.49	-0.16	17.48	-0.002	0.24	-0.02	24.39	0.01	12.68	0.01	0.39	-0.01	0.38	-0.04	3.85	0.03	5.32	0.02	3.62
rs190712692	19	45,425,178	APOC1	A	G	0.95	-0.24	7.93	NA	NA	-0.07	71.47	-0.15	>112	-0.05	1.60	0.02	0.19	-0.003	0.03	NA	NA	0.15	26.57
rs4821764	22	38,599,364	MAFF	A	G	0.42	0.17	20.20	0.007	1.71	0.02	33.89	-0.02	44.08	0.02	1.18	0.004	0.15	0.05	5.32	-0.03	3.96	0.001	0.07
B. Females																								
							Sex-Stratified														Sex-Combined			
							BFPAdj		WHR Adj BMI		HDL		TG		ASAT		VAT		GFAT		T2D		CAD	
SNP	CHR	BP (HG19)	Gene	A0	A1	A1FREQ	β	LOG10P	β	LOG10P	β	LOG10P	β	LOG10P	β	LOG10P	β	LOG10P	β	LOG10P	β	LOG10P	β	LOG10P
rs13303359	1	203,518,873	OPTC	C	A	0.47	-0.11	7.51	0.022	15.35	-0.01	10.48	0.02	13.46	-0.07	9.92	0.03	2.64	-0.02	1.31	0.03	5.00	0.01	1.40
rs386652275	2	165,533,198	COBLL1	TC	T	0.97	-0.40	8.08	NA	NA	-0.05	12.40	0.06	14.68	-0.001	0.01	0.04	0.68	-0.19	7.49	NA	NA	-0.01	0.30
rs10102352	8	9,206,069	TNKS	G	A	0.4	-0.17	15.60	0.012	4.75	-0.04	76.52	0.02	14.83	0.01	0.39	0.03	2.41	-0.01	0.57	0.0003	0.01	-0.01	1.37
rs10086016	8	36,847,709	KCNU1	C	T	0.84	0.22	14.99	0.004	0.52	0.02	12.20	-0.02	10.30	0.06	4.80	-0.01	0.21	-0.01	0.44	NA	NA	-0.01	1.43
rs940390	10	95,322,044	FFAR4	G	A	0.86	0.17	7.93	-0.023	11.66	0.02	12.68	-0.02	8.62	0.01	0.36	-0.02	0.55	0.05	4.41	0.01	0.39	0.004	0.23
rs150090666	11	14,865,399	PDE3B	T	C	0.999	-2.02	9.05	0.363	14.34	-0.46	31.85	0.42	26.78	NA	NA	NA	NA	NA	NA	NA	NA	NA	NA
rs7134375	12	20,473,758	PDE3A	A	C	0.57	-0.13	9.23	0.010	4.83	-0.02	31.64	0.02	22.98	-0.03	2.21	0.01	0.31	-0.03	3.10	0.01	1.41	0.01	0.62
rs11057405	12	122,781,897	CLIP1	A	G	0.89	0.30	18.42	-0.043	28.30	0.03	22.39	-0.02	12.26	-0.02	0.57	-0.07	5.16	0.09	7.80	-0.03	2.30	-0.01	0.35
rs146459385	12	123,318,455	HIP1R	A	G	0.95	0.30	9.94	-0.064	24.45	0.06	30.17	-0.04	15.48	-0.05	1.36	-0.06	2.21	0.09	4.15	-0.006	0.14	0.004	0.11
rs147935170	12	123,882,462	KMT5A	T	C	0.95	0.30	9.17	-0.063	22.04	0.05	25.52	-0.04	12.34	-0.04	1.15	-0.03	0.82	0.06	1.92	-0.01	0.41	0.004	0.10
rs74841570^¶^	12	124,407,903	DNAH10	C	A	0.95	-0.29	9.29	0.067	26.51	-0.04	21.28	0.04	15.88	0.01	0.05	0.11	5.15	-0.13	7.92	0.021	0.82	0.04	3.01
rs11057402^¶^	12	124,430,767	CCDC92	A	T	0.88	0.24	12.81	-0.051	38.95	0.04	30.14	-0.03	17.81	-0.02	0.51	-0.04	2.01	0.09	8.07	-0.04	4.60	-0.001	0.04
rs7412	19	45,412,079	APOE	T	C	0.92	-0.21	7.45	0.015	2.56	-0.1	162.03	-0.09	127.02	-0.03	1.07	-0.01	0.51	-0.04	1.46	0.002	0.07	0.15	54.00
rs4820325	22	38,599,978	MAFF	A	G	0.42	0.15	12.67	-0.015	7.40	0.02	15.18	-0.02	22.14	-0.01	0.48	-0.01	0.60	0.04	4.89	-0.03	3.96	0.001	0.06

A1, Effect Allele; A0. Non-effect allele; A1FREQ, Frequency of A1 allele; LOG10P, -log_10_ (p-value).

*These SNPs remained GWS when both were included in the model (**Table S13**).

^†^Only rs7258937 remained GWS when both SNPs were included in the model (**Table S13**).

^¶^Only rs11057402 remained GWS when both SNPs were included in the model (**Table S14**).

#### Autosomal BFPAdj GWAS loci with paradoxical effects on lipids, T2D and CAD

**Figure 5:**
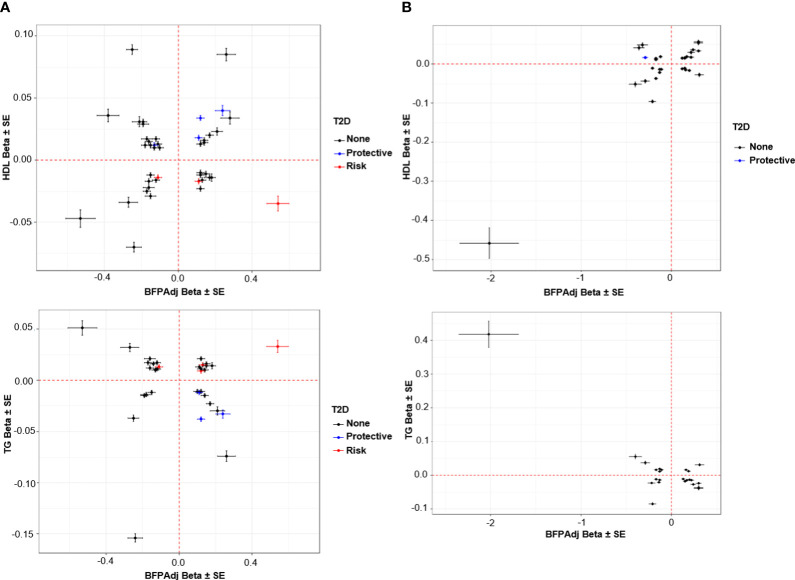
Association of SNPs with HDL and TG vs. BFPAdj. **(A)** Males, **(B)** Female. The plots show the effect (β ± SE) of BFPAdj GWAS SNPs on BFPAdj vs. HDL (top) and TG (bottom) (23). Only SNPs associated with both BFPAdj and HDL or TG at GWS are included in the plots. The SNPs with risk and protective effect on T2D (both sexes combined) at GWS level are shown in red and blue, respectively (21). The rest of SNPs are shown in black.

Lipids: Twenty SNPs had paradoxical associations with BFPAdj and HDL (i.e. the direction of effect on BFPAdj and HDL was the same); and 21 had paradoxical associations with BFPAdj and TG (i.e. the direction of effect on BFPAdj was the opposite of effect direction on TG) with 14 having paradoxical associations with both HDL and TG. Of these 14 SNPs, three were associated with WHR adjusted for BMI all with opposite direction of effect on BFPAdj and WHR including rs998584 (Chr6:43,757,896; A>C; *VEGFA*), rs7133378 (Chr12: 124,409,502; A>G; *DNAH10*) and rs1716407 (Chr12:124,515,218; A>G; *ZNF664*). rs998584 was associated with multiple cardiometabolic phenotypes: the BFPAdj increasing allele associated with increased GFAT, reduced VAT and reduced risk of T2D and CAD underscoring the role of fat distribution in cardiometabolic disease in males. rs78058190 (Chr2:219,699,999; A>G; *PRKAG3*) and rs71602277 (Chr4:157,714,979; TA>T; *PDGFC*) were also associated with fat depots with the BFPAdj increasing allele associating with increased GFAT (**Table 2**, **Figure 5A**).

Lipids and T2D: The BFPAdj increasing alleles of rs62271373 (Chr3:150,066,540; A>T; *TSC22D2*), rs9641894 (Chr7:130,465,054; G>T; *KLF14*), and rs12454712 (Chr18:60,845,884; C>T; *BCL2*) were associated with reduced T2D risk, higher HDL and lower TG (**Table 2**, **Figure 5A**).

Lipids and CAD: The BFPAdj decreasing allele of rs7133378 (Chr12:124,409,502; A>G; *DNAH10*) was associated with lower HDL and higher TG, and increased risk of CAD (**Table 2**, **Figure 5A**).

#### Chr X

545,899 SNPs on Chr X were tested for association with BFPAdj. There were 2,713 GWS SNPs including 22 independent signals (**Figure S7A**; **Supplementary Males.xlsx File, Sheets F, G**). Of these, two SNPs (rs5950969 and rs73505165) have not been associated with SHBG, testosterone or any adiposity related phenotypes previously (**Tables 1**, **S7**).

#### HLA imputation

None of the HLA haplotypes reached GWS threshold (**Supplementary Males.xlsx File, Sheet H**).

#### MR

The MR Egger method did not show any significant causal effect for BFPAdj on T2D (p = 0.58), CAD (p = 0.95), HDL (p = 0.63) or TG (p = 0.58). Some but not all the other methods showed some significant associations but overall we did not find any reliable evidence that increased BFPAdj increases risk of T2D, CAD or TG levels, or decreases HDL; and there was highly significant evidence for heterogeneity (p< 2E-110). There was no evidence for directional pleiotropy (p >0.05) (**Supplementary Males.xlsx File, Sheets K, L**).

Single SNP MR results demonstrated three groups of SNPs suggesting positive, negative and no significant causal effect of BFPAdj on the four outcomes consistent with high levels of heterogeneity.

Leaving out rs35198068 within *TCF7L2* a known locus for T2D with opposite effects on BFPAdj and T2D led to significance of MR analysis. None of the SNPs made significant difference in MR analysis results of CAD, HDL or TG in the leave-one-out analysis (**Supplementary Males MR Plots**). No SNP was deemed an outlier instrument by the MR-PRESSO horizontal pleiotropic outlier detection. Steiger filtering indicated that one instrument (rs35198068) did not show the expected direction of effect with T2D as the outcome. However, after removing this instrument, all MR estimates were similar, and the MR Egger method did not support causal effect on T2D (**Supplementary Males.xlsx File, Sheet K**).

Based on the LCV model, while we found positive genetic correlations between BFPAdj and T2D and CAD, and a negative genetic correlation between BFPAdj and HDL, causal effect of BFPAdj on these traits was not supported (all estimated gcp<0.6; **Table S8**).

### Females

#### Autosomal GWAS

154,337 females were included in the analysis (**Table S3**). BFP was normally distributed with mean (SD) of 36.7 (6.9) % (**Figure S1B**). Both mean and variance of BFP were significantly higher in females compared to males (p< 2.2E-16). In the multivariable analysis, age and testosterone were associated with higher BFP in females whereas albumin and SHBG were associated with lower BFP. Age, albumin, SHBG, testosterone, and their quadratic terms together explained 23% of variation in BFP. SHBG and its quadratic term explained 18% of variation BFP (**Tables S4**, **S5**).

23,640,526 SNPs on Chr1-22 were included in the GWAS (GC lambda = 1.19) (**Figure 3B**). 13,612 SNPs were associated with BFPAdj at GWS level (**Supplementary Females.xlsx File, Sheet A**). There were 334 independent GWS SNPs including 74 SNPs in *SHBG* (**Supplementary Females.xlsx File, Sheet B**). One-hundred and nineteen SNPs were common with males (**Supplementary Females.xlsx File, Sheet C**). Six SNPs in *SHBG* region were missing in Ruth et al. analysis (13); but the rest were all associated with SHBG and/or testosterone except for an indel (Chr17:7200613; CAA>C) which was not associated with SHBG (p = 2.00E-5) or testosterone (p = 0.47). Of 260 SNPs in non-*SHBG* loci, 174 were not associated with SHBG or testosterone (**Supplementary Females.xlsx File, Sheet D**; **Figures S2**, **S3**). Of these 174 loci, only 38 were associated with BFPAdj in males at GWS. The directions of effect were consistent in both sexes with effect sizes being generally smaller in males (**Figures S4**, **S5**). After, further clumping of these 174 SNPs with r^2^< 0.001, 140 SNP were left, and they explained 2.60% of the variation in BFPAdj in females (**Supplementary Females.xlsx File, Sheet J**).

#### Novel autosomal BFPAdj loci

Of these 174 SNPs, 169 were not associated with BFP in prior published GWAS (12)and 104 were not identified by Neale’s round 2 GWAS results of BFP in females (**Supplementary Females.xlsx File, Sheet I**; **Figure S6**). Ten of these SNPs have not been associated with any adiposity related phenotypes (**Table 1**), fat depots (**Table S6**), HDL, TG, T2D or CAD previously (**Supplementary Females.xlsx File, Sheet E**). Of these 10 SNPs, rs11577023 (p = 0.026) and rs56385874 (p = 0.045) were nominally associated with albumin; rs11184828 was nominally associated with SHBG (p = 0.025); and rs531470369 was nominally associated with testosterone (p = 0.044). No other association was observed between these SNPs and albumin, SHBG or testosterone (p >0.05) (**Table S7**).

#### Association of autosomal BFPAdj GWAS loci with cardiometabolic phenotypes

Of 174 autosomal SNPs associated with BFPAdj in females, the majority (132, 75%) did not associate with cardiometabolic phenotypes. Forty-two SNPs were associated with lipid levels, T2D, CAD or fat depots with some associated with multiple traits: HDL: 29, TG: 24, T2D: 4, CAD: 3, GFAT: 5, VAT: 1, and ASAT: 1 (**Table 2**; **Figure 4**; **Supplementary Females.xlsx File, Sheet E**).

#### Autosomal BFPAdj GWAS loci with paradoxical effects on cardiometabolic phenotypes

Eighteen SNPs had paradoxical associations with BFPAdj and HDL (i.e. the direction of effect on BFPAdj and HDL was the same); and 16 had paradoxical associations with BFPAdj and TG (i.e. the direction of effect on BFPAdj was the opposite of effect direction on TG) with 13 having paradoxical associations with both HDL and TG. This included a rare rs150090666 (Chr11:14,865,399, Freq = 0.001) stop-gain (**C**GA>**T**GA, Arg861>*) variant within *PDE3B* (NP_001350499.1) with a large effect on BFPAdj (β (SE) = 2.02 (0.33), -log_10_(p) = 9.05) as well as HDL (β (SE) = 0. 458 (0.039), p = 1.40E-32) and TG (β (SE) = -0.418 (0.039), p = 1.66E-27) (**Figure 5B**). Rare coding variants in *PDE3B* have been associated with BMI adjusted WHR in sex-combined analyses previously (10, 40). Of the 13 SNPs with paradoxical effect on HDL and TG, nine were associated with WHR adjusted for BMI all with opposite direction of effect on BFPAdj and WHR. Four SNPs including rs386652275 (Chr2:165,533,198; TC>T; *COBLL1*), rs11057405 (Chr12:122,781,897; A>G; *CLIP1*), rs74841570 (Chr12:124,407,903; C>A; *DNAH10*), and rs11057402 (Chr12:124,430,767; A>T; *CCDC92*) were associated with GFAT. For all 4 SNPs, the BFPAdj increasing allele associated with increased GFAT and improved lipids (higher HDL and lower TG).

The BFPAdj lowering allele of rs13303359 (Chr1: 203,518,873, C>A; *OPTC*) was associated with lower ASAT, lower HDL and higher TG (**Table 2**, **Figure 5B**).

#### Chr X

930,172 SNPs on Chr X were tested for association with BFPAdj. There were 468 GWS SNPs all in one locus (rs12011976: Chr23:109,836,588) (**Figure S7B**; **Supplementary Females.xlsx File, Sheets F, G**). This locus has been associated with SHBG, testosterone and adiposity related phenotypes previously. It was also associated with BFPAdj in males.

#### HLA imputation

None of the HLA haplotypes reached GWS threshold (**Supplementary Females.xlsx File, Sheet H**).

#### MR

The MR Egger method showed significant positive causal effect of BFPAdj on HDL (β (SE) = 0.047 (0.022), p = 0.028) with significant evidence for directional pleiotropy (p = 0.017) and heterogeneity (p< 2E-110). However, this result and the direction of effect was not supported by the other four MR methods. Both weighted median (β (SE) = -0.013 (0.003), p = 1.54E-5) and simple mode (β (SE) = -0.038 (0.010), p = 3.75E-4) showed that BFPAdj has negative causal effect on HDL. IVW and weighted mode did not show any significant causal effects but the direction of effect was consistent with weighted median and simple mode methods (**Supplementary Females.xlsx File, Sheets K, L**).

The MR Egger method did not show any significant causal effect for BFPAdj on T2D (p = 0.65), CAD (p = 0.86) or TG (p = 0.066). Some, but not all, of the other methods showed significant associations but collectively we did not find any reliable evidence that increased BFPAdj increases risk of T2D, CAD or TG levels; and there was highly significant evidence for heterogeneity (p< 2E-75). There was no evidence for directional pleiotropy (p >0.05) (**Supplementary Females.xlsx File, Sheets K, L**).

Similar to males, single SNP MR results demonstrated three groups of SNPs suggesting positive, negative and no significant causal effect of BFPAdj on the four outcomes consistent with high levels of heterogeneity.

None of the SNPs made significant difference in MR analysis results of T2D, CAD, HDL or TG in the leave-one-out analysis (**Supplementary Females MR Plots**). No SNP was deemed an outlier instrument by the MR-PRESSO horizontal pleiotropic outlier detection. All instruments withstood Steiger filtering.

Using the LCV model, we found positive genetic correlations between BFPAdj and T2D and CAD, and a negative genetic correlation between BFPAdj and HDL, yet no evidence suggested causal effect of BFPAdj on these traits (all estimated gcp<0.6; **Table S8**).

### SNP x sex interaction GWAS

312,274 subjects and 28,442,141 SNPs were included in the GWAS (GC lambda for SNP x Sex = 1.03). Only 25 SNPs in 4 loci reached the GWS threshold: rs754823863, rs16885587, rs5030937 and rs55745760 (**Figure 3C**, **Table S9**). rs754823863 and rs55745760 were not GWS associated with BFPAdj in males or females whereas rs16885587 and rs5030937 were associated with BFPAdj in only females (**Table S10**).

## Discussion

We performed sex-stratified GWAS of BFPAdj in the UK Biobank including SHBG and testosterone in the model. Our data suggests that 1. This approach increases the power to detect BFP associated loci. 2. The majority of BFPAdj loci do not overlap between sexes at GWS 3. BFPAdj loci generally do not appear to have significant deleterious cardiometabolic effects.

### Genetic determinants of BFPAdj

We identified 193 autosomal loci in males and 174 autosomal loci in females associated with BFPAdj a significant increase from the 12 loci identified in a previously published BFP GWAS (12). The identified loci explained 3.35% and 2.60% of the variation in BFPAdj in males and females, respectively; whereas 12 previously identified loci explained only 0.57% of the variation in BFP (12). Of these, 94 loci in males and 104 loci in females were not identified in unpublished analyses by the Neale lab. Seven loci in males (including two on Chr X) and 10 in females have not been associated with any adiposity related/cardiometabolic traits previously. Despite adjustment for SHBG and testosterone, a minority of loci (38 loci) were associated with BFPAdj in both sexes at GWS, underscoring the differential genetic regulators of BFPAdj by sex.

Eight out of twelve previously reported loci for BFP (12) were associated with BFPAdj in both males and females in our analyses. Another locus, *IRS1* (insulin receptor substrate 1), was only associated with BFPAdj in males. The other three loci were not replicated in males or females: *SPRY2*, *IGF2BP1* and *CRTC1*. There were multiple independent signals in *FTO* for males and in *COBLL1* for females (**Table S11**).

### Association of BFPAdj loci with cardiometabolic disease

Intriguingly, the majority of identified BFPAdj loci in males and females did not associate with lipid levels or cardiometabolic diseases such as T2D (13 out of 193 and 4 out of 174 in males and females, respectively) or CAD (5 and 3 in males and females, respectively). A number of BFPAdj increasing alleles, were paradoxically associated with higher HDL and lower TG, and in some cases reduced risk of T2D and CAD. They were also often associated with lower WHR and in some cases increased GFAT. In our primary MR analysis, we employed the MR Egger method to investigate the potential causal effect of BFPAdj on HDL, TG, T2D, and CAD, since this method can detect and account for directional pleiotropy (41). The results obtained through the MR Egger method did not provide substantial evidence to support a causal relationship between BFPAdj and these health outcomes. This finding aligns with the results from the LCV model, which also detected genetic correlation but did not provide support for a causal link. Notably, although significant results were produced by alternative MR methods, such as the weighted median, simple mode, and weighted mode methods, these methods impose the condition that the majority of the instruments should not be influenced by horizontal pleiotropy, which is an elusive assumption for exposures with a polygenic architecture. Additionally, the significant variations observed in the estimated causal effects across different genetic instruments used in the analysis raise concerns about the potential violation of instrumental variable assumptions, which could introduce bias into these methods. Taken together, consistent with previous data, our findings based on the MR Egger method and LCV model indicate that higher BFPAdj, in the absence of deleterious fat distribution, likely does not increase cardiometabolic risk. It is also likely indicative of the contribution of testosterone/SHBG, which are not associated with these variants, to cardiometabolic phenotypes (13, 14).

Previous genetic studies have not reported adverse metabolic effects of reduced ASAT (10). At the *OPTC* locus the BFPAdj lowering allele associated with reduced ASAT, lower HDL and higher TG. Whether the metabolic effects are due to reduced ASAT is not established. At another locus on Chr19 between *CEBPG* and *CEBPA*, a variant associated with higher BFPAdj and VAT in females but not males was also associated with higher HDL levels (in both sexes) potentially suggesting the HDL increase is independent of fat distribution. At *APOC1*-*APOE* locus, we observed BFPAdj increasing alleles which were associated with both lower HDL, lower TG and higher risk of CAD. This locus has been associated with total and LDL cholesterol as well as statin use, which may influence the association with CAD (42, 43).

### Gene expression analyses

We investigated whether novel identified SNPs affected gene expression in different tissues based on the Genotype-Tissue Expression (GTEx) project v8 (44, 45) with a more specific focus in visceral adipose tissue. We report that the novel loci did not affect expression of the genes that SNPs are located in or the nearest genes to the SNP (**Table S12**). For example, rs2941584 (Chr2: 54,881,621) is an intronic SNP within *SPTBN1* and is an expression quantitative trait loci (eQTL) for another gene in its vicinity, *EML6* which might be a more relevant gene as variants within *EML6* have been associated with extreme obesity previously (46). Another example is rs4962671 (Chr10:126,305,434) an intergenic SNP between *FAM53B* and *LHPP* which is an eQTL in visceral adipose tissue for *METTL10* (Chr10:126,447,406-126,480,439) ~142 kb away. rs56385874 (Chr19: 47,282,245) is also an intronic SNP within *SLC1A5* but it is eQTL in visceral adipose tissue for two other genes: *FKRP* and *PRKD2* 20 and 62 kb away from the SNP, respectively. This data highlights candidate genes for further functional studies.

### Limitations

Our study had some limitations. The participation rate in UK Biobank at baseline was 5.5% of invitees, with higher participation rate in females than males, and participants were less likely to be obese and more healthier than the general population (47). Mean and variance of BFP were different in males and females. Therefore, for SNPs with similar effect size in both sexes, a larger sample size was required to detect the association in females than males. For example, the required sample size (power >80% & α = 0.05) to detect association of a SNP with MAF of 20% and effect estimate of 1 was 879 and 1,198 in males and females, respectively. It is likely that BFP was influenced by menopause status. The UK Biobank age ranged between 40-70 years and ~72% of the females had undergone either hysterectomy or menopause at baseline (UDI 2724-0.0) of whom ~50% had a history of hormone replacement therapy (HRT) use. More in-depth studies stratified by menopause status and HRT use will be informative. We did not include estradiol as a covariate in the model for females because the majority of them had the values that were either above or below the detection limit (48). These could result in biased estimates. We excluded any SNP associated with SHBG or testosterone at GWS to avoid collider bias (27). However, there might be loci that were associated with SHBG or testosterone but did not reach this level of significance due to lack of statistical power. Nevertheless, of the 17 newly identified loci (**Table 1**), the majority were not associated with SHBG or testosterone at all (p<0.05) and only four were nominally associated with either SHBG or testosterone (p<0.001) (**Table S7**) suggesting there is little evidence for collider bias. Using this approach, we avoided collider bias to achieve our aim of identification of loci associated with BFP independent of SHBG or testosterone. However, we could not identify loci with pleiotropic effect affecting multiple correlated phenotypes i.e. SHBG, testosterone and BFP. Multivariate approaches can help to identify these loci and are more powerful than the standard GWAS approach of analyzing one phenotype at a time (49, 50). ASAT, VAT, GFAT sample sizes were relatively small, therefore low statistical power could produce false negative results.

## Conclusion

By undertaking sex-stratified analyses adjusted for SHBG and testosterone, we have identified several novel BFPAdj associated loci with limited overlap between sexes at GWS. Further, the majority of BFPAdj increasing alleles at identified loci did not associate with adverse cardiometabolic parameters. MR analyses did not provide convincing evidence that that increased BFPAdj has deleterious cardiometabolic effects which likely underscores the contribution of SHBG and testosterone to fat distribution and cardiometabolic traits.

## Data availability statement

Publicly available datasets were analyzed in this study. This data can be found here: This research has been conducted using the UK Biobank Resource under Application Number 48873. The summary stats for BFPAdj GWAS in males and females are available on MY.LOCUSZOOM.ORG: https://my.locuszoom.org/gwas/915413/?token=afa2e57d233c48ff972be83bf4360881 https://my.locuszoom.org/gwas/204024/?token=0fb3b984ef1f42a796a3b62de51b5daa They also will become available in the NHGRI-EBI GWAS catalogue upon publication.

## Ethics statement

The studies involving humans were approved by The Hospital for Sick Children. The studies were conducted in accordance with the local legislation and institutional requirements. The participants provided their written informed consent to participate in this study.

## Author contributions

DR: Conceptualization, Data curation, Formal Analysis, Investigation, Methodology, Visualization, Writing – original draft, Writing – review & editing. TL: Formal Analysis, Investigation, Methodology, Writing – original draft. AP: Conceptualization, Funding acquisition, Investigation, Methodology, Project administration, Resources, Supervision, Writing – review & editing. SD: Conceptualization, Funding acquisition, Investigation, Methodology, Project administration, Resources, Supervision, Writing – original draft, Writing – review & editing.
