# Supplementary material for: Beyond apples and pears: sex-specific genetics of body fat percentage
Source: Front Endocrinol (Lausanne). 2023 Oct 5;14:1274791. doi: 10.3389/fendo.2023.1274791 (PMC10585153; doi:10.3389/fendo.2023.1274791)
Supplement: Supplementary file 1 [file DataSheet_1.pdf]

Supplementary Material

Table of Contents:

	Page
**Links to Supplementary Excel Files Males.xlsx and Females.xlsx**	3
**Supplementary Tables**	4
**Table S1:** The list of medical treatment (Unique Data Identifier (UDI) 20003-0.0) that subjects on them were excluded from the analysis	4
**Table S2:** The GWAS used to extract association of SNPs with SHBG, testosterone, TG, HDL, T2D, CAD, VAT, ASAT, and GFAT	6
**Table S3:** Subjects characteristics	7
**Table S4:** Association of covariates with BFP in males and females	8
**Table S5:** Correlation between covariates and BFP	9
**Table S6:** Association of novel BFPAdj GWAS loci with MRI-derived ASAT, VAT and GFAT adjusted for BMI and height	10
**Table S7:** Association of novel BFPAdj GWAS loci with BFP before and after including albumin, SHBG and testosterone, and their quadratic terms in the model as well as their association with albumin, SHBG and testosterone	11
**Table S8:** Estimated genetic causality proportion (gcp) using LCV model	12
**Table S9:** SNPs interacting with sex affecting BFPAdj	13
**Table S10:** Independent SNPs interacting with sex affecting BFPAdj	14
**Table S11:** The SNPs associated with BFP in Lu et al 2016 paper	15
**Table S12:** Novel loci that are eQTLs based on GTEx v8	16
**Table S13:** Association of rs7133378 & rs1716407 and rs56361048 & rs7258937 with BFPAdj in males when both SNPs were in the model	20
**Table S14:** Association of rs74841570 and rs11057402 with BFPAdj in females when both SNPs were in the model	20
**Supplementary Figures**	21
**Figure S1:** Distribution of BFP in males and females	21
**Figure S2:** Association of BFPAdj GWAS SNPs in the current analysis with SHBG in Ruth et al analysis in males (N = 193) and females (N = 174)	22
**Figure S3:** Association of BFPAdj GWAS SNPs in the current analysis with testosterone in Ruth et al analysis in males and females	23
**Figure S4:** The effect sizes of independent BFPAdj GWAS loci in males (N = 193) and females (N = 174)	24
**Figure S5:** The p-values ($-\log_{10}$) of independent BFPAdj GWAS loci in males (N = 193) and females (N = 174)	25
**Figure S6:** Association of BFPAdj GWAS SNPs in the current analysis with BFP in Neale's round 2 GWAS results	26
**Figure S7:** Association of SNPs on Chr X with BFPAdj in males and females	27

	Page
**References**	28
**Males MR Plots**	29
**T2D**	29
**CAD**	34
**HDL**	39
**TG**	44
**Females MR Plots**	49
**T2D**	49
**CAD**	54
**HDL**	59
**TG**	64

Links to Supplementary Excel Files:

Males.xlsx:
https://docs.google.com/spreadsheets/d/1MeewUJ6-cG8vjDpnifpgpn4oPn8JN_mv/edit?usp=sharing&ouid=110612840906643856403&rtpof=true&sd=true

Females.xlsx:

https://docs.google.com/spreadsheets/d/1hlT9ZWtstKA4hWpOpLU4uFfSsBwauGYd/edit?usp=sharing&ouid=110612840906643856403&rtpof=true&sd=true

Supplementary Tables

Table S1: The list of medical treatment (Unique Data Identifier (UDI) 20003-0.0) that subjects on them were excluded from the analysis

Males	Females
1. anastrozole<br>2. arimidex 1mg tablet<br>3. buserelin<br>4. buserelin product<br>5. clomid 50mg tablet<br>6. conjugated oestrogens 0.3mg / medroxyprogesterone 1.5mg tab<br>7. cyclogest 200mg suppository<br>8. cyprostat 50mg tablet<br>9. cyproterone<br>10. cyproterone acetate+ethinyloestradiol<br>11. de-capeptyl sr 4.2mg injection (pdr for recon)+diluent<br>12. deltahydrocortisone<br>13. deltastab 1mg tablet<br>14. dexamethasone<br>15. drogenil<br>16. dydrogesterone<br>17. ethinylnortestosterone<br>18. ethinyloestradiol product<br>19. ethinyloestradiol+norethisterone acetate 20mcg/1mg tablet<br>20. ethinyloestradiol+norgestimate 35mcg/250mcg tablet<br>21. ethynodiol diacetate<br>22. flutamide<br>23. gonadorelin<br>24. goserelin<br>25. goserelin product<br>26. hydrocortisone<br>27. hydrocortisone product<br>28. hydrocortistab 20mg tablet<br>29. hydrocortone 10mg tablet<br>30. letrozole<br>31. leuprorelin<br>32. levonorgestrel<br>33. levonorgestrel product<br>34. loestrin<br>35. medrone 2mg tablet<br>36. medroxyprogesterone<br>37. mestranol+norethisterone 50micrograms/1mg tablet<br>38. methylprednisolone<br>39. methyltestosterone product<br>40. micronor<br>41. microval tablet<br>42. nafarelin<br>43. norethisterone<br>44. norethisterone product<br>45. norgeston tablet<br>46. noriday<br>47. norinyl-1 tablet	1. methyltestosterone product<br>2. testotop tts 15mg transdermal patch<br>3. yohimbine/pemoline/methyltestosterone<br>4. testosterone product<br>5. primoteston depot 250mg/1ml oily injection<br>6. testoderm 6mg/24hours transdermal patch<br>7. testogel 50mg gel 5g sachet<br>8. cyprostat 50mg tablet<br>9. flutamide<br>10. drogenil<br>11. anastrozole<br>12. arimidex 1mg tablet<br>13. letrozole<br>14. cyproterone<br>15. cyproterone acetate+ethinyloestradiol<br>16. cyproterone acetate+ethinylestradiol<br>17. dydrogesterone<br>18. proscar 5mg tablet<br>19. clomid 50mg tablet<br>20. medrone 2mg tablet<br>21. prednisone<br>22. prednisolone<br>23. prednesol 5mg tablet<br>24. hydrocortistab 20mg tablet<br>25. hydrocortone 10mg tablet<br>26. methylprednisolone<br>27. prednisolone product<br>28. hydrocortisone<br>29. deltastab 1mg tablet<br>30. precortisyl 1mg tablet<br>31. deltahydrocortisone<br>32. hydrocortisone product<br>33. dexamethasone

48. noristerat 200mg/1ml oily injection<br>49. ortho-novin 1/50 tablet<br>50. precortisyl 1mg tablet<br>51. prednesol 5mg tablet<br>52. prednisolone<br>53. prednisolone product<br>54. prednisone<br>55. primoteston depot 250mg/1ml oily injection<br>56. progesterone product<br>57. proscar 5mg tablet<br>58. prostap sr 3.75mg injection (pdr for recon)+diluent+kit<br>59. suprefact 100micrograms nasal spray<br>60. testoderm 6mg/24hours transdermal patch<br>61. testogel 50mg gel 5g sachet<br>62. testosterone product<br>63. testotop tts 15mg transdermal patch<br>64. triptorelin<br>65. yohimbine/pemoline/methyltestosterone	

Table S2: The GWAS used to extract association of SNPs with SHBG, testosterone, TG, HDL, T2D, CAD, VAT, ASAT, and GFAT

Trait	First Author	Ethnicity	Includes UKBB	Sex Stratified	Subjects	Link to Download
SHBG	Ruth et al (1)	European	Yes	Yes	180,726 Males<br>189,473 Females	Males: https://www.ebi.ac.uk/gwas/studies/GCST90012109<br>Females: https://www.ebi.ac.uk/gwas/studies/GCST90012107
Testosterone	Ruth et al (1)	European	Yes	Yes	194,453 Males<br>230,454 Females	Males: https://www.ebi.ac.uk/gwas/studies/GCST90012113<br>Females: https://www.ebi.ac.uk/gwas/studies/GCST90012112
HDL & TG	Graham et al (2)	European 80%<br>East Asian 9%<br>African 6%<br>Hispanic 3%<br>South Asian 2%	Yes	Yes	1,654,960<br>51% Females	http://csg.sph.umich.edu/willer/public/glgc-lipids2021/results/sex_specific_summary_stats/
T2D	Mahajan et al (3)	European	Yes	No	74,124 Cases<br>824,006 Controls	http://diagram-consortium.org/downloads.html<br>Mahajan.NatGen2022.DIAMANTE-EUR.sumstat.zip
CAD	Aragam et al (4)	European	Yes	No	181,522 Cases<br>984,168 Controls	http://ftp.ebi.ac.uk/pub/databases/gwas/summary_statistics/GCST90132001-GCST90133000/GCST90132314/
GFAT, VAT & ASAT	Agrawal et al (5)	European 87%	Yes	Yes	38,965<br>51% Females	https://cvd.hugeamp.org/downloads.html#summary
WHR adjusted for BMI	Pulit et al (6)	European	Yes	Yes	315,284 Males<br>379,501 Females	https://zenodo.org/record/1251813#.Y-u7InbMKUl

Table S3: Subjects characteristics

	Males (N = 157,937)		Females (N = 154,337)	
	**Mean**	**SD**	**Mean**	**SD**
**Age (years)**	57.1	8.1	56.3	8.0
**Albumin (g/L)**	45.5	2.6	45.0	2.6
**SHBG (nmol/L)**	39.9	16.7	61.8	30.1
**Testosterone (nmol/L)**	12.0	3.7	1.1	0.6
**Body fat percentage (%)**	25.3	5.8	36.7	6.9

Table S4: Association of covariates with BFP in males and females

	Males				Females			
	**Beta**	**SE**	**P**	**R^2**	**Beta**	**SE**	**P**	**R^2**
**Age**	0.1559	0.0020	<2E-16	0.034	0.1148	0.0002	<2E-16	0.029
**Centered Age^2**	-0.0010	0.0002	1.04E-5		-0.0022	0.0003	<2E-16	
**Albumin**	-0.0562	0.0054	<2E-16	0.004	-0.4242	0.0060	<2E-16	0.026
**Centered Albumin^2**	-0.0012	0.0012	0.33		0.0135	0.0015	<2E-16	
**SHBG**	-0.0768	0.0013	<2E-16	0.049	-0.1142	0.0006	<2E-16	0.183
**Centered SHBG^2**	0.0005	0.00002	<2E-16		0.0007	0.00001	<2E-16	
**Testosterone**	-0.2755	0.005	<2E-16	0.084	1.3180	0.0328	<2E-16	0.006
**Centered Testosterone^2**	0.0121	0.0006	<2E-16		-0.2535	0.0120	<2E-16	
**R^2**	**0.13**				**0.23**			

Multi-variable linear regression was used to test association of covariates with BFP.

Table S5: Correlation between covariates and BFP

A.

	**BFP**	**Albumin**	**SHBG**	**Testosterone**
**BFP**		-0.06	-0.21	-0.28
**Albumin**	<1E-4		-0.11	0.01
**SHBG**	<1E-4	<1E-4		0.58
**Testosterone**	<1E-4	3.15E-02	<1E-4	

B.

	**BFP**	**Albumin**	**SHBG**	**Testosterone**
**BFP**		-0.16	-0.43	0.07
**Albumin**	<1E-5		0.00	0.02
**SHBG**	<1E-5	0.46		-0.02
**Testosterone**	<1E-5	<1E-5	<1E-5	

A: Males, B: Females
Above diagonal: Spearman correlation coefficient; Below diagonal: p-value

Table S6: Association of novel BFPAdj GWAS loci with MRI-derived ASAT, VAT and GFAT adjusted for BMI and height (5)

									Agrawal et al					
					Males		Females		ASAT		VAT		GFAT	
**CHR**	**BP (HG19)**	**SNP**	**A0**	**A1**	**BETA**	**LOG10P**	**BETA**	**LOG10P**	**Beta**	**p**	**Beta**	**p**	**Beta**	**p**
1	106802195	rs11184828	A	G	-0.07	3.09	-0.14	8.65	-0.01	0.21	0.005	0.43	-0.003	0.64
1	150055361	rs116819476	C	T	0.17	2.79	0.33	7.39	0.006	0.71	0.008	0.78	-0.007	0.83
1	222061973	rs11577023	T	C	-0.06	2.66	-0.12	7.32	-0.01	0.17	-0.007	0.28	0.013	0.17
2	54881621	rs2941584	T	C	-0.04	1.26	-0.12	7.68	-0.014	0.059	-0.006	0.58	-0.003	0.62
2	101414496	rs2309885	C	T	0.1	7.44	0.03	0.65	-0.014	0.07	-0.002	0.59	0.023	0.0011
3	30071380	rs7426945	A	G	0.05	2.35	0.12	8.06	0.008	0.21	-0.003	0.54	-0.006	0.44
4	2405062	rs35802140	C	G	0.07	1.79	0.19	8.73	0.005	0.71	0.016	0.083	-0.005	0.66
10	126305434	rs4962671	T	C	-0.1	7.91	-0.03	0.98	0.001	0.96	0.008	0.38	-0.003	0.56
10	131430686	rs524804	G	A	-0.07	3.46	-0.13	8.75	-0.017	0.016	0.001	0.86	0.001	0.82
11	66820856	rs117773425	C	A	0.39	7.68	0	0.01	0.031	0.25	0.006	0.75	0.38	0.22
13	76086882	rs531470369	C	CTTTTTTT	0.03	1.11	0.12	7.39	-	-	-	-	-	-
16	25247974	rs151118254	C	T	0.17	0.85	0.73	7.42	-0.001	0.93	-0.006	0.9	0.009	0.78
19	47282245	rs56385874	C	T	0.05	1.3	0.17	9.32	0.011	0.22	-0.009	0.31	0.019	0.038
20	38490795	rs768147154	T	TAGAG	-0.12	8.1	-0.06	1.77	-0.009	0.34	0.008	0.25	-0.016	0.062
20	55823762	rs6127980	G	A	0.15	7.31	0.03	0.48	-0.007	0.6	-0.001	0.97	0.018	0.13
23	43,017,461	rs5950969	C	T	0.10	7.46	-0.02	0.25	-	-	-	-	-	-
23	83,562,659	rs73505165	G	A	0.07	7.66	-0.00	0.01	-	-	-	-	-	-

A0: reference allele, A1: Effect Allele, ASAT: Abdominal subcutaneous adipose tissue, VAT: Visceral adipose tissue, GFAT: Gluteofemoral adipose tissue

Table S7: Association of novel BFPAdj GWAS loci with BFP before and after including albumin, SHBG and testosterone, and their quadratic terms in the model as well as their association with albumin, SHBG and testosterone

					M0			M1			Albumin			SHBG			Testosterone		
**SNP**	**CHR**	**BP (HG19)**	**A1**	**Sex**	**BETA**	**SE**	**P**	**BETA**	**SE**	**P**	**BETA**	**SE**	**P**	**BETA**	**SE**	**P**	**BETA**	**SE**	**P**
rs11184828	1	106,802,195	A	F	0.121	0.027	7.40E-06	0.158	0.024	4.45E-11	0.013	0.010	0.20	0.269	0.120	2.49E-02	0.000	0.002	0.99
rs116819476	1	150,055,361	T	F	0.272	0.076	3.38E-04	0.300	0.067	8.54E-06	-0.042	0.029	0.15	0.315	0.337	0.35	0.002	0.006	0.80
rs11577023	1	222,061,973	C	F	-0.109	0.027	4.16E-05	-0.107	0.024	5.92E-06	0.022	0.010	2.62E-02	-0.077	0.118	0.52	-0.001	0.002	0.62
rs2941584	2	54,881,621	T	F	0.133	0.026	3.31E-07	0.127	0.023	4.25E-08	-0.012	0.010	0.23	0.001	0.116	1.00	0.000	0.002	0.84
rs2309885	2	101,414,496	T	M	0.095	0.021	3.63E-06	0.095	0.019	1.02E-06	-0.004	0.009	0.62	0.036	0.058	0.54	-0.010	0.013	0.43
rs7426945	3	30,071,380	A	F	-0.126	0.024	2.10E-07	-0.115	0.022	1.03E-07	0.013	0.009	0.17	0.034	0.108	0.75	0.002	0.002	0.35
rs35802140	4	2,405,062	G	F	0.142	0.038	2.14E-04	0.181	0.034	1.09E-07	0.011	0.015	0.45	0.310	0.171	0.07	0.001	0.003	0.81
rs4962671	10	126,305,434	C	M	-0.117	0.020	9.32E-09	-0.101	0.019	1.58E-07	-0.005	0.009	0.56	0.114	0.058	0.05	0.019	0.013	0.15
rs524804	10	131,430,686	A	F	-0.116	0.025	2.51E-06	-0.137	0.022	4.85E-10	-0.013	0.009	0.16	-0.063	0.110	0.57	0.000	0.002	0.91
rs117773425	11	66,820,856	A	M	0.403	0.080	5.38E-07	0.395	0.076	2.18E-07	-0.017	0.036	0.63	-0.126	0.229	0.58	0.004	0.052	0.94
rs531470369	13	76,086,882	C	F	-0.117	0.027	1.14E-05	-0.113	0.024	1.62E-06	0.015	0.010	0.15	-0.013	0.118	0.91	0.005	0.002	4.38E-02
rs151118254	16	25,247,974	T	F	0.638	0.190	7.77E-04	0.594	0.168	4.22E-04	0.070	0.072	0.33	-0.738	0.843	0.38	0.007	0.016	0.65
rs56385874	19	47,282,245	T	F	0.136	0.032	1.75E-05	0.162	0.028	9.94E-09	0.024	0.012	4.62E-02	0.093	0.141	0.51	-0.002	0.003	0.45
rs768147154	20	38,490,795	T	M	0.084	0.023	2.89E-04	0.111	0.022	5.19E-07	-0.030	0.010	3.95E-03	0.187	0.066	4.75E-03	0.043	0.015	4.49E-03
rs6127980	20	55,823,762	A	M	0.136	0.031	1.59E-05	0.147	0.030	7.26E-07	-0.020	0.014	0.14	0.070	0.089	0.44	0.041	0.020	4.00E-02
rs5950969	23	43,017,461	C	M	-0.090	0.021	1.34E-05	-0.097	0.020	6.95E-07	0.025	0.009	6.43E-03	-0.058	0.059	0.32	-0.015	0.013	0.26
rs73505165	23	83,562,659	G	M	-0.078	0.014	5.93E-08	-0.075	0.014	3.84E-08	0.008	0.006	0.19	0.015	0.041	0.71	0.003	0.009	0.72

Linear regression was used to test association of SNPs with BFP, albumin, SHBG and testosterone.
M0: Age, Age^2, PC1, PC2, PC3, PC4, PC5, PC6, PC7, PC8, PC9 and PC10 were included as covariates in the model.
M1: All covariates in M0 plus albumin, albumin^2, SHBG, SHBG^2, testosterone and testosterone^2 were included as covariates in the model.
Age, Age^2, PC1, PC2, PC3, PC4, PC5, PC6, PC7, PC8, PC9 and PC10 were included as covariates in the models to test association of SNPs with albumin, SHBG and testosterone.
A1: Effect allele, M: Males, F: Females

Table S8: Estimated genetic causality proportion (gcp) using LCV model

Sex	Exposure	Outcome	Genetic correlation (SE)	gcp (SE)
Male	BFPAdj	CAD	0.24 (0.03)	-0.65 (0.24)
Male	BFPAdj	T2D	0.36 (0.04)	-0.04 (0.58)
Male	BFPAdj	HDL	-0.23 (0.04)	-0.69 (0.21)
Male	BFPAdj	TG	0.06 (0.05)	-0.19 (0.14)
Female	BFPAdj	CAD	0.21 (0.04)	-0.53 (0.31)
Female	BFPAdj	T2D	0.32 (0.04)	-0.06 (0.53)
Female	BFPAdj	HDL	-0.20 (0.04)	0.36 (0.30)
Female	BFPAdj	TG	0.12 (0.04)	0.09 (0.20)

Table S9: SNPs interacting with sex affecting BFPAdj

CHR	BP (HG19)	ID	A0	A1	A1FREQ	INFO	BETA	SE	LOG10P
4	62,634,106	rs754823863	T	C	0.999	0.730	3.600	0.650	7.52
8	36,794,458	rs16885587	C	T	0.872	0.977	0.229	0.041	7.54
8	36,829,456	rs61146997	CTAAA	C	0.814	0.948	0.197	0.036	7.38
8	36,846,435	rs75772194	G	A	0.836	0.999	0.202	0.037	7.36
8	36,847,115	rs10110651	C	T	0.836	1.000	0.203	0.037	7.43
8	36,847,709	rs10086016	C	T	0.836	1.000	0.204	0.037	7.49
8	36,848,038	rs28735403	C	T	0.836	0.999	0.203	0.037	7.43
8	36,848,357	rs16885613	C	T	0.836	0.999	0.203	0.037	7.42
8	36,849,946	rs4286946	G	C	0.836	0.998	0.202	0.037	7.33
8	36,851,901	rs10955009	A	C	0.836	0.998	0.203	0.037	7.44
8	36,853,213	rs10096213	T	C	0.836	0.995	0.204	0.037	7.48
8	36,853,217	rs10095380	C	G	0.836	0.995	0.204	0.037	7.48
10	70,975,897	rs5030937	T	C	0.308	0.994	-0.168	0.030	7.82
10	70,975,916	rs5030938	T	C	0.308	0.994	-0.167	0.030	7.80
10	70,976,833	rs10762264	A	G	0.309	0.995	-0.166	0.030	7.68
10	70,977,308	rs10998647	G	T	0.308	0.995	-0.166	0.030	7.66
10	70,977,395	rs10998648	C	A	0.308	0.996	-0.167	0.030	7.73
10	70,979,924	rs10823318	T	A	0.309	0.996	-0.166	0.030	7.67
10	70,982,136	rs35696875	TCA	T	0.301	0.997	-0.163	0.030	7.37
10	70,982,941	rs4746822	T	C	0.301	0.999	-0.163	0.030	7.38
10	70,983,629	rs9663238	G	A	0.301	0.999	-0.164	0.030	7.44
10	70,983,936	rs35199395	C	G	0.301	0.998	-0.164	0.030	7.45
10	70,985,267	rs2394529	C	G	0.301	0.999	-0.163	0.030	7.41
10	70,986,723	rs9645500	G	T	0.301	1.000	-0.164	0.030	7.47
17	7,568,925	rs55745760	T	C	0.597	0.960	-0.165	0.029	8.15

Table S10: Independent SNPs interacting with sex affecting BFPAdj

						All (SNP x Sex Interaction)					Males					Females				
**CHR**	**BP (HG19)**	**SNP**	**Gene**	**A0**	**A1**	**A1FREQ**	**INFO**	**BETA**	**SE**	**LOG10P**	**A1FREQ**	**INFO**	**BETA**	**SE**	**LOG10P**	**A1FREQ**	**INFO**	**BETA**	**SE**	**LOG10P**
4	62,634,106	rs754823863	ADGRL3	T	C	0.999	0.73	3.60	0.65	7.52	0.999	0.73	-1.56	0.44	3.41	0.999	0.73	1.96	0.48	4.33
8	36,794,458	rs16885587	KCNU1	C	T	0.872	0.98	0.23	0.04	7.54	0.872	0.98	0.01	0.03	0.09	0.872	0.98	0.22	0.03	12.07
10	70,975,897	rs5030937	HKDC1	T	C	0.308	0.99	-0.17	0.03	7.82	0.308	0.99	0.05	0.02	1.89	0.308	0.99	-0.10	0.02	5.45
17	7,568,925	rs55745760	TP53	T	C	0.597	0.96	-0.17	0.03	8.15	0.597	0.96	0.01	0.02	0.11	0.596	0.96	-0.16	0.02	12.97

Table S11: The SNPs associated with BFP in Lu et al 2016 paper (7)

					**Males**		**Females**	
**SNP**	**CHR**	**BP (HG18)**	**BP (HG19)**	**Gene**	**Top SNP**	**BP (HG19)**	**Top SNP**	**BP (HG19)**
rs543874	1	176,156,103	177,889,480	SEC16B	rs539515	177,889,025	rs543874	177,889,480
rs6755502	2	625,721	635,721	TMEM18	rs4407278	648,607	rs6751993	635,864
rs6738627	2	165,252,696	165,544,450	COBLL1	rs1128249	165,528,624	rs200472737*	165,544,573
rs2943652	2	226,816,690	227,108,446	IRS1	rs2943650	227,105,921	**No Signal**	-
rs693839	13	79,856,289	80,958,288	SPRY2	**No Signal**	-	**No Signal**	-
rs4788099	16	28,763,228	28,855,727	TUFM	16:28579915_GT_G	28,579,915	rs437564	28,818,037
rs1558902	16	52,361,075	53,803,574	FTO	rs56094641*	53,806,453	rs1421085	53,800,954
rs9906944	17	44,446,419	47,091,420	IGF2BP1	**No Signal**	-	**No Signal**	-
rs6567160	18	55,980,115	57,829,135	MC4R	rs73455668	57,985,366	rs11451426	60,189,009
rs757318	19	18,681,308	18,820,308	CRTC1	**No Signal**	-	**No Signal**	-
rs6857	19	50,084,094	45,392,254	TOMM40	rs190712692	45,425,178	rs7412	45,412,079
rs3761445	22	36,925,357	38,595,411	PLA2G6	rs4821764	38,599,364	rs4820325	38,599,978

* There are other independent signals in the locus.

Table S12: Novel loci that are eQTLs based on GTEx v8

Gencode Id	Gene Symbol	Variant Id	SNP Id	P-Value	NES	Tissue
ENSG00000266472.5	MRPS21	chr1_150083277_C_T_b38	rs116819476	0.0000048	0.36	Adipose - Visceral (Omentum)
ENSG00000143369.14	ECM1	chr1_150083277_C_T_b38	rs116819476	0.000085	-0.26	Artery - Tibial
ENSG00000266472.5	MRPS21	chr1_150083277_C_T_b38	rs116819476	0.00029	0.22	Artery - Tibial
ENSG00000143369.14	ECM1	chr1_150083277_C_T_b38	rs116819476	0.000056	0.63	Brain - Cerebellar Hemisphere
ENSG00000143369.14	ECM1	chr1_150083277_C_T_b38	rs116819476	6.40E-08	0.92	Brain - Cerebellum
ENSG00000143369.14	ECM1	chr1_150083277_C_T_b38	rs116819476	5.60E-09	0.49	Cells - Cultured fibroblasts
ENSG00000143363.15	PRUNE1	chr1_150083277_C_T_b38	rs116819476	0.00011	0.33	Cells - Cultured fibroblasts
ENSG00000143382.14	ADAMTSL4	chr1_150083277_C_T_b38	rs116819476	0.00024	0.21	Cells - Cultured fibroblasts
ENSG00000266472.5	MRPS21	chr1_150083277_C_T_b38	rs116819476	0.00057	0.28	Cells - Cultured fibroblasts
ENSG00000143369.14	ECM1	chr1_150083277_C_T_b38	rs116819476	3.50E-12	1.6	Cells - EBV-transformed lymphocytes
ENSG00000266472.5	MRPS21	chr1_150083277_C_T_b38	rs116819476	1.80E-07	0.48	Colon - Sigmoid
ENSG00000266472.5	MRPS21	chr1_150083277_C_T_b38	rs116819476	0.00021	0.31	Esophagus - Mucosa
ENSG00000266472.5	MRPS21	chr1_150083277_C_T_b38	rs116819476	1.50E-07	0.41	Esophagus - Muscularis
ENSG00000266472.5	MRPS21	chr1_150083277_C_T_b38	rs116819476	0.000015	0.36	Heart - Atrial Appendage
ENSG00000143374.16	TARS2	chr1_150083277_C_T_b38	rs116819476	0.000037	-0.26	Heart - Left Ventricle
ENSG00000266472.5	MRPS21	chr1_150083277_C_T_b38	rs116819476	0.00001	0.31	Nerve - Tibial
ENSG00000228126.1	FALEC	chr1_150083277_C_T_b38	rs116819476	0.00024	0.49	Nerve - Tibial
ENSG00000266472.5	MRPS21	chr1_150083277_C_T_b38	rs116819476	0.000032	0.76	Pituitary
ENSG00000143369.14	ECM1	chr1_150083277_C_T_b38	rs116819476	0.0000049	0.34	Thyroid
ENSG00000163141.18	BNIPL	chr1_150083277_C_T_b38	rs116819476	0.000063	0.46	Thyroid
ENSG00000143369.14	ECM1	chr1_150083277_C_T_b38	rs116819476	0.0000021	0.51	Whole Blood
ENSG00000238042.5	RP11-815M8.1	chr1_221888631_T_C_b38	rs11577023	2.10E-16	0.46	Lung
ENSG00000238042.5	RP11-815M8.1	chr1_221888631_T_C_b38	rs11577023	0.00012	0.21	Nerve - Tibial
ENSG00000214595.11	EML6	chr2_54654484_T_C_b38	rs2941584	1.10E-17	0.4	Artery - Tibial
ENSG00000214595.11	EML6	chr2_54654484_T_C_b38	rs2941584	5.80E-16	0.46	Esophagus - Muscularis
ENSG00000115306.15	SPTBN1	chr2_54654484_T_C_b38	rs2941584	1.80E-14	-0.2	Whole Blood
ENSG00000214595.11	EML6	chr2_54654484_T_C_b38	rs2941584	2.60E-11	0.39	Artery - Aorta
ENSG00000214595.11	EML6	chr2_54654484_T_C_b38	rs2941584	2.10E-08	0.48	Artery - Coronary
ENSG00000237887.1	RPL23AP32	chr2_54654484_T_C_b38	rs2941584	8.60E-08	-0.25	Lung
ENSG00000214595.11	EML6	chr2_54654484_T_C_b38	rs2941584	1.60E-07	0.35	Esophagus - Gastroesophageal Junction
ENSG00000238018.2	AC093110.3	chr2_54654484_T_C_b38	rs2941584	2.60E-07	0.13	Artery - Tibial
ENSG00000238018.2	AC093110.3	chr2_54654484_T_C_b38	rs2941584	3.10E-07	0.18	Muscle - Skeletal
ENSG00000237887.1	RPL23AP32	chr2_54654484_T_C_b38	rs2941584	4.80E-07	-0.22	Thyroid
ENSG00000238018.2	AC093110.3	chr2_54654484_T_C_b38	rs2941584	8.00E-07	0.15	Skin - Sun Exposed (Lower leg)
ENSG00000237887.1	RPL23AP32	chr2_54654484_T_C_b38	rs2941584	0.0000013	-0.45	Brain - Anterior cingulate cortex (BA24)
ENSG00000115306.15	SPTBN1	chr2_54654484_T_C_b38	rs2941584	0.0000017	0.11	Skin - Sun Exposed (Lower leg)
ENSG00000214595.11	EML6	chr2_54654484_T_C_b38	rs2941584	0.0000018	0.28	Colon - Sigmoid
ENSG00000214595.11	EML6	chr2_54654484_T_C_b38	rs2941584	0.000003	0.27	Adipose - Visceral (Omentum)
ENSG00000237887.1	RPL23AP32	chr2_54654484_T_C_b38	rs2941584	0.0000034	-0.34	Brain - Cerebellar Hemisphere
ENSG00000115306.15	SPTBN1	chr2_54654484_T_C_b38	rs2941584	0.000005	0.12	Skin - Not Sun Exposed (Suprapubic)
ENSG00000237887.1	RPL23AP32	chr2_54654484_T_C_b38	rs2941584	0.0000065	-0.34	Testis
ENSG00000237887.1	RPL23AP32	chr2_54654484_T_C_b38	rs2941584	0.0000071	-0.22	Adipose - Visceral (Omentum)
ENSG00000238018.2	AC093110.3	chr2_54654484_T_C_b38	rs2941584	0.0000098	0.11	Nerve - Tibial
ENSG00000238018.2	AC093110.3	chr2_54654484_T_C_b38	rs2941584	0.000016	0.11	Adipose - Subcutaneous
ENSG00000115306.15	SPTBN1	chr2_54654484_T_C_b38	rs2941584	0.000018	-0.14	Cells - Cultured fibroblasts
ENSG00000237887.1	RPL23AP32	chr2_54654484_T_C_b38	rs2941584	0.000027	-0.22	Adipose - Subcutaneous
ENSG00000214595.11	EML6	chr2_54654484_T_C_b38	rs2941584	0.000032	-0.19	Thyroid
ENSG00000238018.2	AC093110.3	chr2_54654484_T_C_b38	rs2941584	0.000038	0.11	Esophagus - Gastroesophageal Junction
ENSG00000238018.2	AC093110.3	chr2_54654484_T_C_b38	rs2941584	0.000072	0.11	Thyroid
ENSG00000115306.15	SPTBN1	chr2_54654484_T_C_b38	rs2941584	0.0001	0.14	Muscle - Skeletal
ENSG00000214595.11	EML6	chr2_54654484_T_C_b38	rs2941584	0.00016	-0.14	Skin - Sun Exposed (Lower leg)
ENSG00000238018.2	AC093110.3	chr2_54654484_T_C_b38	rs2941584	0.00024	0.099	Lung
ENSG00000159733.13	ZFYVE28	chr4_2403335_C_G_b38	rs35802140	5.40E-14	-0.55	Colon - Sigmoid
ENSG00000159733.13	ZFYVE28	chr4_2403335_C_G_b38	rs35802140	1.10E-11	-0.43	Esophagus - Muscularis

Gencode Id	Gene Symbol	Variant Id	SNP Id	P-Value	NES	Tissue
ENSG00000159733.13	ZFYVE28	chr4_2403335_C_G_b38	rs35802140	5.50E-11	-0.56	Heart - Atrial Appendage
ENSG00000159733.13	ZFYVE28	chr4_2403335_C_G_b38	rs35802140	8.20E-09	-0.33	Thyroid
ENSG00000159733.13	ZFYVE28	chr4_2403335_C_G_b38	rs35802140	2.20E-08	-0.55	Prostate
ENSG00000159733.13	ZFYVE28	chr4_2403335_C_G_b38	rs35802140	3.30E-07	-0.4	Heart - Left Ventricle
ENSG00000159733.13	ZFYVE28	chr4_2403335_C_G_b38	rs35802140	4.60E-07	-0.36	Esophagus - Gastroesophageal Junction
ENSG00000159733.13	ZFYVE28	chr4_2403335_C_G_b38	rs35802140	0.0000011	-0.3	Adipose - Visceral (Omentum)
ENSG00000206113.10	CFAP99	chr4_2403335_C_G_b38	rs35802140	0.0000047	-0.26	Thyroid
ENSG00000159733.13	ZFYVE28	chr4_2403335_C_G_b38	rs35802140	0.0000059	-0.25	Artery - Tibial
ENSG00000159733.13	ZFYVE28	chr4_2403335_C_G_b38	rs35802140	0.000023	-0.27	Lung
ENSG00000063978.15	RNF4	chr4_2403335_C_G_b38	rs35802140	0.000024	0.11	Cells - Cultured fibroblasts
ENSG00000063978.15	RNF4	chr4_2403335_C_G_b38	rs35802140	0.00003	0.25	Colon - Sigmoid
ENSG00000159733.13	ZFYVE28	chr4_2403335_C_G_b38	rs35802140	0.00015	-0.2	Adipose - Subcutaneous
ENSG00000203791.14	METTL10	chr10_124616865_T_C_b38	rs4962671	3.80E-29	0.3	Cells - Cultured fibroblasts
ENSG00000203791.14	METTL10	chr10_124616865_T_C_b38	rs4962671	5.40E-28	0.32	Esophagus - Mucosa
ENSG00000203791.14	METTL10	chr10_124616865_T_C_b38	rs4962671	1.50E-27	0.31	Artery - Tibial
ENSG00000203791.14	METTL10	chr10_124616865_T_C_b38	rs4962671	2.30E-27	0.32	Adipose - Subcutaneous
ENSG00000203791.14	METTL10	chr10_124616865_T_C_b38	rs4962671	3.50E-26	0.3	Thyroid
ENSG00000203791.14	METTL10	chr10_124616865_T_C_b38	rs4962671	2.00E-24	0.38	Breast - Mammary Tissue
ENSG00000203791.14	METTL10	chr10_124616865_T_C_b38	rs4962671	6.20E-22	0.33	Lung
ENSG00000203791.14	METTL10	chr10_124616865_T_C_b38	rs4962671	1.60E-21	0.31	Adipose - Visceral (Omentum)
ENSG00000203791.14	METTL10	chr10_124616865_T_C_b38	rs4962671	1.30E-20	0.36	Artery - Aorta
ENSG00000203791.14	METTL10	chr10_124616865_T_C_b38	rs4962671	6.80E-20	0.26	Skin - Sun Exposed (Lower leg)
ENSG00000203791.14	METTL10	chr10_124616865_T_C_b38	rs4962671	4.30E-19	0.22	Muscle - Skeletal
ENSG00000203791.14	METTL10	chr10_124616865_T_C_b38	rs4962671	1.30E-17	0.26	Skin - Not Sun Exposed (Suprapubic)
ENSG00000203791.14	METTL10	chr10_124616865_T_C_b38	rs4962671	1.10E-16	0.3	Colon - Transverse
ENSG00000203791.14	METTL10	chr10_124616865_T_C_b38	rs4962671	3.00E-15	0.27	Testis
ENSG00000107902.13	LHPP	chr10_124616865_T_C_b38	rs4962671	5.60E-15	-0.15	Nerve - Tibial
ENSG00000203791.14	METTL10	chr10_124616865_T_C_b38	rs4962671	7.80E-14	0.33	Stomach
ENSG00000203791.14	METTL10	chr10_124616865_T_C_b38	rs4962671	8.60E-14	0.24	Nerve - Tibial
ENSG00000203791.14	METTL10	chr10_124616865_T_C_b38	rs4962671	5.10E-13	0.21	Esophagus - Muscularis
ENSG00000203791.14	METTL10	chr10_124616865_T_C_b38	rs4962671	3.10E-12	0.33	Heart - Left Ventricle
ENSG00000203791.14	METTL10	chr10_124616865_T_C_b38	rs4962671	3.20E-12	0.33	Prostate
ENSG00000203791.14	METTL10	chr10_124616865_T_C_b38	rs4962671	3.30E-12	0.36	Liver
ENSG00000203791.14	METTL10	chr10_124616865_T_C_b38	rs4962671	1.10E-10	0.27	Heart - Atrial Appendage
ENSG00000203791.14	METTL10	chr10_124616865_T_C_b38	rs4962671	1.10E-10	0.41	Small Intestine - Terminal Ileum
ENSG00000203791.14	METTL10	chr10_124616865_T_C_b38	rs4962671	1.50E-10	0.45	Cells - EBV-transformed lymphocytes
ENSG00000203791.14	METTL10	chr10_124616865_T_C_b38	rs4962671	1.60E-10	0.35	Artery - Coronary
ENSG00000203791.14	METTL10	chr10_124616865_T_C_b38	rs4962671	2.80E-10	0.14	Whole Blood
ENSG00000203791.14	METTL10	chr10_124616865_T_C_b38	rs4962671	3.00E-10	0.34	Adrenal Gland
ENSG00000203791.14	METTL10	chr10_124616865_T_C_b38	rs4962671	5.30E-10	0.32	Brain - Caudate (basal ganglia)
ENSG00000203791.14	METTL10	chr10_124616865_T_C_b38	rs4962671	8.20E-10	0.35	Pituitary
ENSG00000203791.14	METTL10	chr10_124616865_T_C_b38	rs4962671	3.00E-09	0.21	Esophagus - Gastroesophageal Junction
ENSG00000203791.14	METTL10	chr10_124616865_T_C_b38	rs4962671	5.30E-09	0.33	Brain - Nucleus accumbens (basal [illegible]
ENSG00000203791.14	METTL10	chr10_124616865_T_C_b38	rs4962671	6.70E-09	0.43	Brain - Amygdala
ENSG00000203791.14	METTL10	chr10_124616865_T_C_b38	rs4962671	7.90E-09	0.35	Brain - Cortex
ENSG00000203791.14	METTL10	chr10_124616865_T_C_b38	rs4962671	8.30E-09	0.35	Spleen
ENSG00000203791.14	METTL10	chr10_124616865_T_C_b38	rs4962671	2.80E-08	0.2	Colon - Sigmoid
ENSG00000203791.14	METTL10	chr10_124616865_T_C_b38	rs4962671	3.20E-08	0.41	Vagina
ENSG00000203791.14	METTL10	chr10_124616865_T_C_b38	rs4962671	2.00E-07	0.32	Brain - Putamen (basal ganglia)
ENSG00000203791.14	METTL10	chr10_124616865_T_C_b38	rs4962671	2.70E-07	0.27	Pancreas
ENSG00000203791.14	METTL10	chr10_124616865_T_C_b38	rs4962671	5.80E-07	0.29	Minor Salivary Gland
ENSG00000203791.14	METTL10	chr10_124616865_T_C_b38	rs4962671	0.0000065	0.24	Brain - Hypothalamus
ENSG00000203791.14	METTL10	chr10_124616865_T_C_b38	rs4962671	0.000012	0.32	Uterus
ENSG00000107902.13	LHPP	chr10_124616865_T_C_b38	rs4962671	0.000024	-0.21	Brain - Putamen (basal ganglia)
ENSG00000107902.13	LHPP	chr10_124616865_T_C_b38	rs4962671	0.00007	-0.11	Artery - Tibial
ENSG00000189319.13	FAM53B	chr10_124616865_T_C_b38	rs4962671	0.000088	-0.12	Cells - Cultured fibroblasts

Gencode Id	Gene Symbol	Variant Id	SNP Id	P-Value	NES	Tissue
ENSG00000170430.9	MGMT	chr10_129632422_G_A_b38	rs524804	2.10E-70	-0.64	Thyroid
ENSG00000170430.9	MGMT	chr10_129632422_G_A_b38	rs524804	8.80E-64	-0.63	Skin - Sun Exposed (Lower leg)
ENSG00000170430.9	MGMT	chr10_129632422_G_A_b38	rs524804	5.10E-62	-0.73	Esophagus - Mucosa
ENSG00000170430.9	MGMT	chr10_129632422_G_A_b38	rs524804	7.40E-61	-0.64	Adipose - Visceral (Omentum)
ENSG00000170430.9	MGMT	chr10_129632422_G_A_b38	rs524804	2.50E-60	-0.63	Adipose - Subcutaneous
ENSG00000170430.9	MGMT	chr10_129632422_G_A_b38	rs524804	7.30E-60	-0.62	Skin - Not Sun Exposed (Suprapubic)
ENSG00000170430.9	MGMT	chr10_129632422_G_A_b38	rs524804	1.00E-55	-0.52	Artery - Tibial
ENSG00000170430.9	MGMT	chr10_129632422_G_A_b38	rs524804	2.10E-54	-0.51	Cells - Cultured fibroblasts
ENSG00000170430.9	MGMT	chr10_129632422_G_A_b38	rs524804	2.50E-50	-0.62	Lung
ENSG00000170430.9	MGMT	chr10_129632422_G_A_b38	rs524804	4.90E-48	-0.37	Whole Blood
ENSG00000170430.9	MGMT	chr10_129632422_G_A_b38	rs524804	8.10E-47	-0.71	Artery - Aorta
ENSG00000170430.9	MGMT	chr10_129632422_G_A_b38	rs524804	1.80E-46	-0.41	Muscle - Skeletal
ENSG00000170430.9	MGMT	chr10_129632422_G_A_b38	rs524804	1.80E-43	-0.54	Nerve - Tibial
ENSG00000170430.9	MGMT	chr10_129632422_G_A_b38	rs524804	2.70E-41	-0.56	Breast - Mammary Tissue
ENSG00000170430.9	MGMT	chr10_129632422_G_A_b38	rs524804	4.30E-41	-0.56	Esophagus - Muscularis
ENSG00000170430.9	MGMT	chr10_129632422_G_A_b38	rs524804	1.20E-38	-0.53	Heart - Atrial Appendage
ENSG00000170430.9	MGMT	chr10_129632422_G_A_b38	rs524804	6.10E-38	-0.68	Colon - Transverse
ENSG00000170430.9	MGMT	chr10_129632422_G_A_b38	rs524804	8.10E-35	-0.65	Stomach
ENSG00000170430.9	MGMT	chr10_129632422_G_A_b38	rs524804	3.10E-34	-0.63	Esophagus - Gastroesophageal Junction
ENSG00000170430.9	MGMT	chr10_129632422_G_A_b38	rs524804	2.50E-33	-0.57	Heart - Left Ventricle
ENSG00000170430.9	MGMT	chr10_129632422_G_A_b38	rs524804	1.80E-30	-0.54	Colon - Sigmoid
ENSG00000170430.9	MGMT	chr10_129632422_G_A_b38	rs524804	2.00E-29	-0.73	Adrenal Gland
ENSG00000170430.9	MGMT	chr10_129632422_G_A_b38	rs524804	2.50E-28	-0.54	Testis
ENSG00000170430.9	MGMT	chr10_129632422_G_A_b38	rs524804	2.60E-27	-0.48	Liver
ENSG00000170430.9	MGMT	chr10_129632422_G_A_b38	rs524804	1.30E-26	-0.65	Prostate
ENSG00000170430.9	MGMT	chr10_129632422_G_A_b38	rs524804	2.60E-23	-0.67	Artery - Coronary
ENSG00000170430.9	MGMT	chr10_129632422_G_A_b38	rs524804	3.00E-23	-0.63	Pituitary
ENSG00000170430.9	MGMT	chr10_129632422_G_A_b38	rs524804	6.90E-22	-0.59	Pancreas
ENSG00000170430.9	MGMT	chr10_129632422_G_A_b38	rs524804	5.50E-19	-0.49	Brain - Caudate (basal ganglia)
ENSG00000170430.9	MGMT	chr10_129632422_G_A_b38	rs524804	3.60E-18	-0.59	Brain - Cortex
ENSG00000170430.9	MGMT	chr10_129632422_G_A_b38	rs524804	1.60E-17	-0.59	Brain - Cerebellar Hemisphere
ENSG00000170430.9	MGMT	chr10_129632422_G_A_b38	rs524804	4.50E-17	-0.58	Brain - Cerebellum
ENSG00000170430.9	MGMT	chr10_129632422_G_A_b38	rs524804	2.20E-16	-0.46	Brain - Nucleus accumbens (basal [illegible]
ENSG00000170430.9	MGMT	chr10_129632422_G_A_b38	rs524804	2.50E-16	-0.53	Brain - Spinal cord (cervical c-1)
ENSG00000170430.9	MGMT	chr10_129632422_G_A_b38	rs524804	3.40E-16	-0.6	Spleen
ENSG00000170430.9	MGMT	chr10_129632422_G_A_b38	rs524804	2.00E-14	-0.64	Small Intestine - Terminal Ileum
ENSG00000170430.9	MGMT	chr10_129632422_G_A_b38	rs524804	3.10E-14	-0.82	Cells - EBV-transformed lymphocytes
ENSG00000170430.9	MGMT	chr10_129632422_G_A_b38	rs524804	4.70E-14	-0.49	Brain - Hypothalamus
ENSG00000170430.9	MGMT	chr10_129632422_G_A_b38	rs524804	1.70E-13	-0.52	Brain - Anterior cingulate cortex (BA24)
ENSG00000170430.9	MGMT	chr10_129632422_G_A_b38	rs524804	9.90E-13	-0.57	Brain - Hippocampus
ENSG00000170430.9	MGMT	chr10_129632422_G_A_b38	rs524804	1.30E-12	-0.47	Brain - Frontal Cortex (BA9)
ENSG00000170430.9	MGMT	chr10_129632422_G_A_b38	rs524804	1.90E-11	-0.44	Brain - Putamen (basal ganglia)
ENSG00000170430.9	MGMT	chr10_129632422_G_A_b38	rs524804	3.70E-11	-0.6	Minor Salivary Gland
ENSG00000170430.9	MGMT	chr10_129632422_G_A_b38	rs524804	5.70E-11	-0.57	Brain - Substantia nigra
ENSG00000170430.9	MGMT	chr10_129632422_G_A_b38	rs524804	3.90E-09	-0.63	Uterus
ENSG00000170430.9	MGMT	chr10_129632422_G_A_b38	rs524804	4.60E-09	-0.45	Brain - Amygdala
ENSG00000170430.9	MGMT	chr10_129632422_G_A_b38	rs524804	5.30E-08	-0.58	Vagina
ENSG00000170430.9	MGMT	chr10_129632422_G_A_b38	rs524804	6.90E-08	-0.47	Ovary
ENSG00000108001.13	EBF3	chr10_129632422_G_A_b38	rs524804	0.000015	-0.14	Esophagus - Mucosa
ENSG00000172725.13	CORO1B	chr11_67053385_C_A_b38	rs117773425	0.000049	0.42	Spleen
ENSG00000174165.7	ZDHHC24	chr11_67053385_C_A_b38	rs117773425	0.000052	0.51	Artery - Tibial
ENSG00000255468.6	RP11-867G23.8	chr11_67053385_C_A_b38	rs117773425	0.00033	-0.66	Thyroid
ENSG00000105287.12	PRKD2	chr19_46778988_C_T_b38	rs56385874	1.10E-19	0.27	Artery - Tibial
ENSG00000181027.10	FKRP	chr19_46778988_C_T_b38	rs56385874	2.50E-19	0.38	Skin - Sun Exposed (Lower leg)
ENSG00000181027.10	FKRP	chr19_46778988_C_T_b38	rs56385874	4.20E-17	0.3	Muscle - Skeletal
ENSG00000181027.10	FKRP	chr19_46778988_C_T_b38	rs56385874	1.40E-16	0.39	Skin - Not Sun Exposed (Suprapubic)

Gencode Id	Gene Symbol	Variant Id	SNP Id	P-Value	NES	Tissue
ENSG00000181027.10	FKRP	chr19_46778988_C_T_b38	rs56385874	2.10E-16	0.54	Esophagus - Muscularis
ENSG00000181027.10	FKRP	chr19_46778988_C_T_b38	rs56385874	7.70E-16	0.4	Nerve - Tibial
ENSG00000105281.12	SLC1A5	chr19_46778988_C_T_b38	rs56385874	1.10E-13	0.25	Cells - Cultured fibroblasts
ENSG00000105287.12	PRKD2	chr19_46778988_C_T_b38	rs56385874	3.30E-13	0.16	Whole Blood
ENSG00000105287.12	PRKD2	chr19_46778988_C_T_b38	rs56385874	4.60E-13	0.32	Cells - Cultured fibroblasts
ENSG00000181027.10	FKRP	chr19_46778988_C_T_b38	rs56385874	6.40E-13	0.34	Artery - Tibial
ENSG00000181027.10	FKRP	chr19_46778988_C_T_b38	rs56385874	1.90E-12	0.35	Thyroid
ENSG00000105287.12	PRKD2	chr19_46778988_C_T_b38	rs56385874	1.10E-11	0.31	Nerve - Tibial
ENSG00000181027.10	FKRP	chr19_46778988_C_T_b38	rs56385874	1.30E-11	0.35	Adipose - Subcutaneous
ENSG00000181027.10	FKRP	chr19_46778988_C_T_b38	rs56385874	2.90E-10	0.29	Adipose - Visceral (Omentum)
ENSG00000181027.10	FKRP	chr19_46778988_C_T_b38	rs56385874	3.20E-10	0.48	Colon - Sigmoid
ENSG00000181027.10	FKRP	chr19_46778988_C_T_b38	rs56385874	4.90E-10	0.28	Cells - Cultured fibroblasts
ENSG00000181027.10	FKRP	chr19_46778988_C_T_b38	rs56385874	5.20E-10	0.31	Esophagus - Mucosa
ENSG00000105287.12	PRKD2	chr19_46778988_C_T_b38	rs56385874	6.30E-10	0.23	Thyroid
ENSG00000090372.14	STRN4	chr19_46778988_C_T_b38	rs56385874	2.40E-09	0.14	Skin - Sun Exposed (Lower leg)
ENSG00000181027.10	FKRP	chr19_46778988_C_T_b38	rs56385874	7.20E-09	0.29	Colon - Transverse
ENSG00000181027.10	FKRP	chr19_46778988_C_T_b38	rs56385874	3.70E-08	0.36	Breast - Mammary Tissue
ENSG00000105287.12	PRKD2	chr19_46778988_C_T_b38	rs56385874	6.30E-08	0.22	Esophagus - Muscularis
ENSG00000181027.10	FKRP	chr19_46778988_C_T_b38	rs56385874	9.20E-08	0.31	Artery - Aorta
ENSG00000181027.10	FKRP	chr19_46778988_C_T_b38	rs56385874	1.30E-07	0.23	Lung
ENSG00000181027.10	FKRP	chr19_46778988_C_T_b38	rs56385874	3.20E-07	0.33	Brain - Hippocampus
ENSG00000090372.14	STRN4	chr19_46778988_C_T_b38	rs56385874	3.70E-07	0.13	Esophagus - Mucosa
ENSG00000181027.10	FKRP	chr19_46778988_C_T_b38	rs56385874	4.80E-07	0.3	Heart - Left Ventricle
ENSG00000090372.14	STRN4	chr19_46778988_C_T_b38	rs56385874	5.20E-07	0.15	Skin - Not Sun Exposed (Suprapubic)
ENSG00000181027.10	FKRP	chr19_46778988_C_T_b38	rs56385874	6.00E-07	0.46	Prostate
ENSG00000105287.12	PRKD2	chr19_46778988_C_T_b38	rs56385874	0.0000033	0.27	Pancreas
ENSG00000105287.12	PRKD2	chr19_46778988_C_T_b38	rs56385874	0.0000048	0.25	Colon - Sigmoid
ENSG00000105287.12	PRKD2	chr19_46778988_C_T_b38	rs56385874	0.0000049	0.17	Adipose - Visceral (Omentum)
ENSG00000105287.12	PRKD2	chr19_46778988_C_T_b38	rs56385874	0.0000086	0.28	Pituitary
ENSG00000181027.10	FKRP	chr19_46778988_C_T_b38	rs56385874	0.0000095	0.15	Whole Blood
ENSG00000105287.12	PRKD2	chr19_46778988_C_T_b38	rs56385874	0.000014	0.34	Liver
ENSG00000105287.12	PRKD2	chr19_46778988_C_T_b38	rs56385874	0.000021	0.18	Breast - Mammary Tissue
ENSG00000181027.10	FKRP	chr19_46778988_C_T_b38	rs56385874	0.000029	0.38	Esophagus - Gastroesophageal Junction
ENSG00000269487.1	CTB-174O21.2	chr19_46778988_C_T_b38	rs56385874	0.000031	-0.31	Nerve - Tibial
ENSG00000105281.12	SLC1A5	chr19_46778988_C_T_b38	rs56385874	0.000036	0.19	Artery - Aorta
ENSG00000105281.12	SLC1A5	chr19_46778988_C_T_b38	rs56385874	0.000039	0.14	Artery - Tibial
ENSG00000090372.14	STRN4	chr19_46778988_C_T_b38	rs56385874	0.000045	0.091	Whole Blood
ENSG00000181027.10	FKRP	chr19_46778988_C_T_b38	rs56385874	0.000057	0.33	Brain - Cerebellar Hemisphere
ENSG00000105287.12	PRKD2	chr19_46778988_C_T_b38	rs56385874	0.000066	0.15	Muscle - Skeletal
ENSG00000090372.14	STRN4	chr19_46778988_C_T_b38	rs56385874	0.000089	0.11	Cells - Cultured fibroblasts
ENSG00000101144.12	BMP7	chr20_57248706_G_A_b38	rs6127980	4.10E-10	0.17	Skin - Sun Exposed (Lower leg)
ENSG00000101146.12	RAE1	chr20_57248706_G_A_b38	rs6127980	0.0000036	0.15	Testis
ENSG00000101144.12	BMP7	chr20_57248706_G_A_b38	rs6127980	0.000015	0.18	Skin - Not Sun Exposed (Suprapubic)
ENSG00000132819.16	RBM38	chr20_57248706_G_A_b38	rs6127980	0.00002	0.22	Esophagus - Mucosa

Table S13: Association of rs7133378 & rs1716407 and rs56361048 & rs7258937 with BFPAdj in males when both SNPs were in the model

SNP	CHR	BP (HG19)	A0	A1	β	SE	P	Interaction P
**rs7133378 & rs1716407 both in the model**								
rs7133378	12	124,409,502	A	G	-0.15	0.02	2.53E-12	0.72
rs1716407	12	124,515,218	A	G	0.11	0.02	4.20E-8	
**rs56361048 & rs7258937 both in the model**								
rs56361048	19	33,885,318	C	T	-0.10	0.03	2.66E-4	0.95
rs7258937	19	33,938,800	T	C	-0.14	0.02	5.09E-12	

Linear regression was used to test association of the two SNPs with BFP in males when Age, Age^2, Albumin, $Albumin^2$, SHBG, $SHBG^2$, Testosterone, $Testosterone^2$ and first 10 PCs were in the model.

Table S14: Association of rs74841570 and rs11057402 with BFPAdj in females when both SNPs were in the model

SNP	CHR	BP (HG19)	A0	A1	β	SE	P	Int β	Int SE	Int P
rs74841570	12	124,407,903	C	A	-0.25	0.05	3.33E-7	0.39	0.15	1.20E-2
rs11057402	12	124,430,767	A	T	0.22	0.03	5.42E-11			

Linear regression was used to test association of the two SNPs with BFP in females when Age, Age^2, Albumin, $Albumin^2$, SHBG, $SHBG^2$, Testosterone, $Testosterone^2$ and first 10 PCs were in the model.

Supplementary Figures

Figure S1: Distribution of BFP in males and females

A.

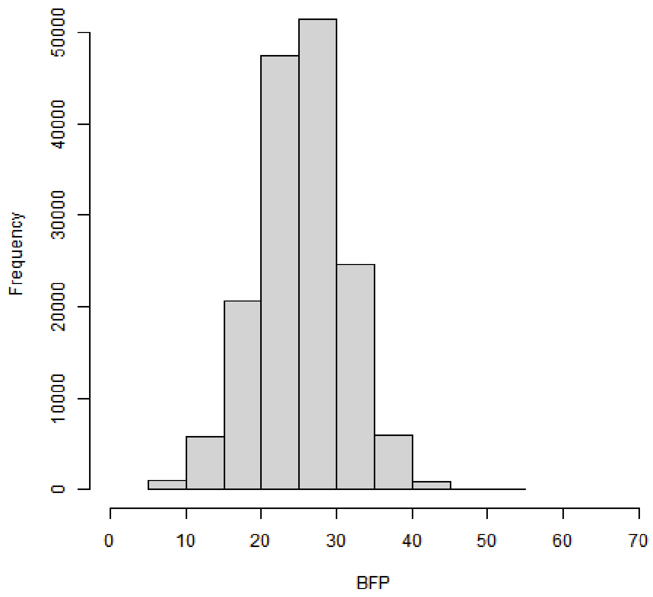

B.

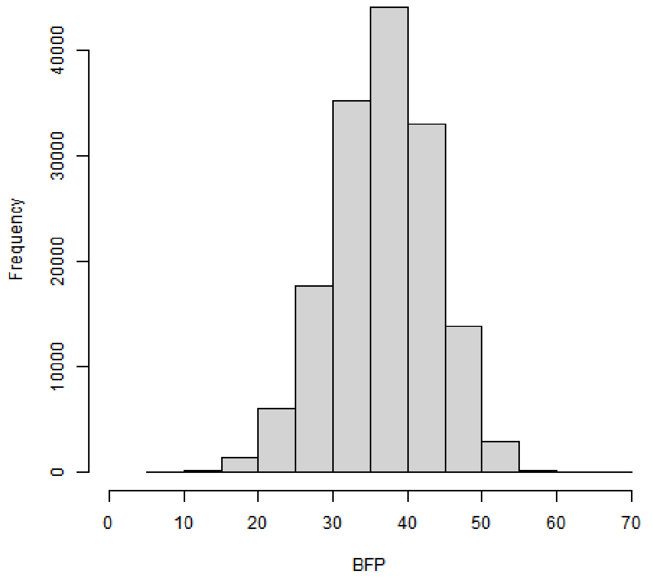

A: Males, B: Females

The plot shows the distribution of BFP in 157,937 males (A) and 154,337 females (B) from the UK biobank.

Figure S2: Association of BFPAdj GWAS SNPs in the current analysis with SHBG in Ruth et al analysis (1) in males (N = 193) and females (N = 174)

A.

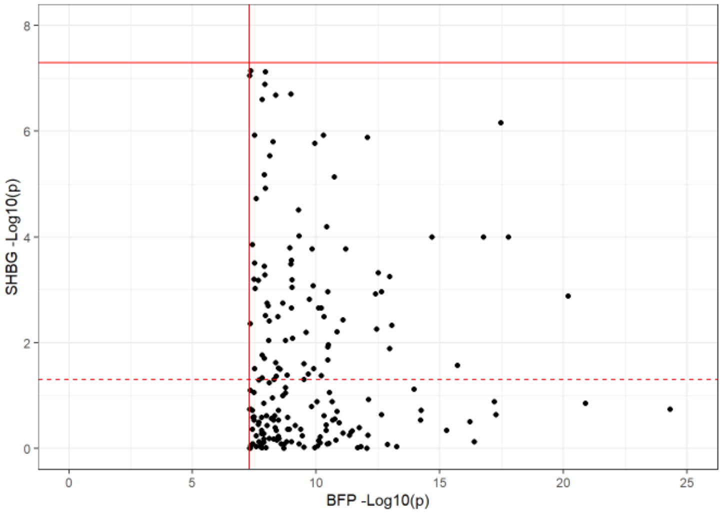

B.

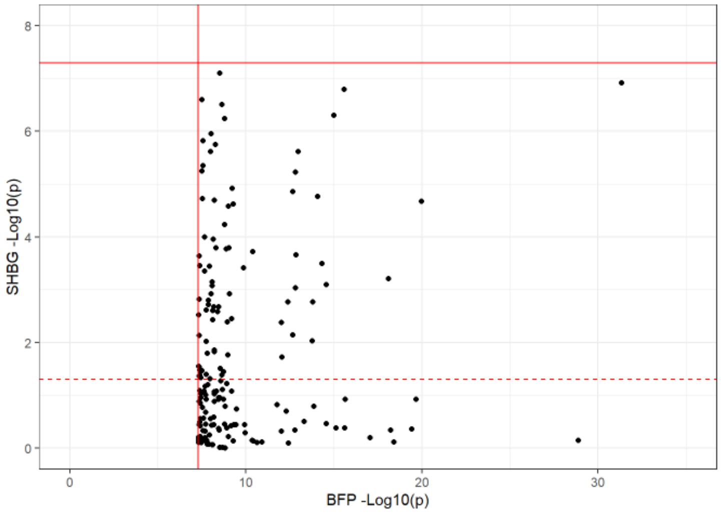

A: Males, B: Females

The plots show -$\log_{10}$(p-value) regarding association of SNPs with BFPAdj vs. their association with SHBG in males (A) and females (B).

The solid red line shows x = 7.3 and y = 7.3 (genome-wide significance). The dashed red line shows y = 1.3 (nominal significance).

Figure S3: Association of BFPAdj GWAS SNPs in the current analysis with testosterone in Ruth et al analysis (1) in males and females

A.

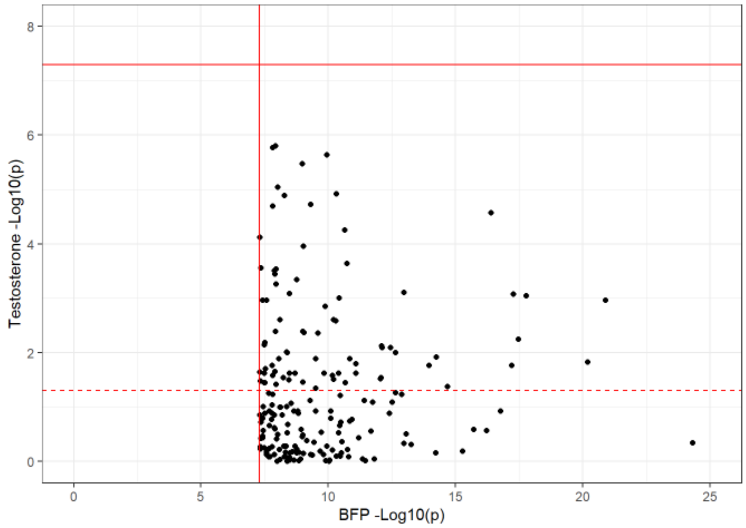

B.

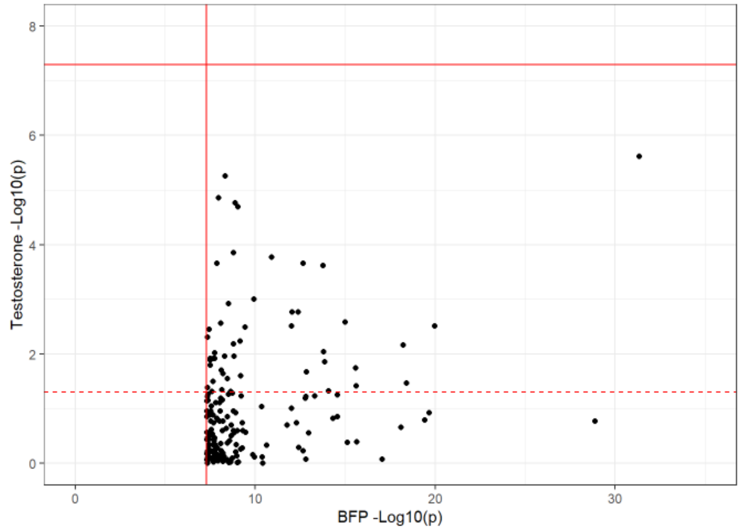

A: Males, B: Female

The plots show -$\log_{10}$(p-value) regarding association of SNPs with BFPAdj vs. their association with testosterone in males (A) and females (B).

The solid red line shows x = 7.3 and y = 7.3 (genome-wide significance). The dashed red line shows y = 1.3 (nominal significance).

Figure S4: The effect sizes of independent BFPAdj GWAS loci in males (N = 193) and females (N = 174)

A.

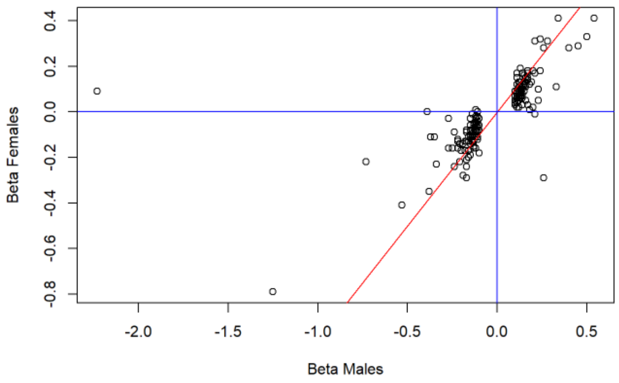

B.

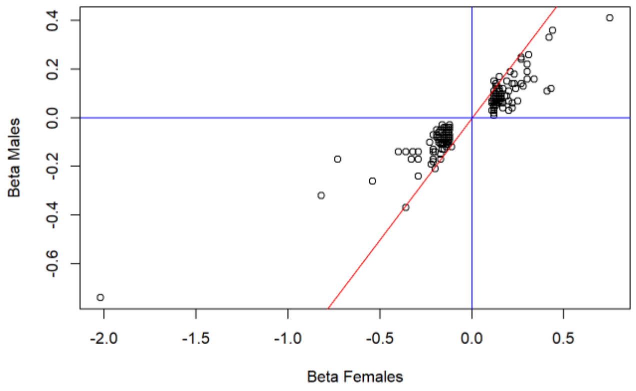

A: Males; the plot shows the effect (β ± SE) of BFPAdj male GWAS loci on BFPAdj in males (X axis) vs. their effect (β ± SE) on BFPAdj in females (Y axis).

B: Female; the plot shows the effect (β ± SE) of BFPAdj female GWAS loci on BFPAdj in females (X axis) vs. their effect on BFPAdj in males (Y axis).

The red line is x = y, and blue lines are x = 0 and y = 0.

Figure S5: The p-values (-$\log_{10}$) of independent BFPAdj GWAS loci in males (N = 193) and females (N = 174)

A.

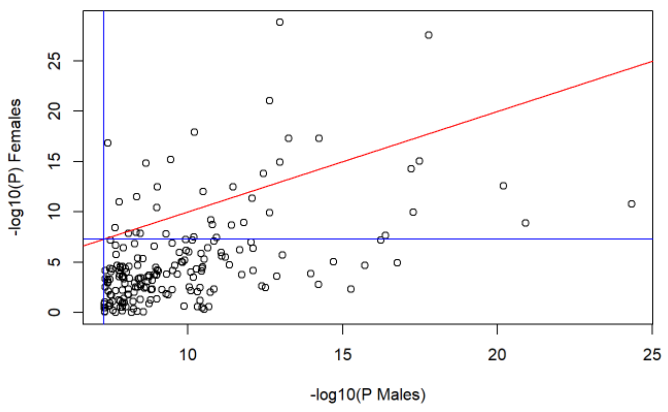

B.

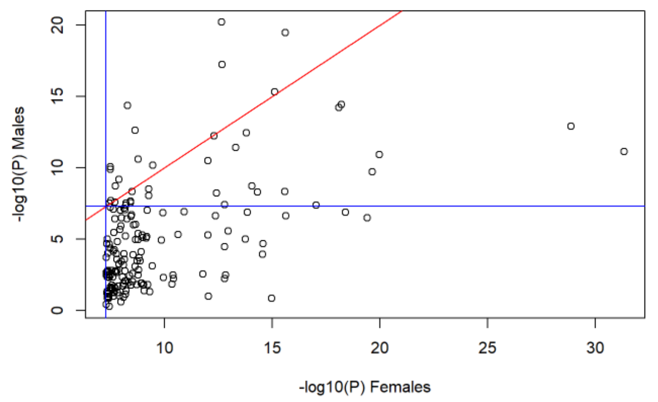

A: Males; the plot show -$\log_{10}$(p-value) regarding association of BFPAdj male GWAS loci with BFPAdj in males (X axis) vs. their association with BFPAdj in females (Y axis).

B: Females; the plot show -$\log_{10}$(p-value) regarding association of BFPAdj female GWAS loci with BFPAdj in females (X axis) vs. their association with BFPAdj in males (Y axis).

The red line is x = y, and blue lines are x = 7.3 and y = 7.3 (genome-wide significance).

Figure S6: Association of BFPAdj GWAS SNPs in the current analysis with BFP in Neale's round 2 GWAS results

A.

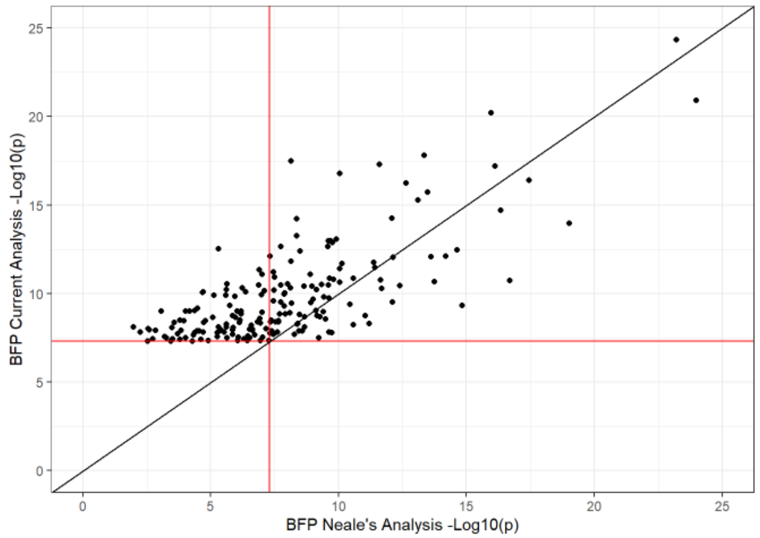

B.

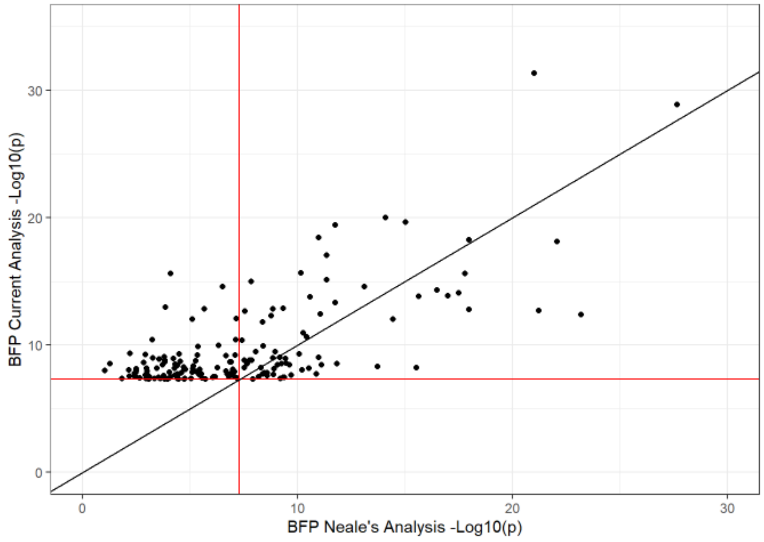

A: Males, B: Female

The plots show -log_{10}(p-value) regarding association of BFPAdj GWAS loci with BFP in Neale's round 2 GWAS results (X axis) vs. BFPAdj in the current analysis (Y axis) in males (A) and females (B).

The solid red line shows x = 7.3 and y = 7.3 (genome-wide significance). The black line shows x = y.

Figure S7: Association of SNPs on Chr X with BFPAdj in males and females

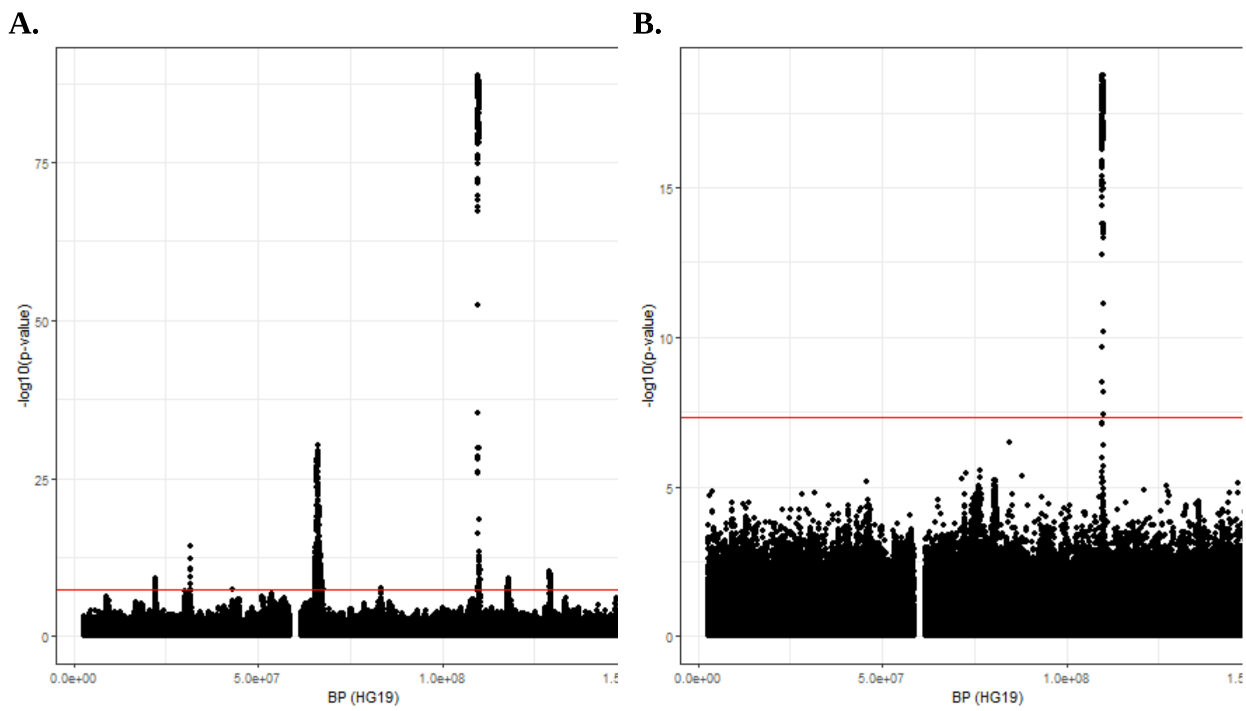

A: Males, B: Females

Associations of SNPs on chromosome X were tested with BFPAdj in 157,937 males and 154,337 females from the UK biobank separately. Age, centered age^2, serum albumin, centered albumin^2, serum SHBG, centered SHBG^2, serum testosterone, centered testosterone^2, and first ten genetic PCs were included as covariates in the model. The X axis shows the SNP location on chromosome X and the Y axis shows -$\log_{10}$ (p-value) regarding SNP association with BFPAdj in males (A) and females (B).

References

1. Ruth KS, Day FR, Tyrrell J, Thompson DJ, Wood AR, Mahajan A, et al. Using human genetics to understand the disease impacts of testosterone in men and women. Nat Med. 2020;26(2):252-8.
2. Graham SE, Clarke SL, Wu KH, Kanoni S, Zajac GJM, Ramdas S, et al. The power of genetic diversity in genome-wide association studies of lipids. Nature. 2021;600(7890):675-9.
3. Mahajan A, Spracklen CN, Zhang W, Ng MCY, Petty LE, Kitajima H, et al. Multi-ancestry genetic study of type 2 diabetes highlights the power of diverse populations for discovery and translation. Nat Genet. 2022;54(5):560-72.
4. Aragam KG, Jiang T, Goel A, Kanoni S, Wolford BN, Atri DS, et al. Discovery and systematic characterization of risk variants and genes for coronary artery disease in over a million participants. Nat Genet. 2022;54(12):1803-15.
5. Agrawal S, Wang M, Klarqvist MDR, Smith K, Shin J, Dashti H, et al. Inherited basis of visceral, abdominal subcutaneous and gluteofemoral fat depots. Nat Commun. 2022;13(1):3771.
6. Pulit SL, Stoneman C, Morris AP, Wood AR, Glastonbury CA, Tyrrell J, et al. Meta-analysis of genome-wide association studies for body fat distribution in 694 649 individuals of European ancestry. Hum Mol Genet. 2019;28(1):166-74.
7. Lu Y, Day FR, Gustafsson S, Buchkovich ML, Na J, Bataille V, et al. New loci for body fat percentage reveal link between adiposity and cardiometabolic disease risk. Nat Commun. 2016;7:10495.

MR Analysis in Males

1- T2D

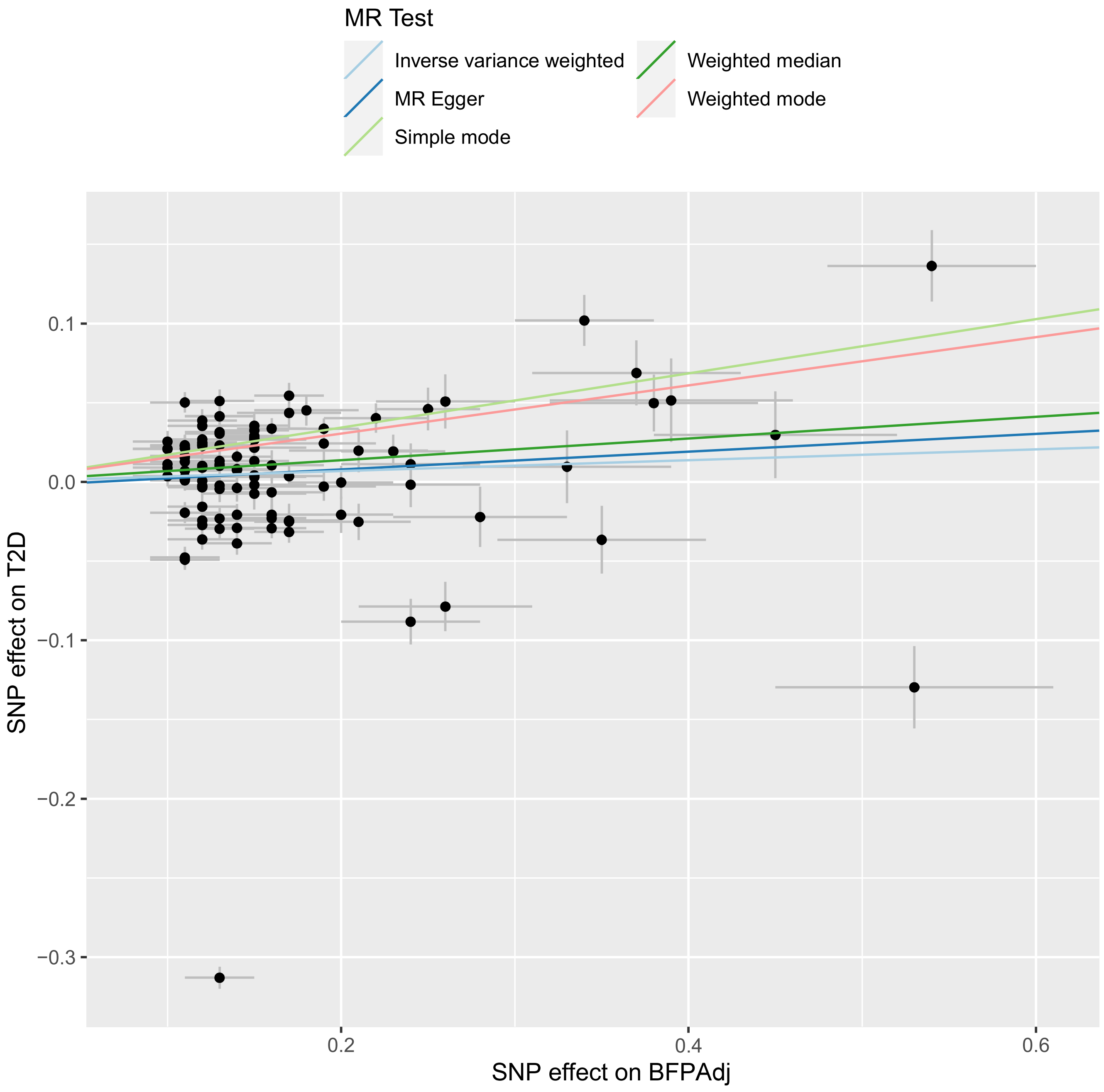
MR Test
Inverse variance weighted
MR Egger
Simple mode
Weighted median
Weighted mode
0.1
0.0
−0.1
−0.2
−0.3
SNP effect on T2D
0.2
0.4
0.6
SNP effect on BFPAdj

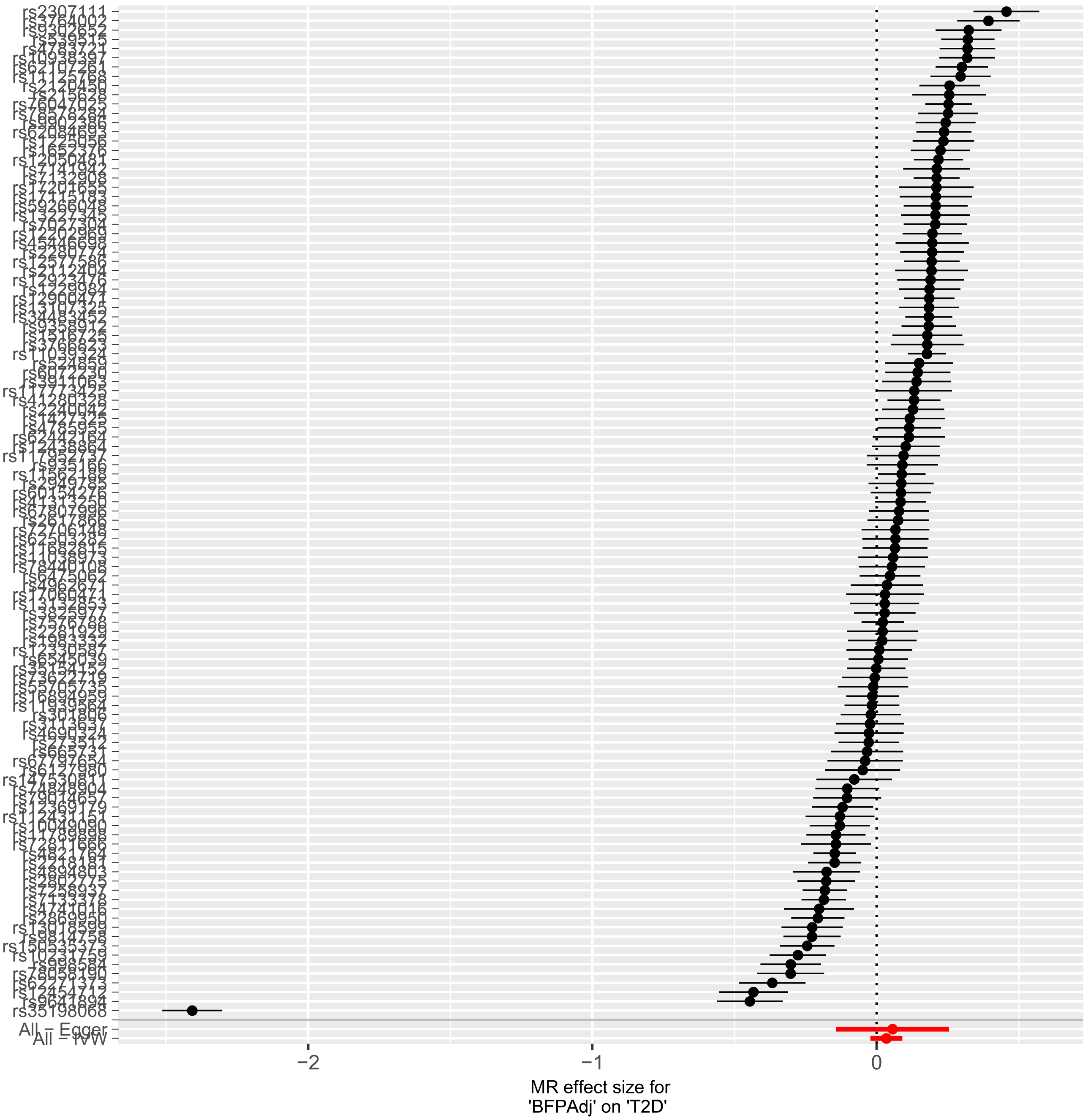
rs2307111
rs35198068
All - Egger
All - IVW
−2
−1
0
MR effect size for
'BFPAdj' on 'T2D'

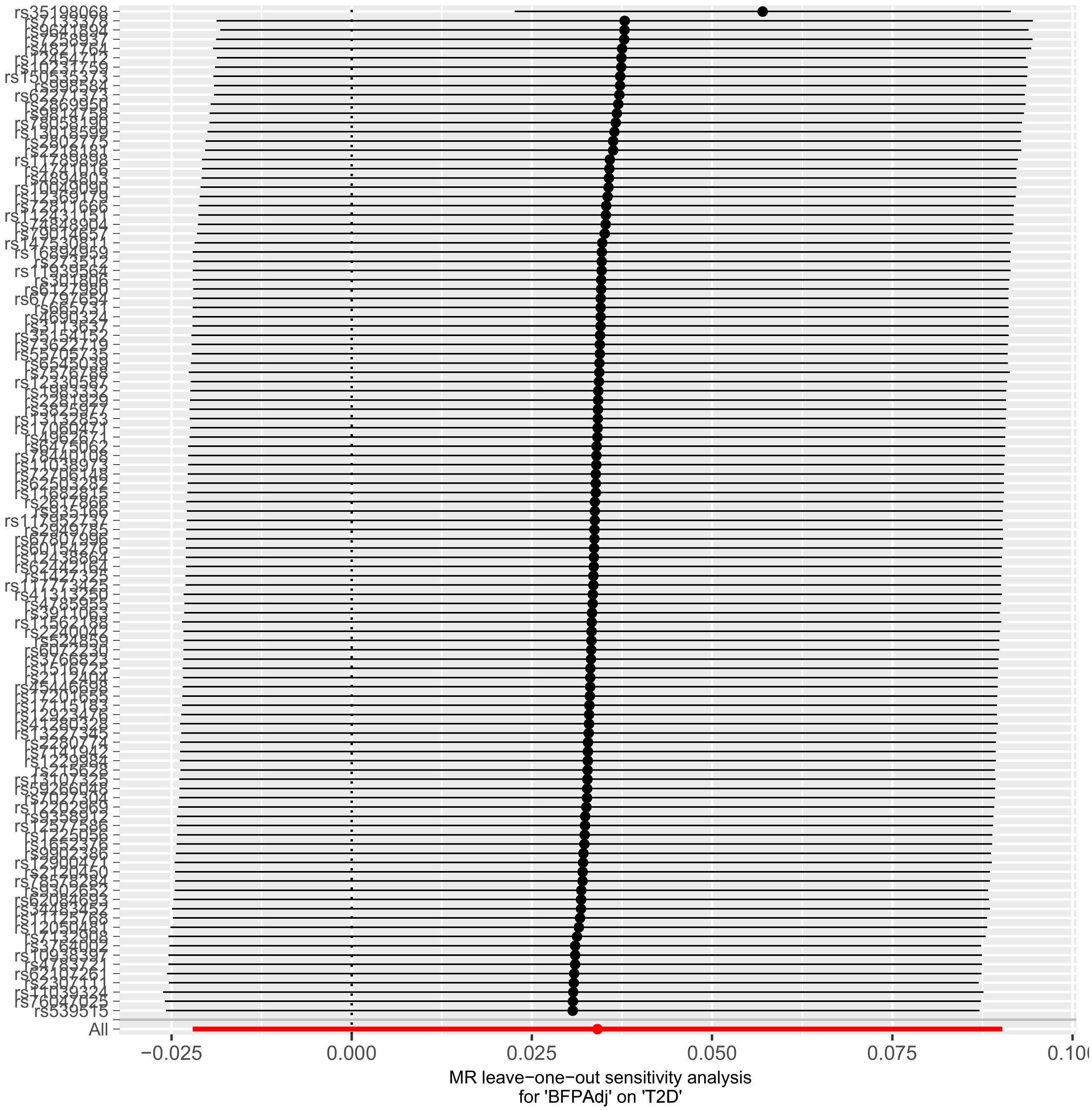

rs35198068
rs539515
All
−0.025
0.000
0.025
0.050
0.075
MR leave−one−out sensitivity analysis
for 'BFPAdj' on 'T2D'

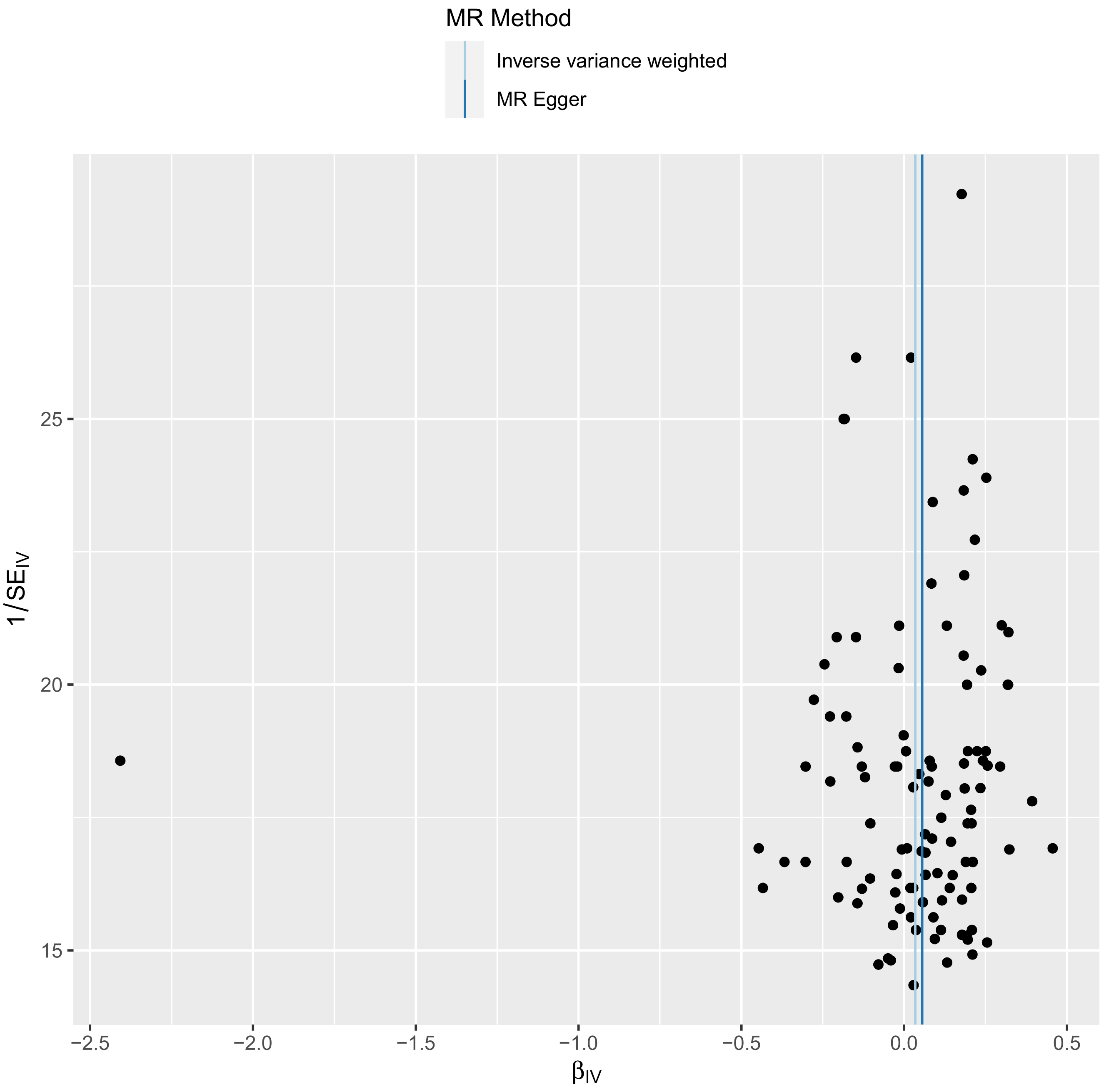

MR Method
Inverse variance weighted
MR Egger
1/SE_IV
25
20
15
−2.5
−2.0
−1.5
−1.0
−0.5
0.0
0.5
β_IV

2- CAD

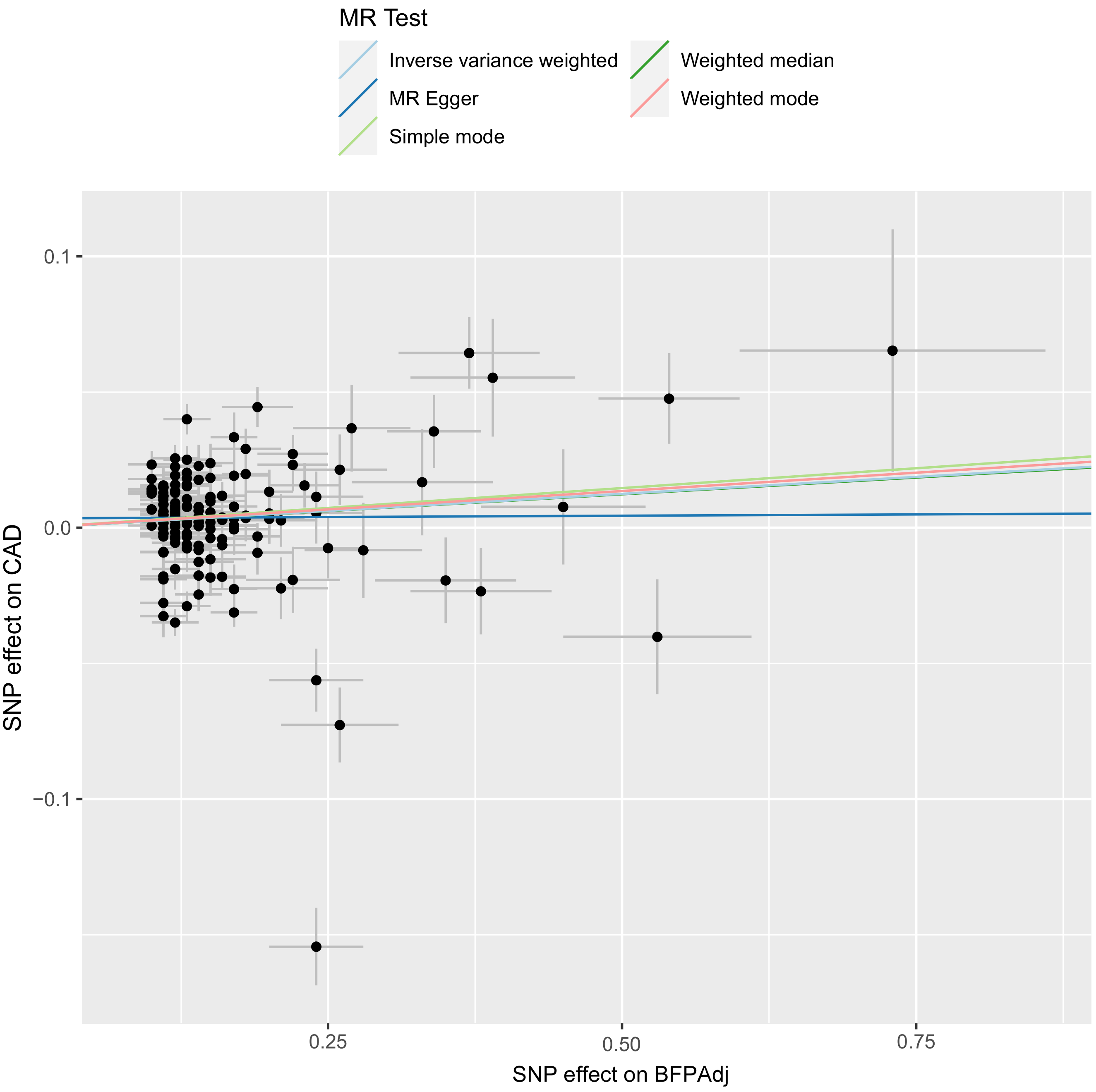
MR Test
Inverse variance weighted
MR Egger
Simple mode
Weighted median
Weighted mode
0.1
0.0
−0.1
0.25
0.50
0.75
SNP effect on CAD
SNP effect on BFPAdj

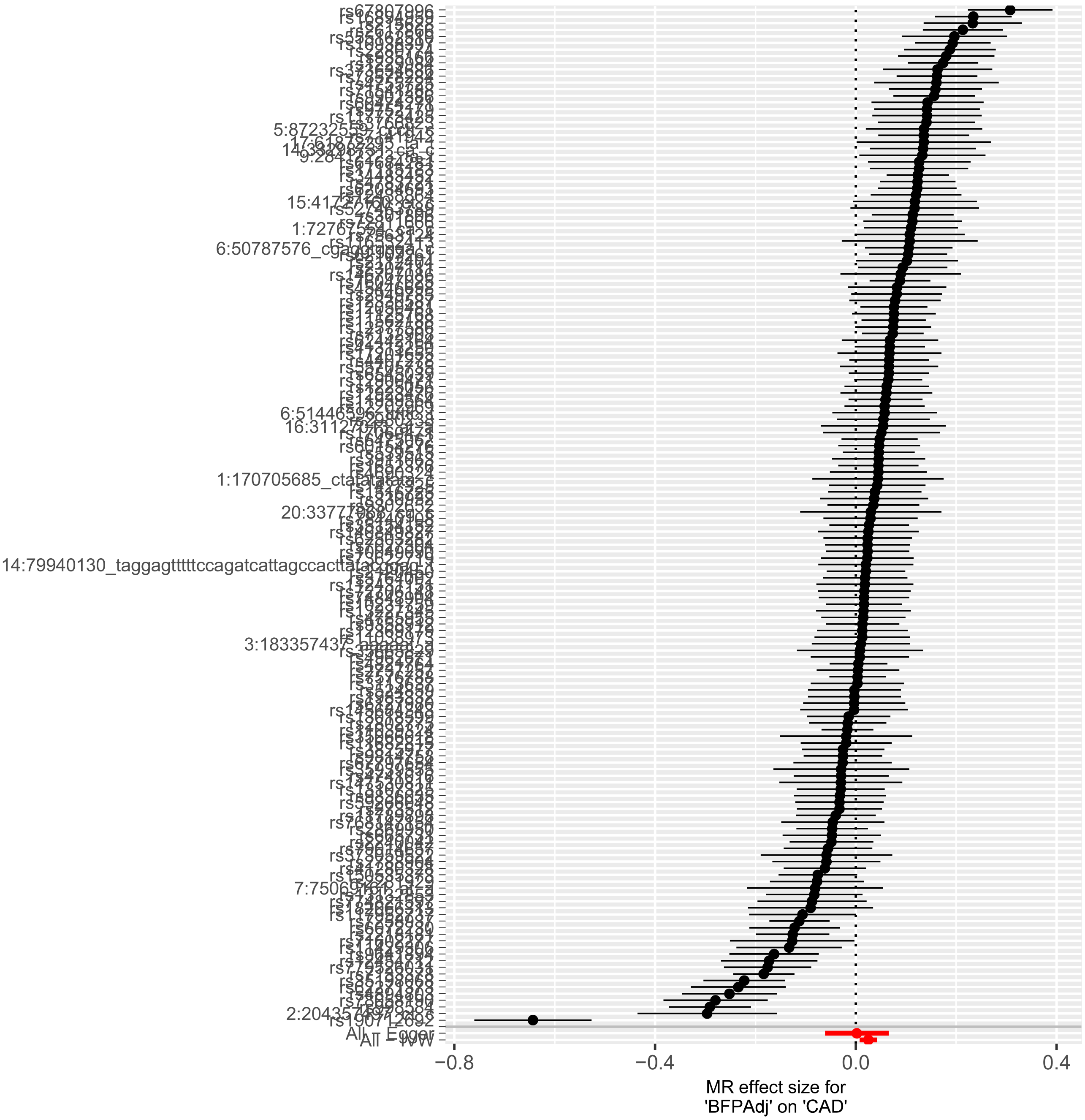
−0.8
−0.4
0.0
0.4
MR effect size for
'BFPAdj' on 'CAD'

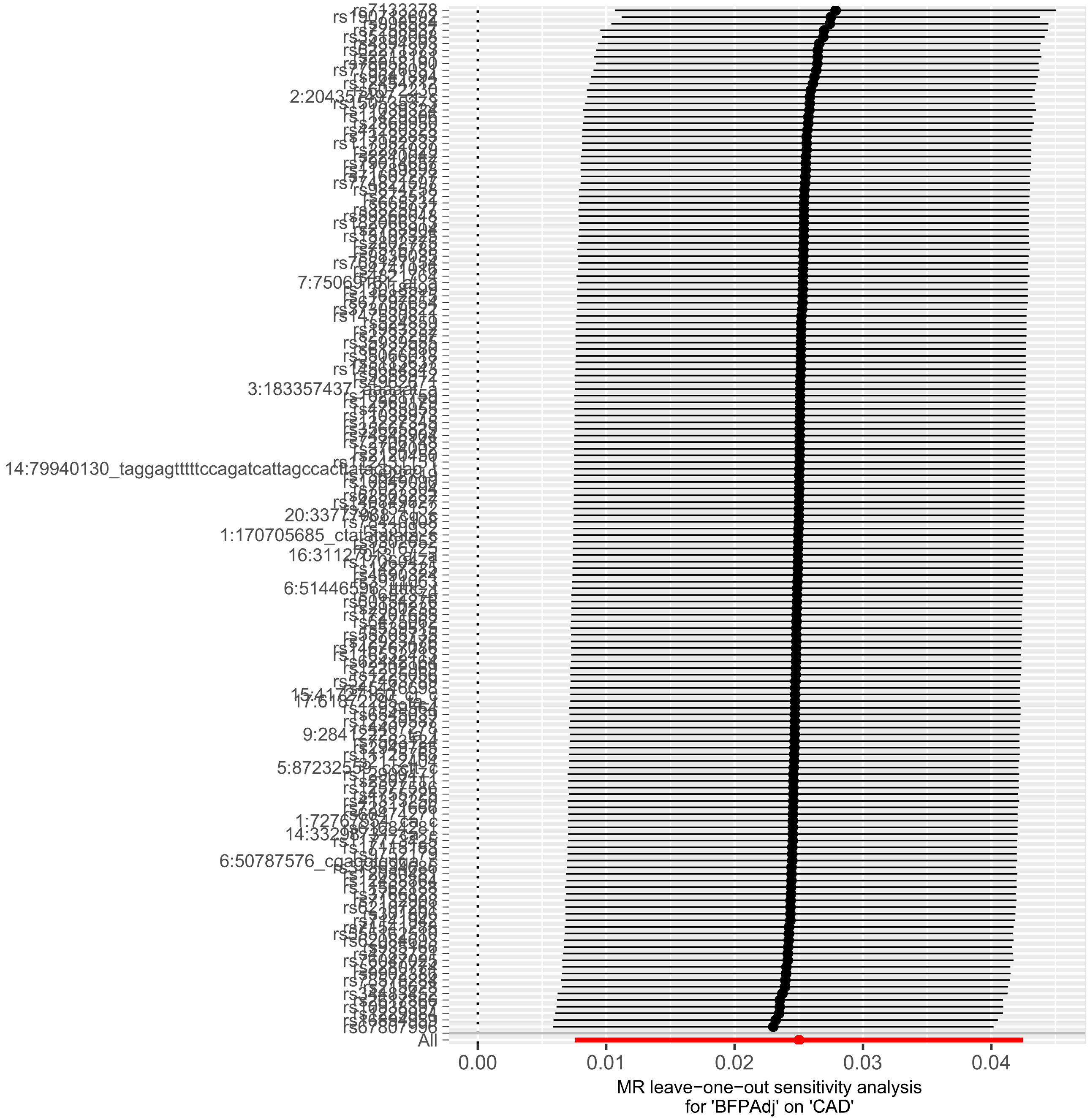
All
0.00
0.01
0.02
0.03
0.04
MR leave−one−out sensitivity analysis
for 'BFPAdj' on 'CAD'

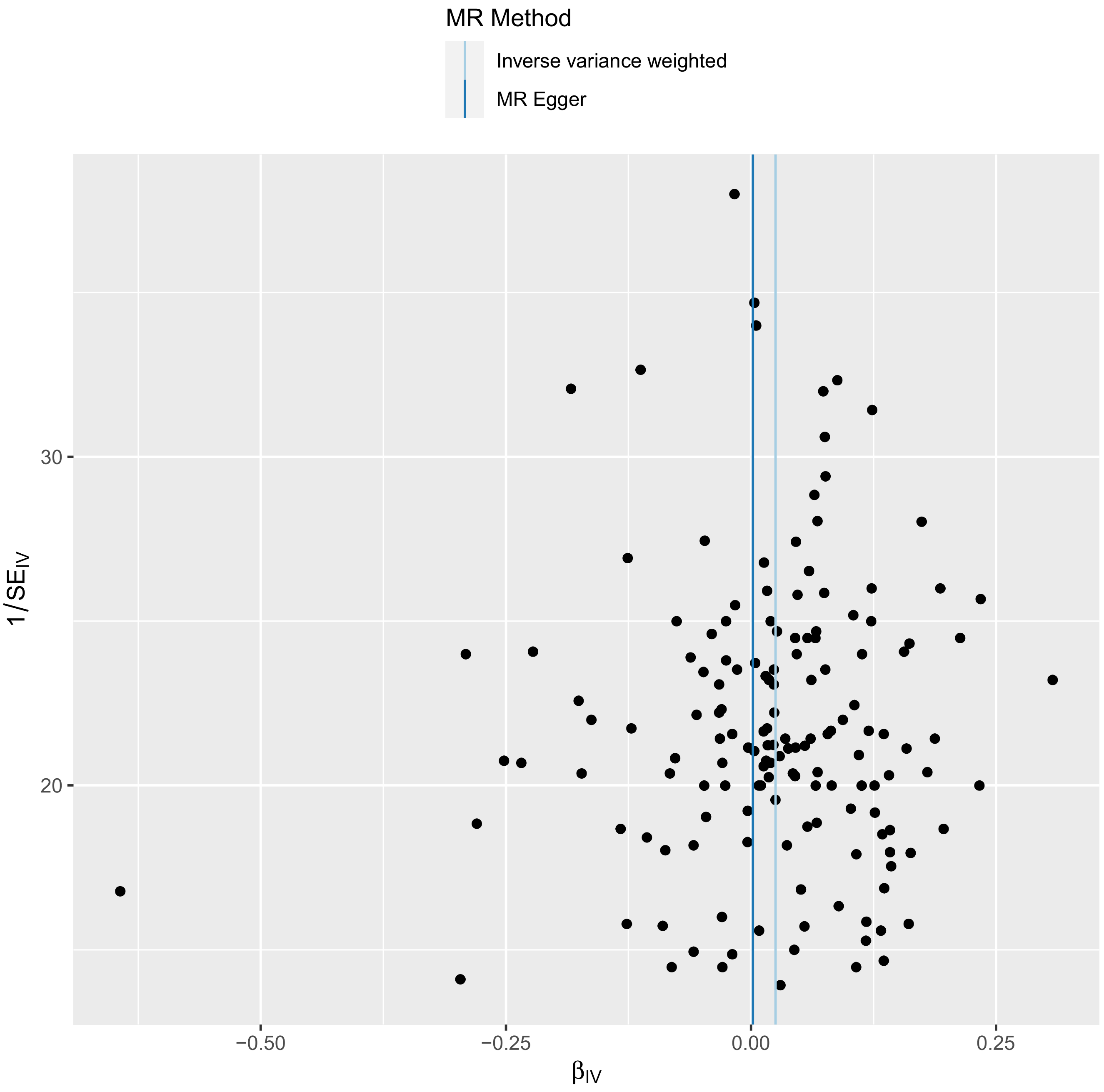
MR Method
Inverse variance weighted
MR Egger
1/SE$_{IV}$
30
20
−0.50
−0.25
0.00
0.25
β_{IV}

3- HDL

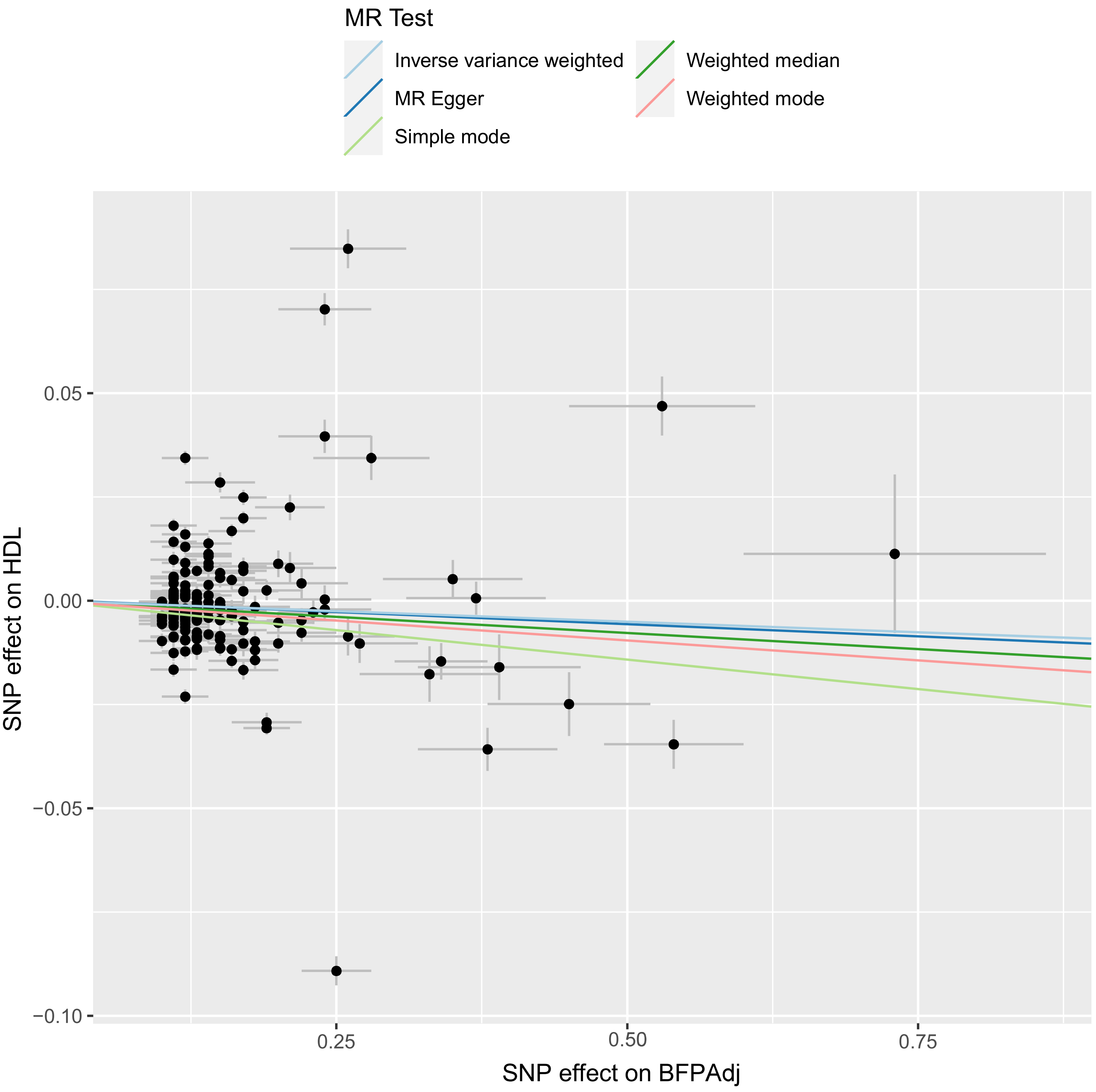
MR Test
Inverse variance weighted
MR Egger
Simple mode
Weighted median
Weighted mode
0.05
0.00
−0.05
−0.10
SNP effect on HDL
0.25
0.50
0.75
SNP effect on BFPAdj

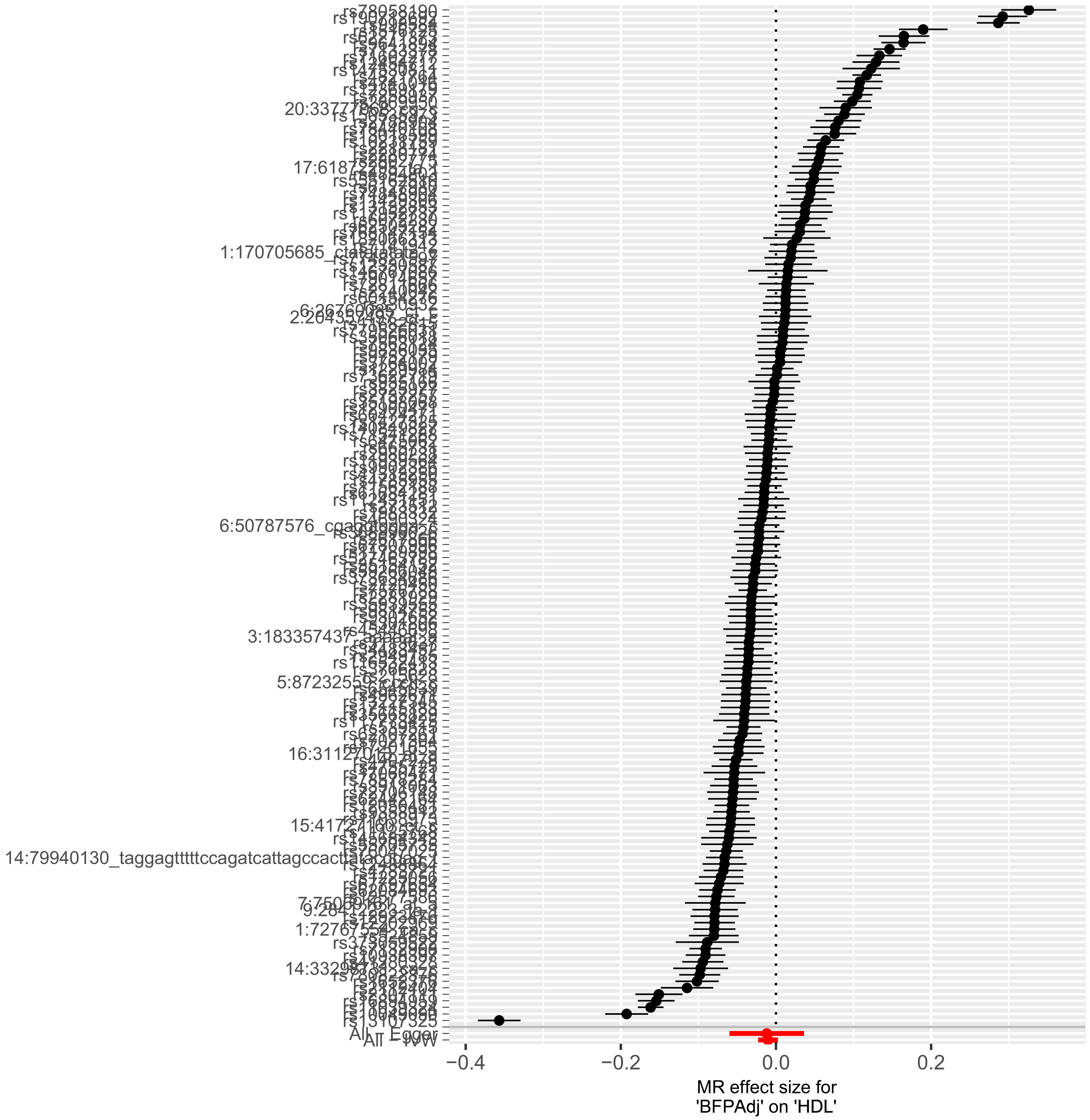
rs78058190
rs13107325
All - Egger
All - IVW
−0.4
−0.2
0.0
0.2
MR effect size for
'BFPAdj' on 'HDL'

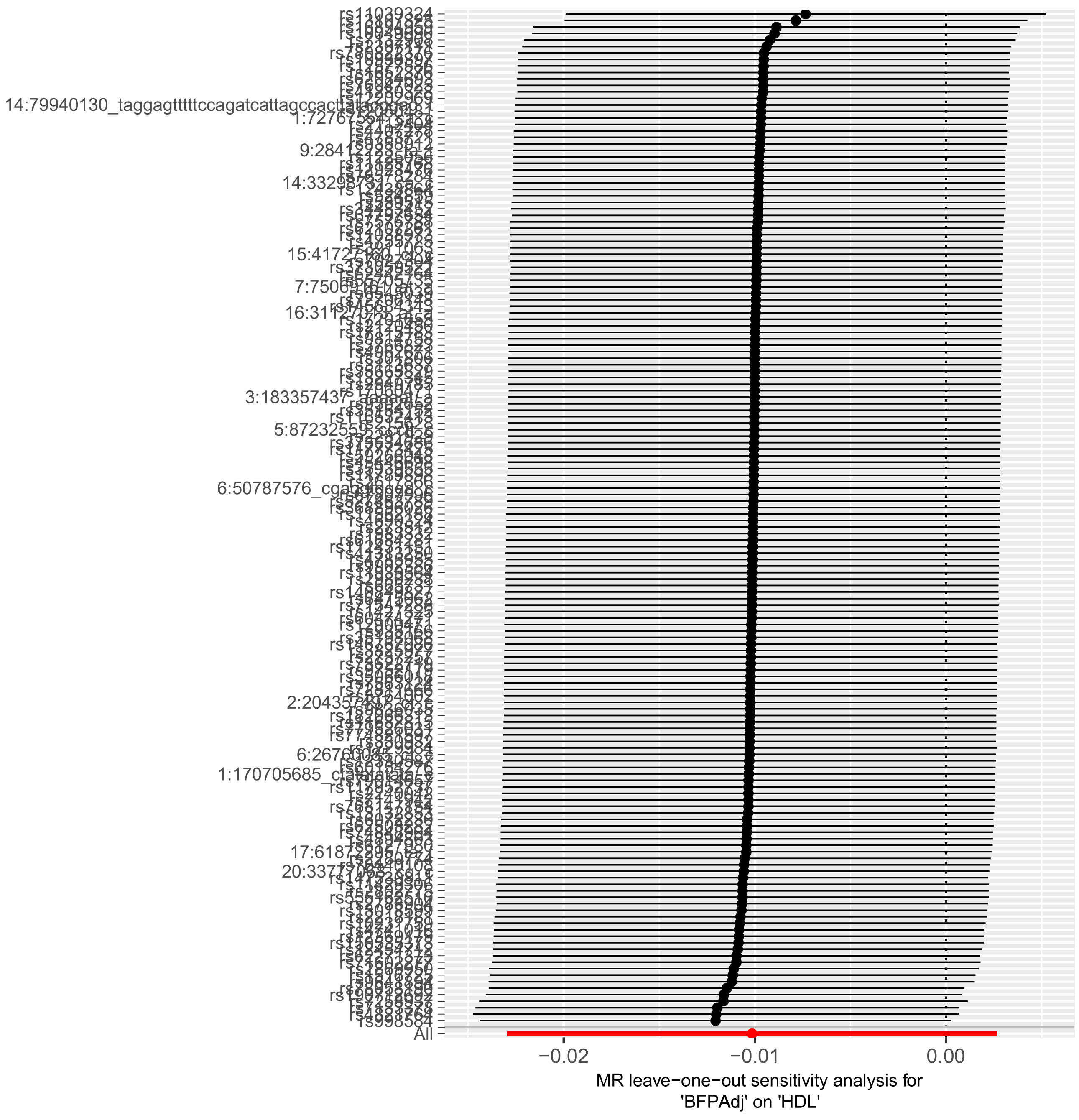
rs11039324
rs998564
All
−0.02
−0.01
0.00
MR leave−one−out sensitivity analysis for
'BFPAdj' on 'HDL'

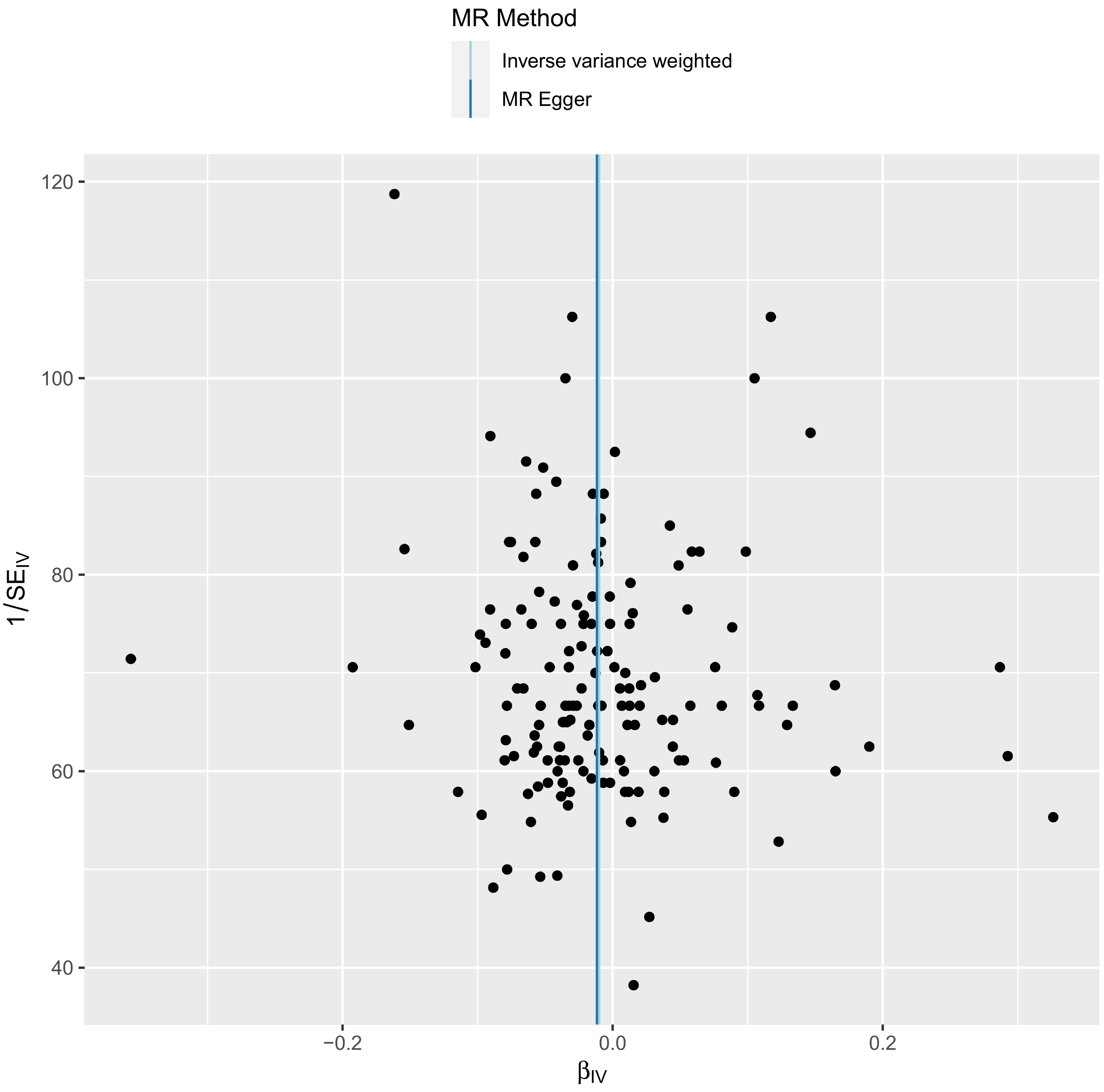

MR Method
Inverse variance weighted
MR Egger
120
100
80
60
40
1/SE$_{IV}$
−0.2
0.0
0.2
β$_{IV}$

4- TG

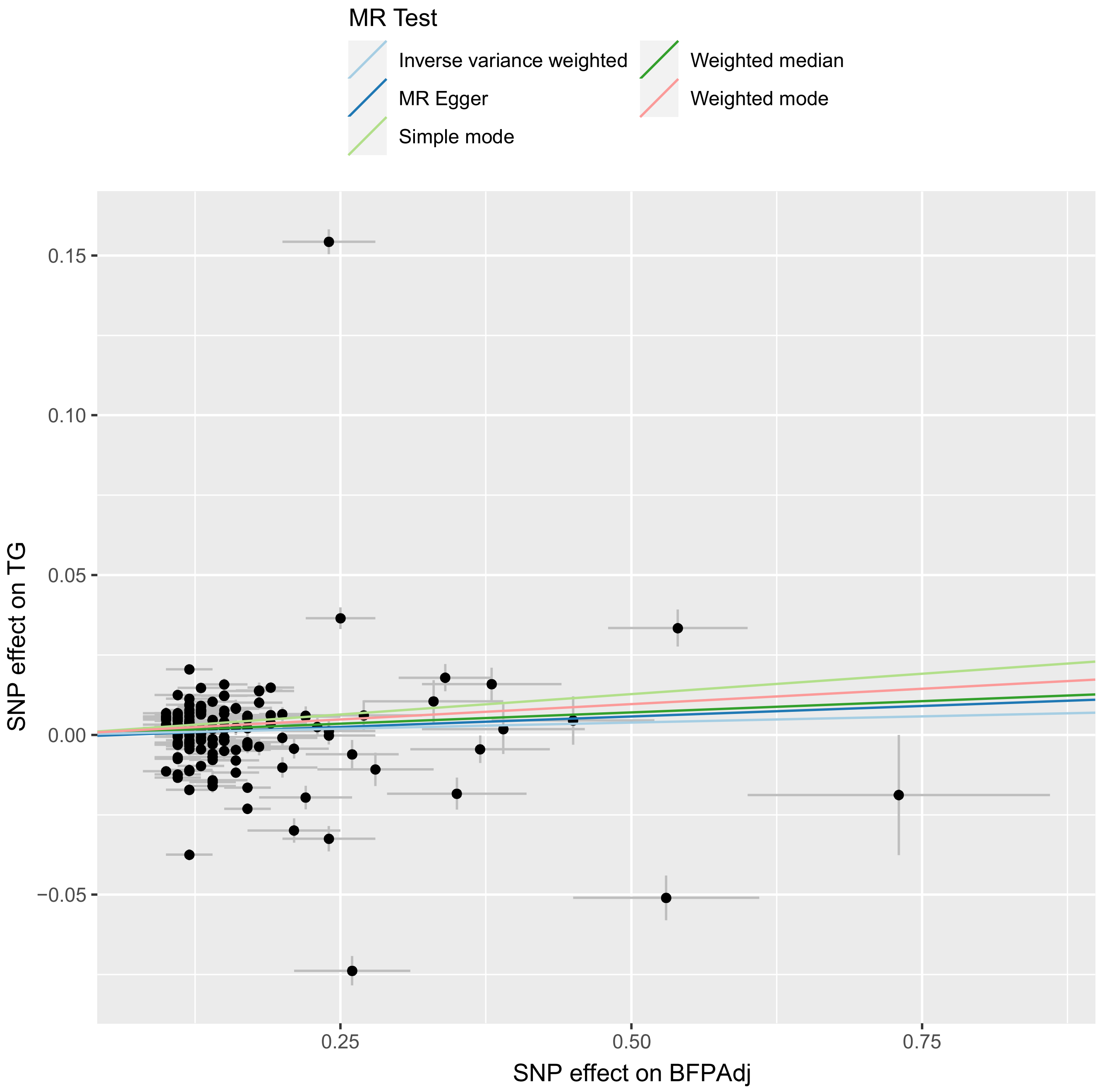
MR Test
Inverse variance weighted
MR Egger
Simple mode
Weighted median
Weighted mode
0.15
0.10
0.05
0.00
−0.05
SNP effect on TG
0.25
0.50
0.75
SNP effect on BFPAdj

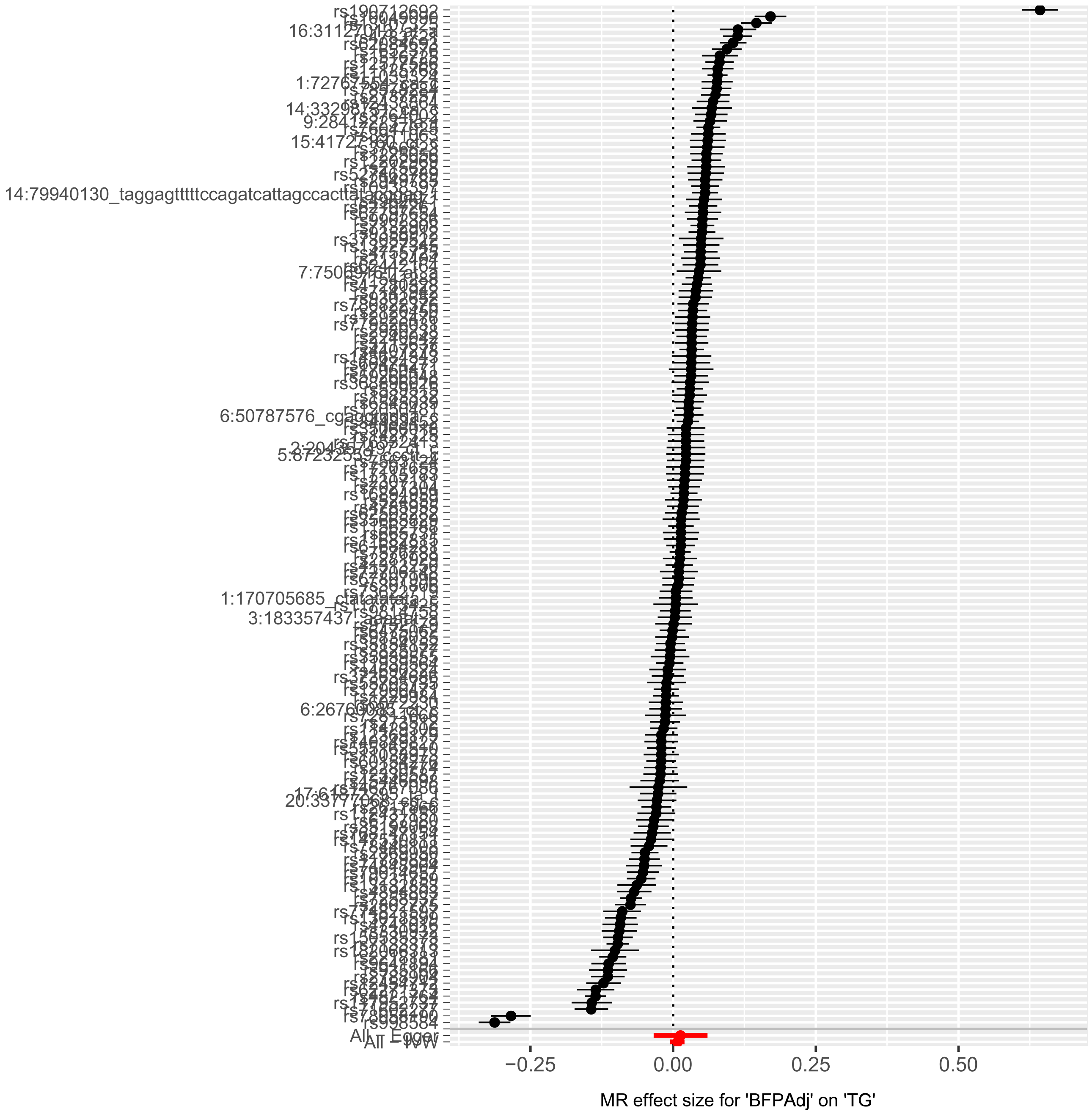

rs190712692
rs998584
All - Egger
All - IVW
−0.25
0.00
0.25
0.50
MR effect size for 'BFPAdj' on 'TG'

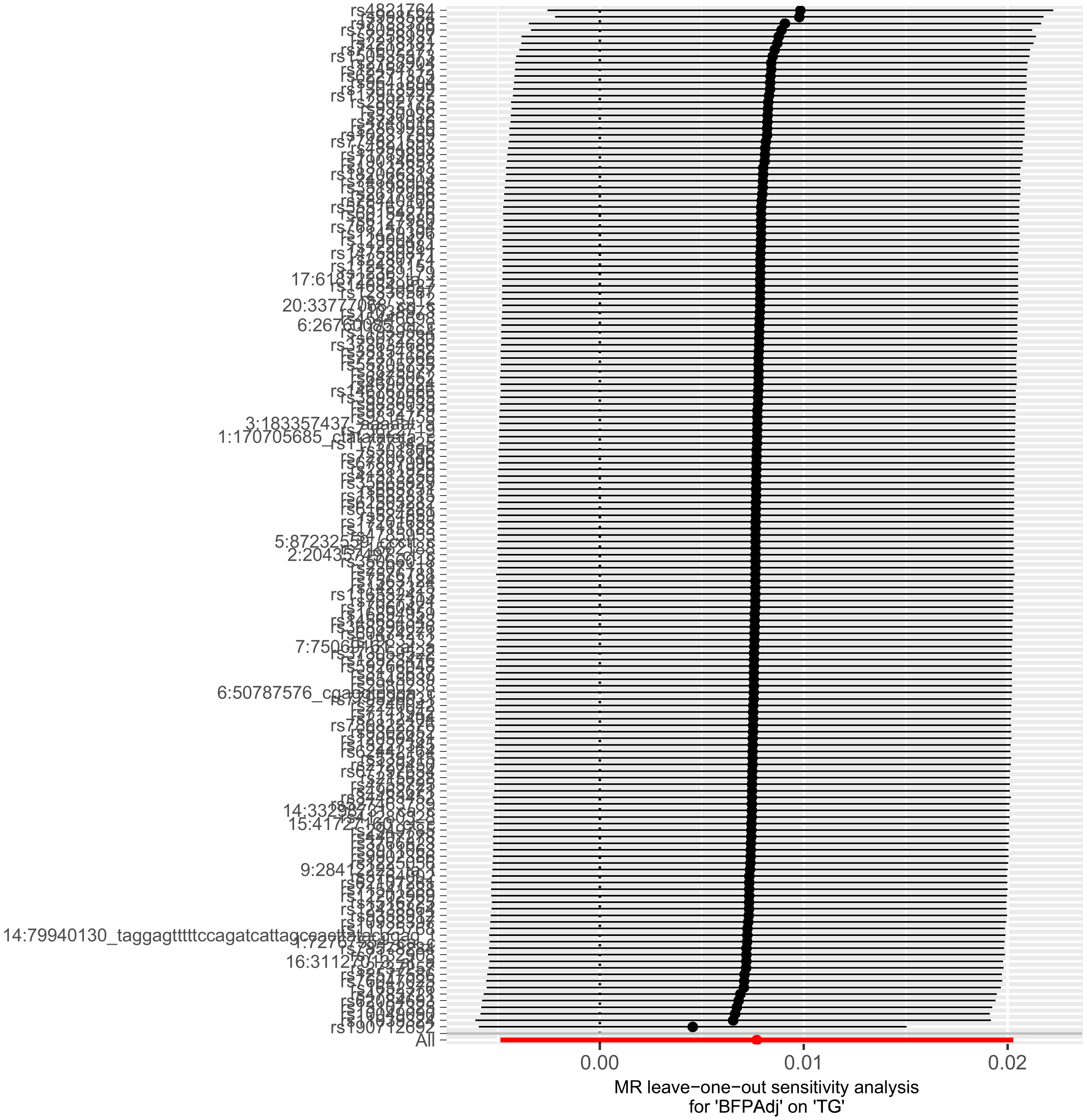
rs4821764
All
0.00
0.01
0.02
MR leave−one−out sensitivity analysis
for 'BFPAdj' on 'TG'

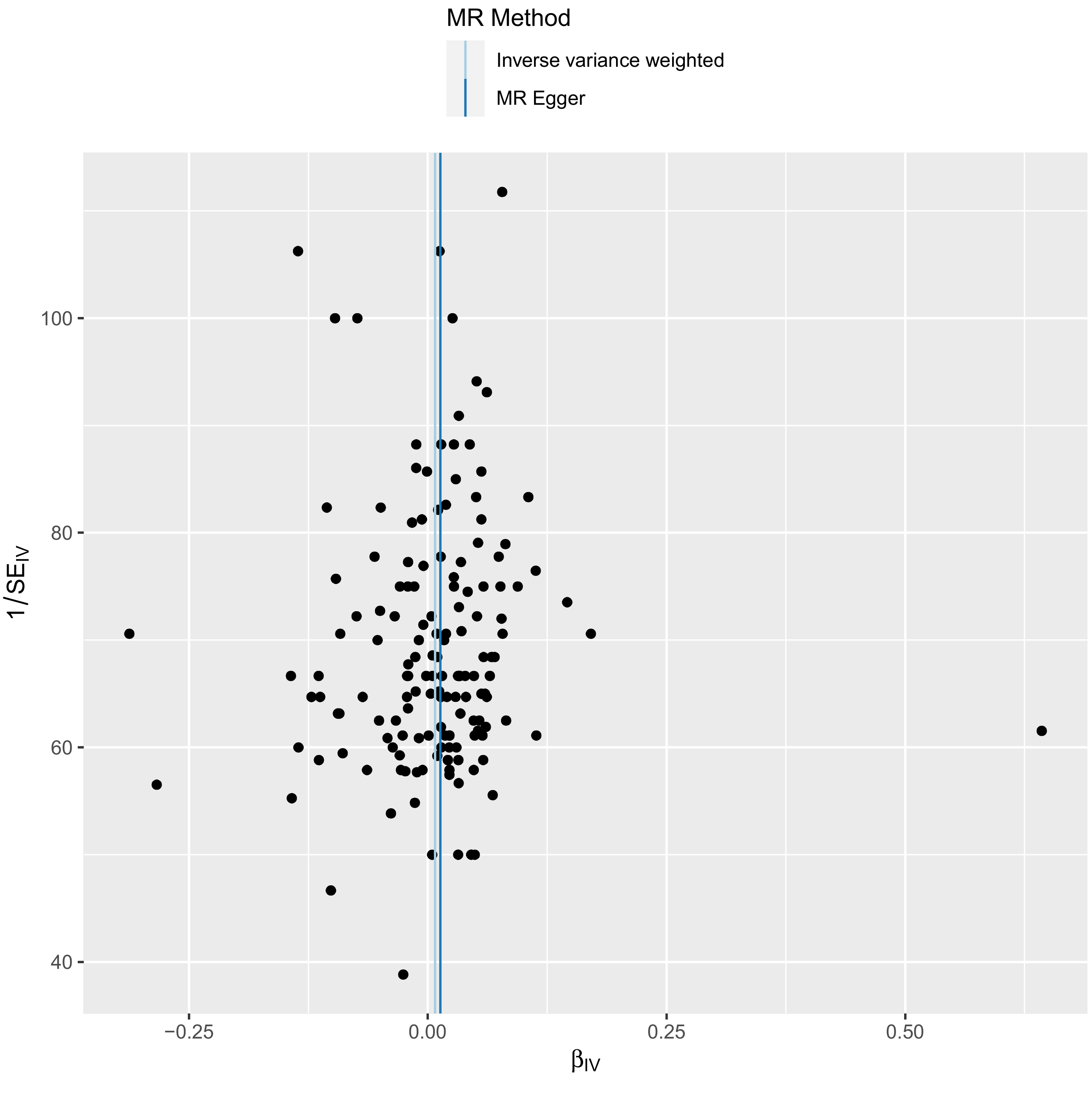

MR Method
Inverse variance weighted
MR Egger
100
80
60
40
1/SE$_{IV}$
−0.25
0.00
0.25
0.50
β$_{IV}$

MR Analysis in Females

1- T2D

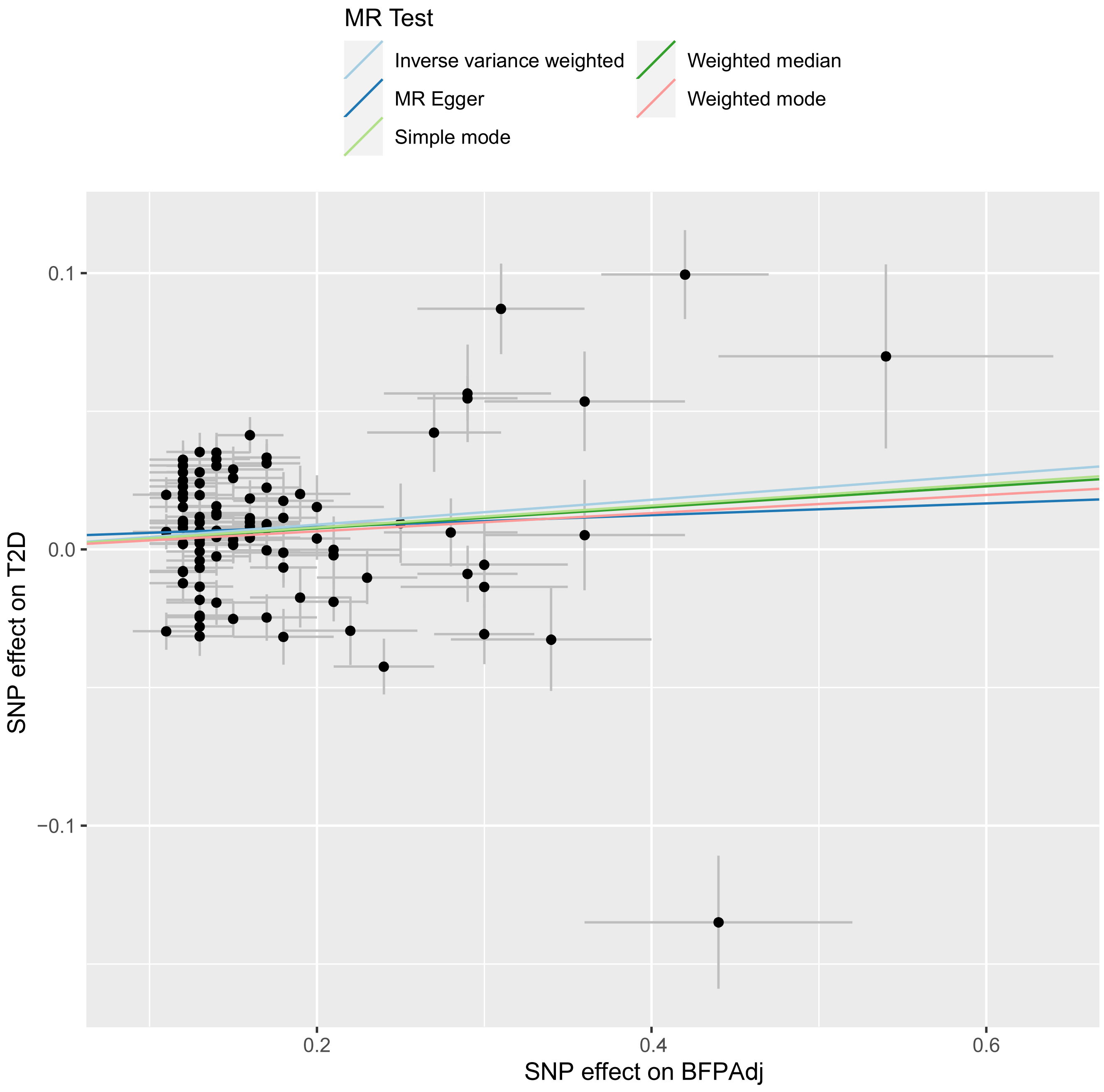
MR Test
Inverse variance weighted
MR Egger
Simple mode
Weighted median
Weighted mode
0.1
0.0
−0.1
SNP effect on T2D
0.2
0.4
0.6
SNP effect on BFPAdj

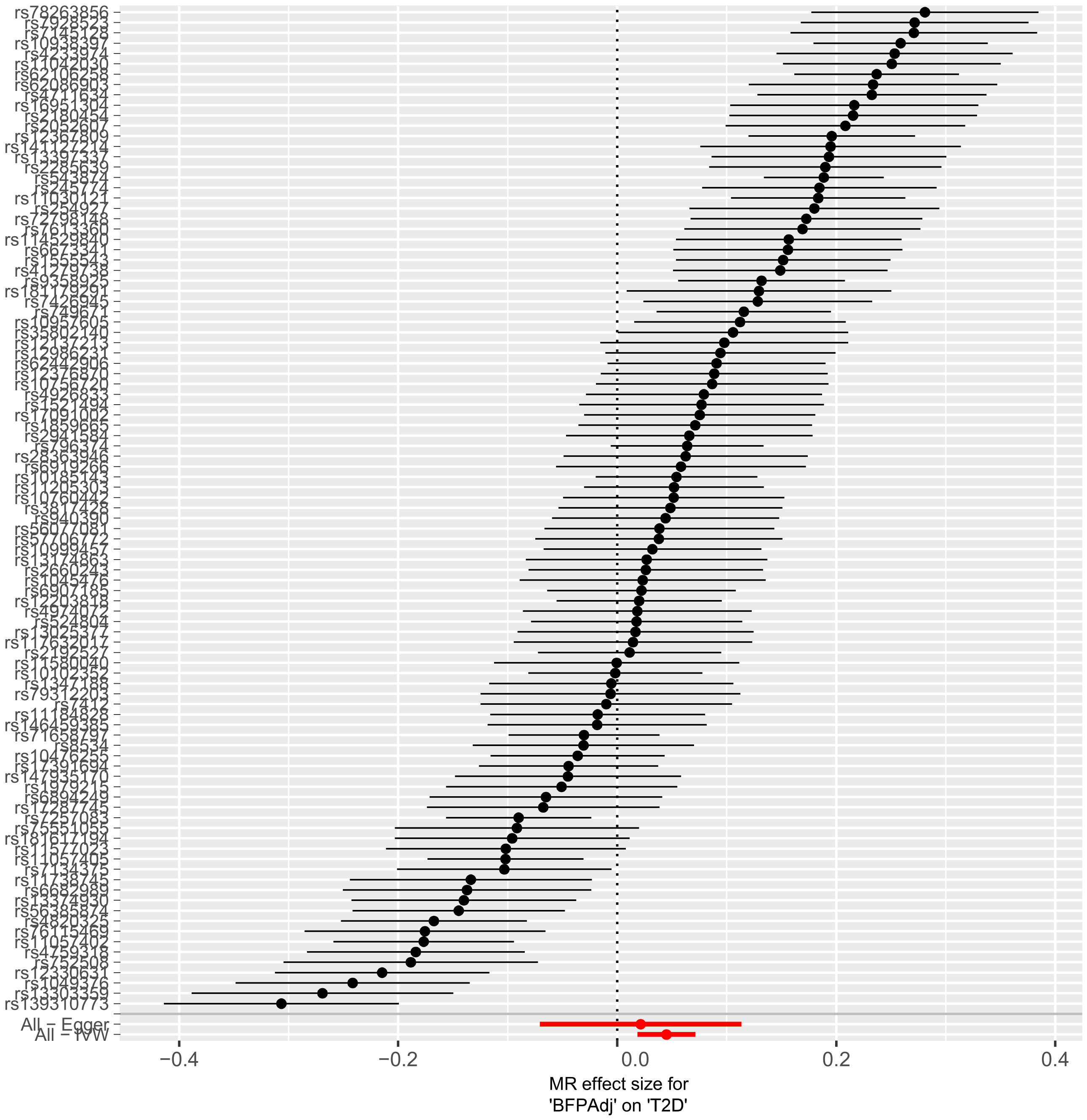
rs78263856
rs139310773
All − Egger
All − IVW
−0.4
−0.2
0.0
0.2
0.4
MR effect size for
'BFPAdj' on 'T2D'

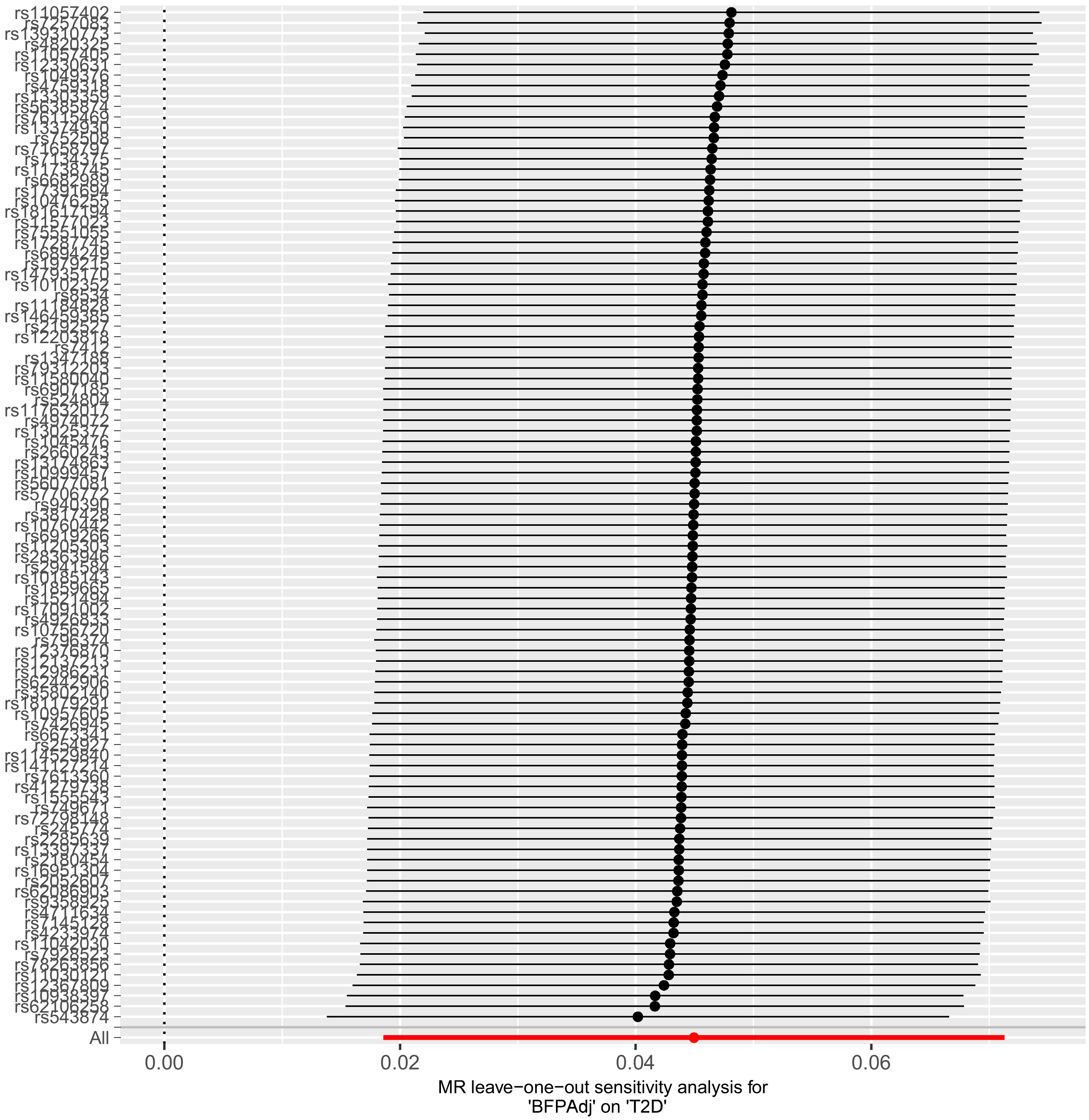
All
0.00
0.02
0.04
0.06
MR leave−one−out sensitivity analysis for
'BFPAdj' on 'T2D'

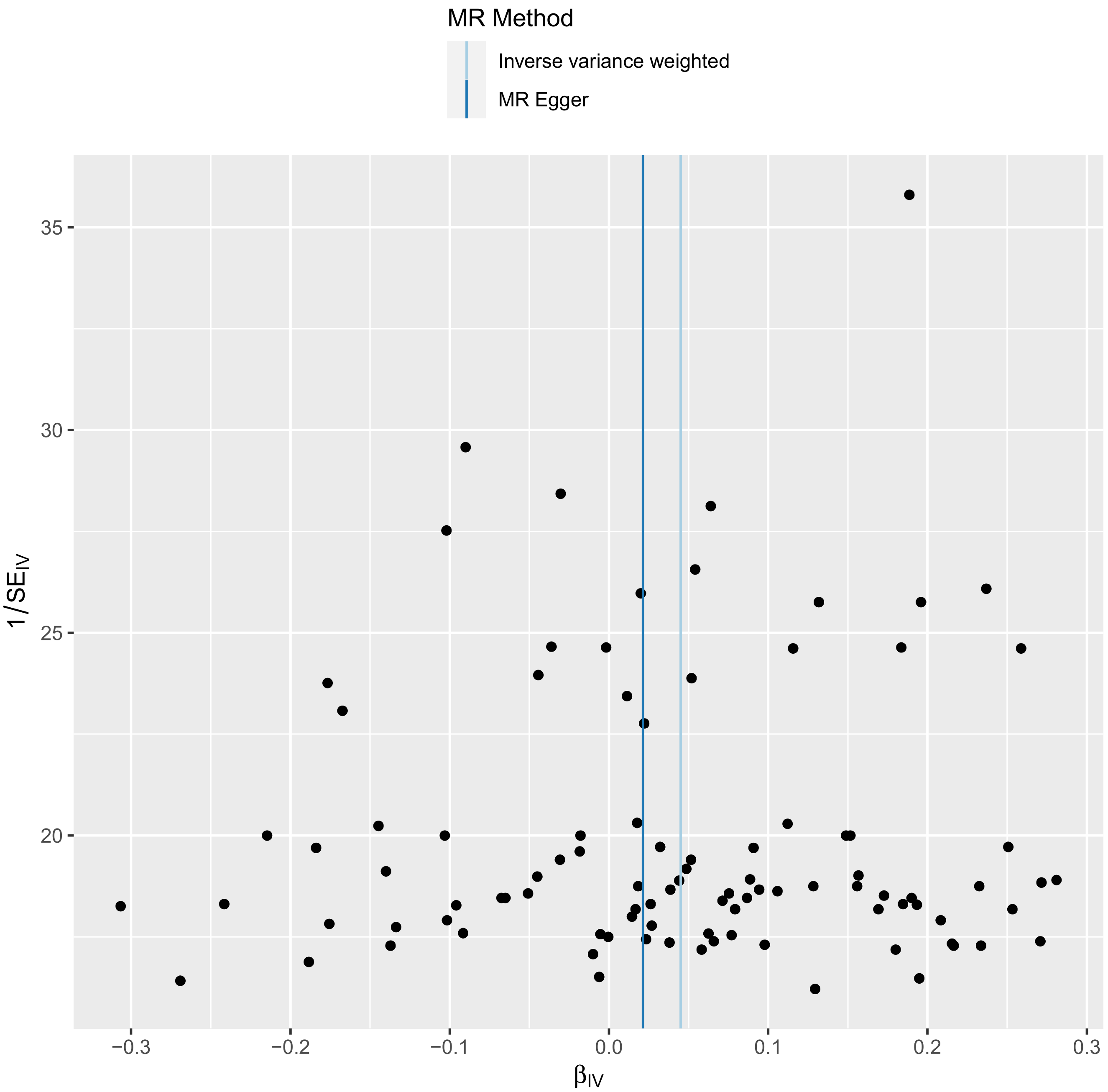

MR Method
Inverse variance weighted
MR Egger
35
30
25
20
$1/SE_{IV}$
−0.3
−0.2
−0.1
0.0
0.1
0.2
0.3
β_{IV}

2- CAD

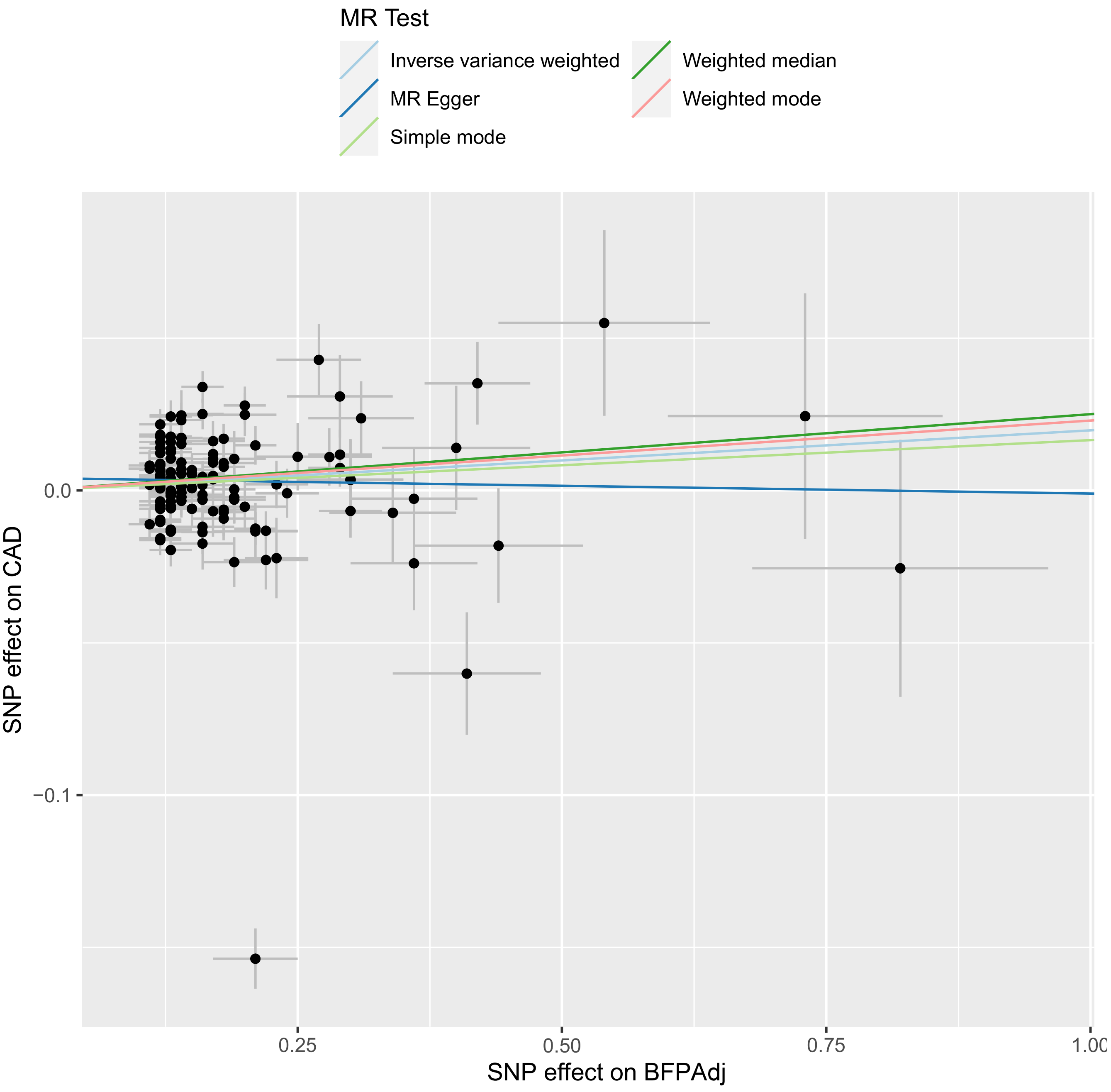
MR Test
Inverse variance weighted
MR Egger
Simple mode
Weighted median
Weighted mode
0.0
−0.1
SNP effect on CAD
0.25
0.50
0.75
1.00
SNP effect on BFPAdj

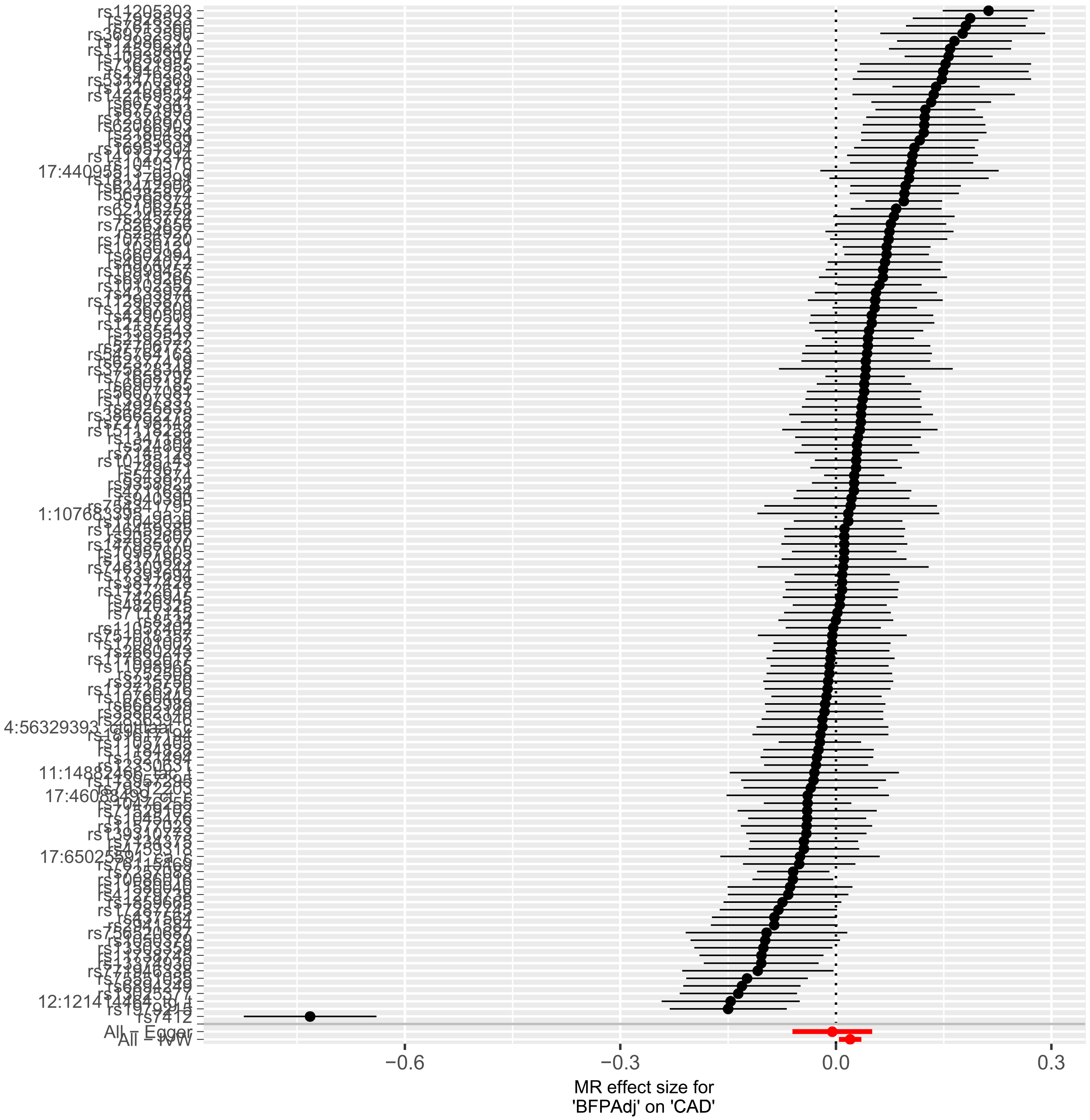
rs11205303
rs7412
All - Egger
All - IVW
−0.6
−0.3
0.0
0.3
MR effect size for
'BFPAdj' on 'CAD'

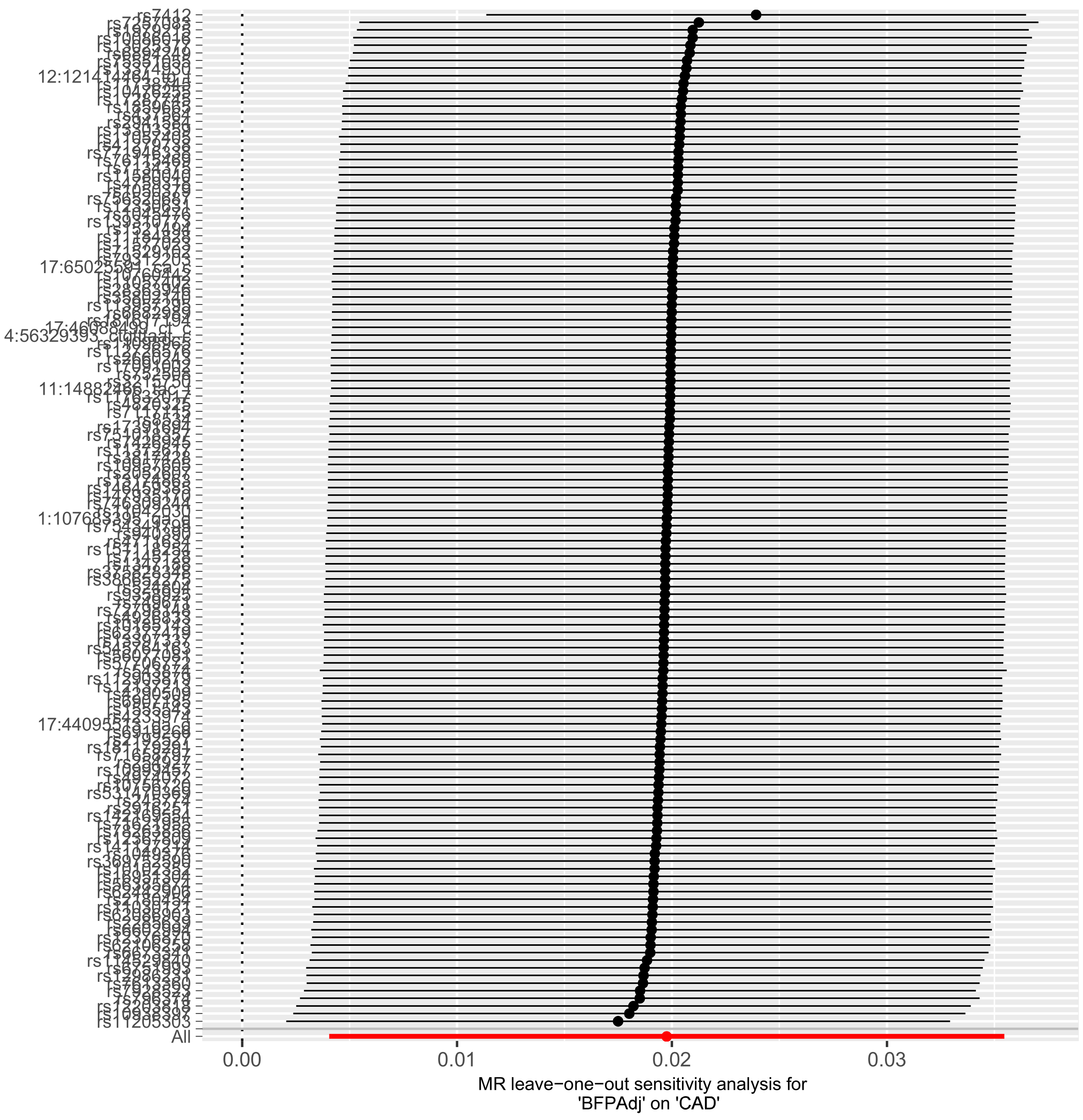
rs7412
rs11205303
All
0.00
0.01
0.02
0.03
MR leave−one−out sensitivity analysis for
'BFPAdj' on 'CAD'

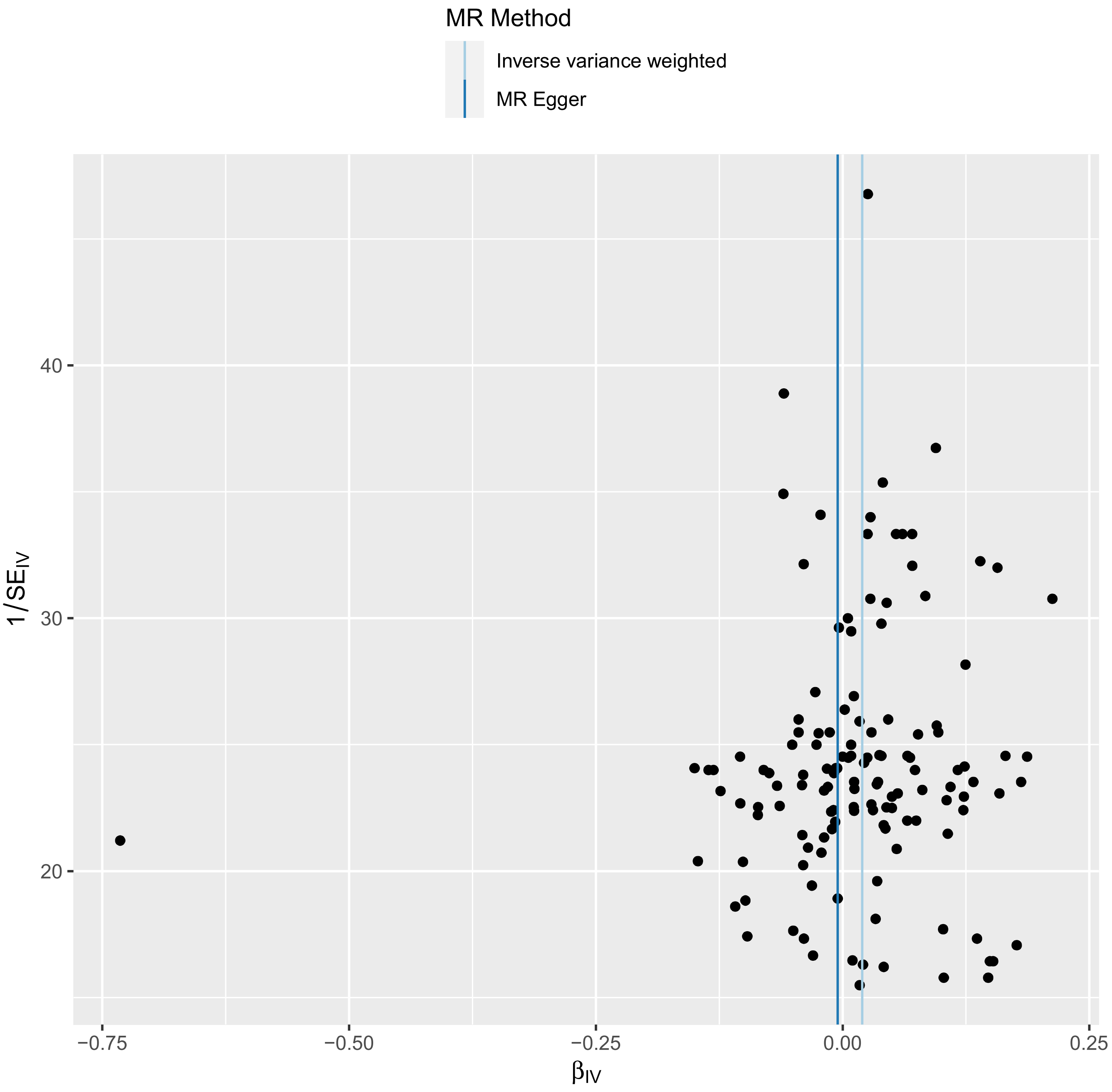
MR Method
Inverse variance weighted
MR Egger
40
30
20
1/SE$_{IV}$
−0.75
−0.50
−0.25
0.00
0.25
β$_{IV}$

3- HDL

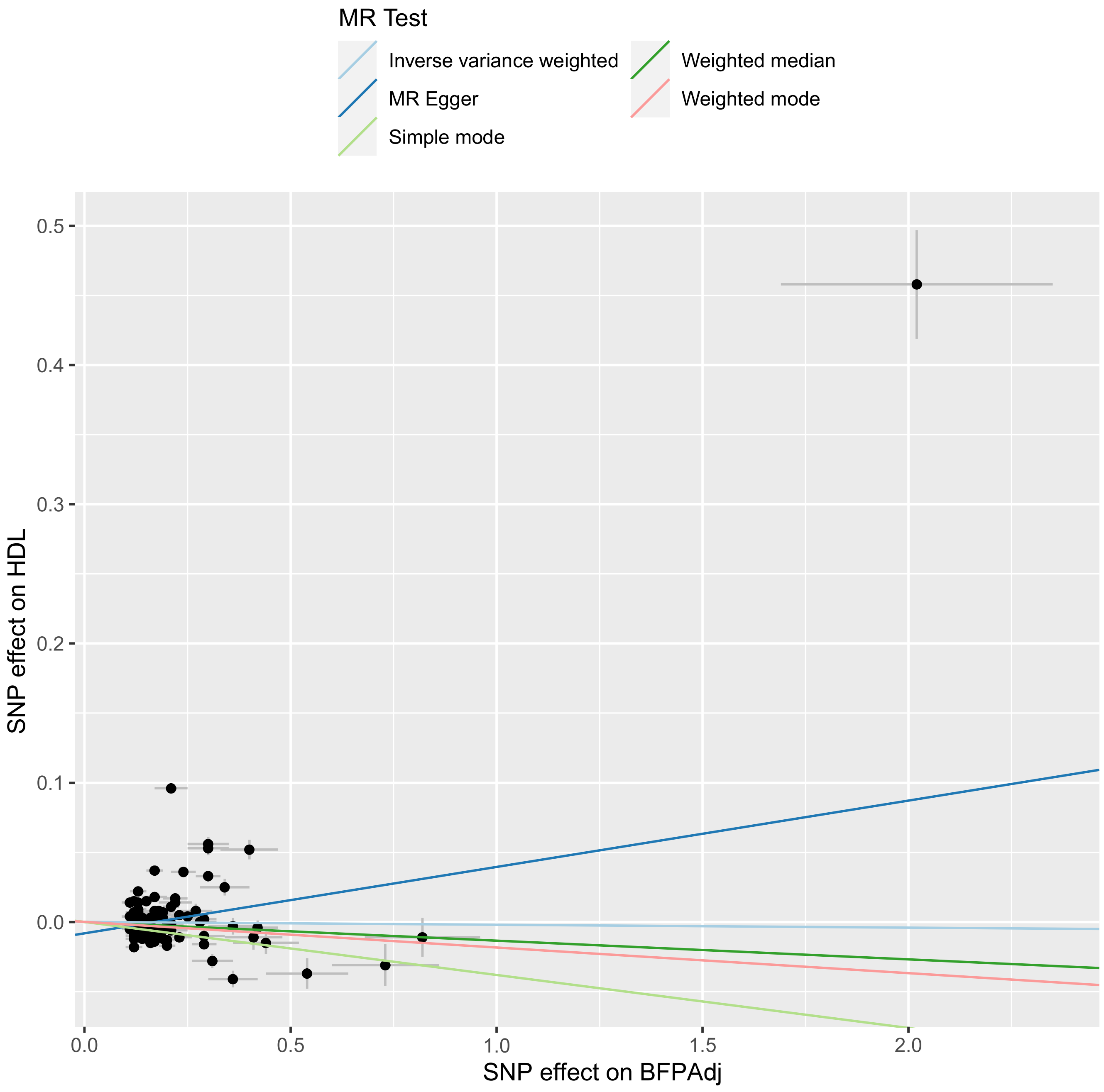
MR Test
Inverse variance weighted
MR Egger
Simple mode
Weighted median
Weighted mode
0.5
0.4
0.3
0.2
0.1
0.0
SNP effect on HDL
0.0
0.5
1.0
1.5
2.0
SNP effect on BFPAdj

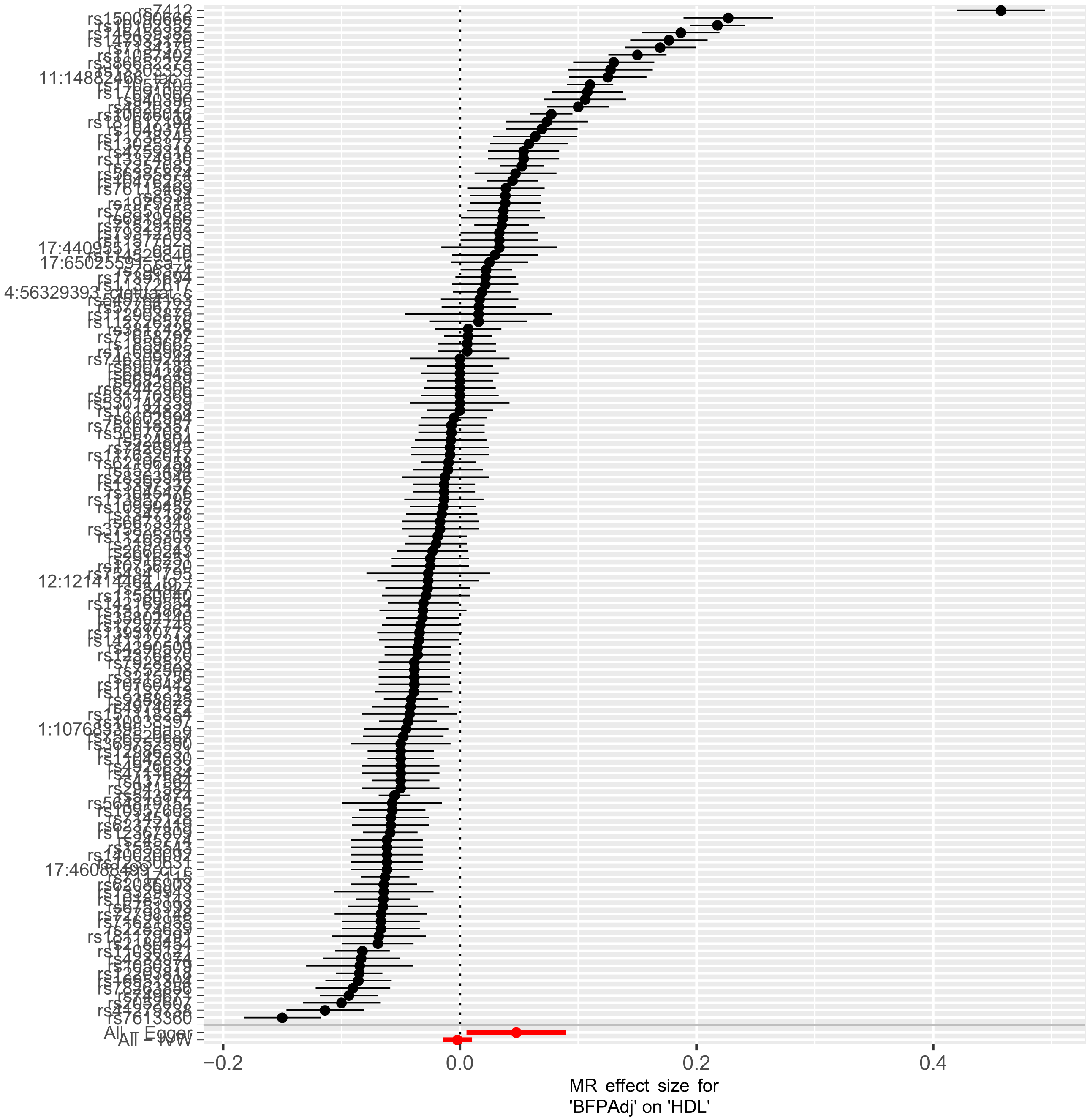
rs7412
All - Egger
All - IVW
−0.2
0.0
0.2
0.4
MR effect size for
'BFPAdj' on 'HDL'

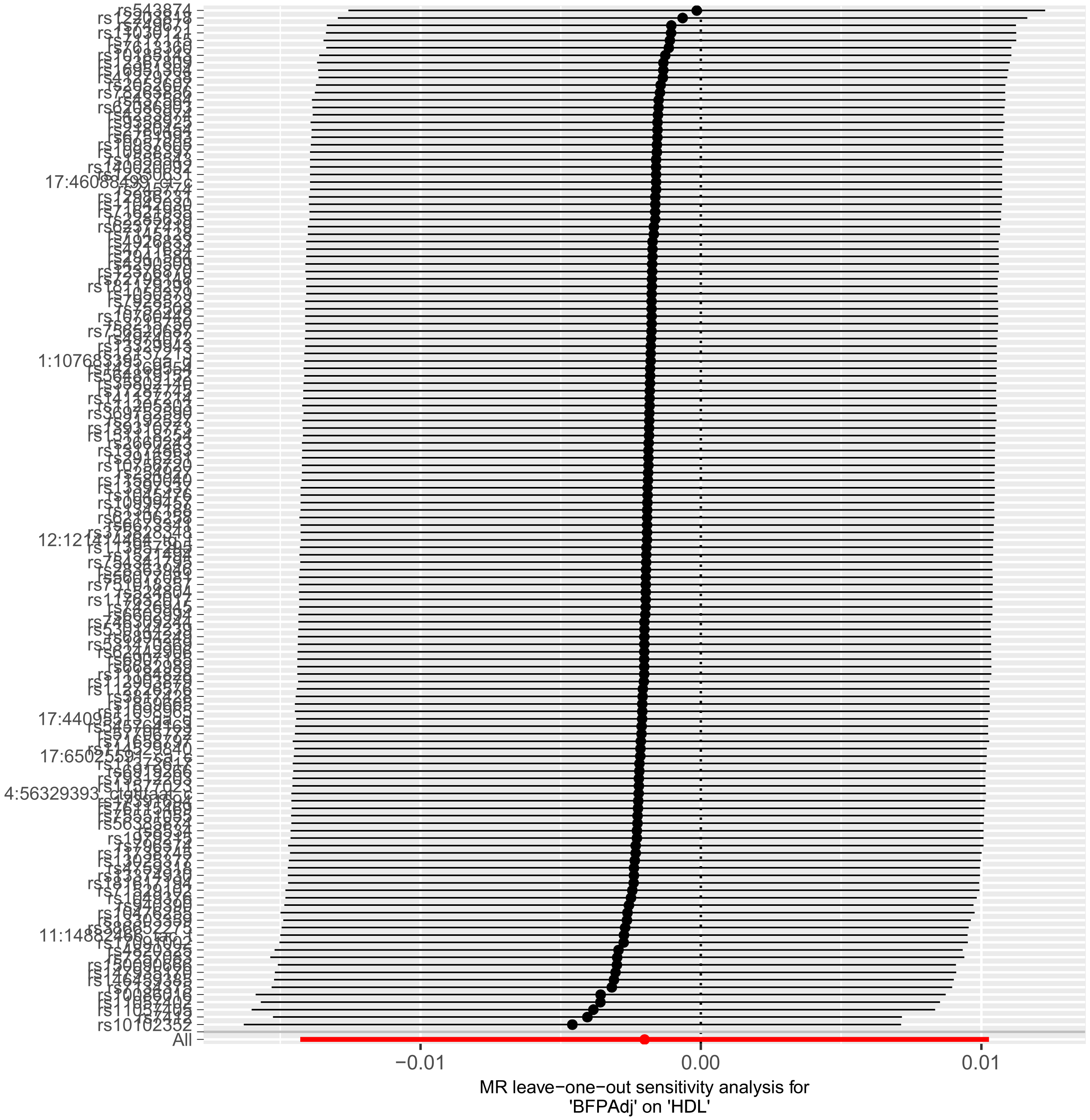
rs543874
rs10102352
All
−0.01
0.00
0.01
MR leave−one−out sensitivity analysis for
'BFPAdj' on 'HDL'

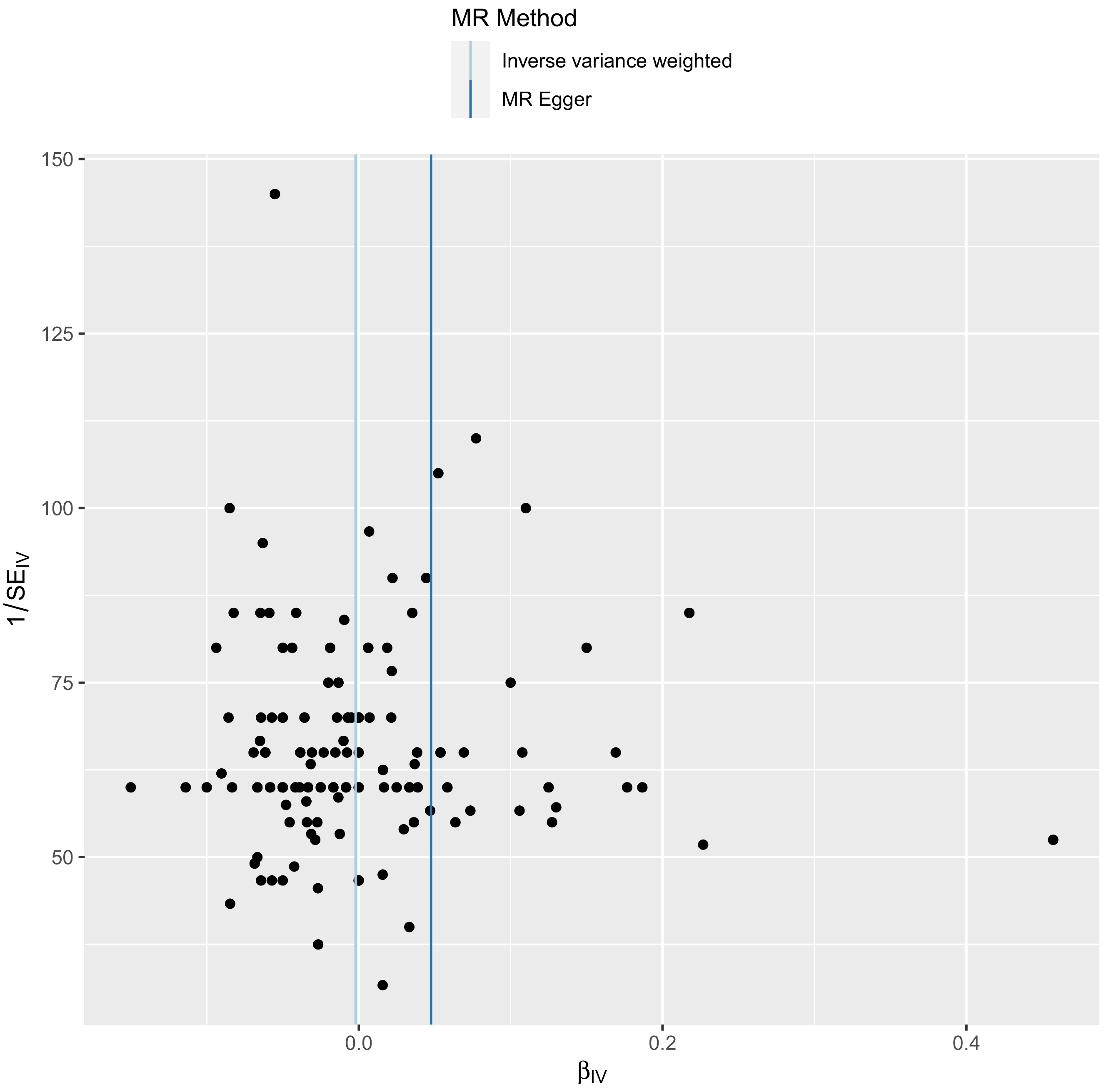
MR Method
Inverse variance weighted
MR Egger
150
125
100
75
50
1/SE_{IV}
0.0
0.2
0.4
β_{IV}

4- TG

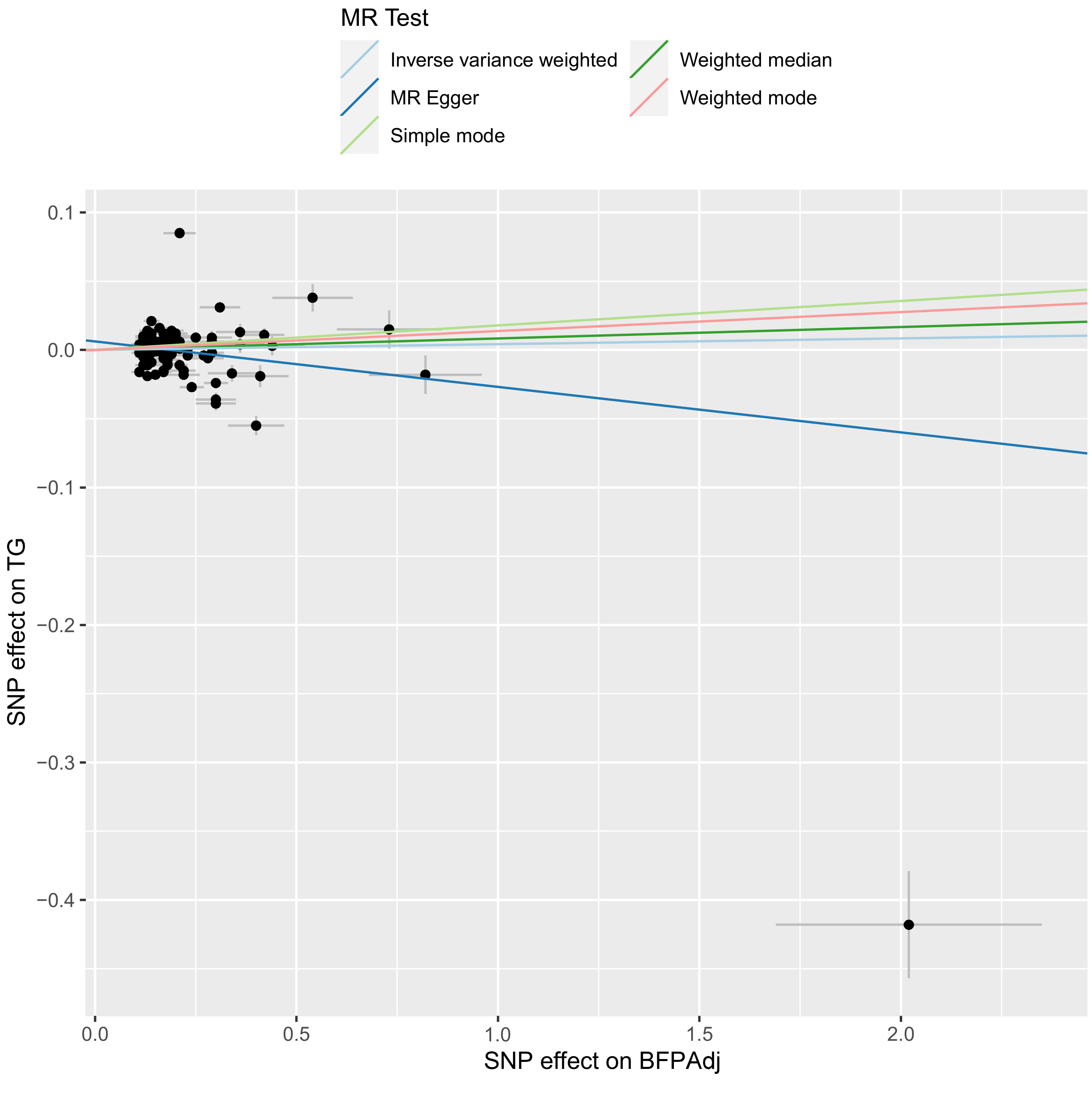
MR Test
Inverse variance weighted
MR Egger
Simple mode
Weighted median
Weighted mode
0.1
0.0
−0.1
−0.2
−0.3
−0.4
SNP effect on TG
0.0
0.5
1.0
1.5
2.0
SNP effect on BFPAdj

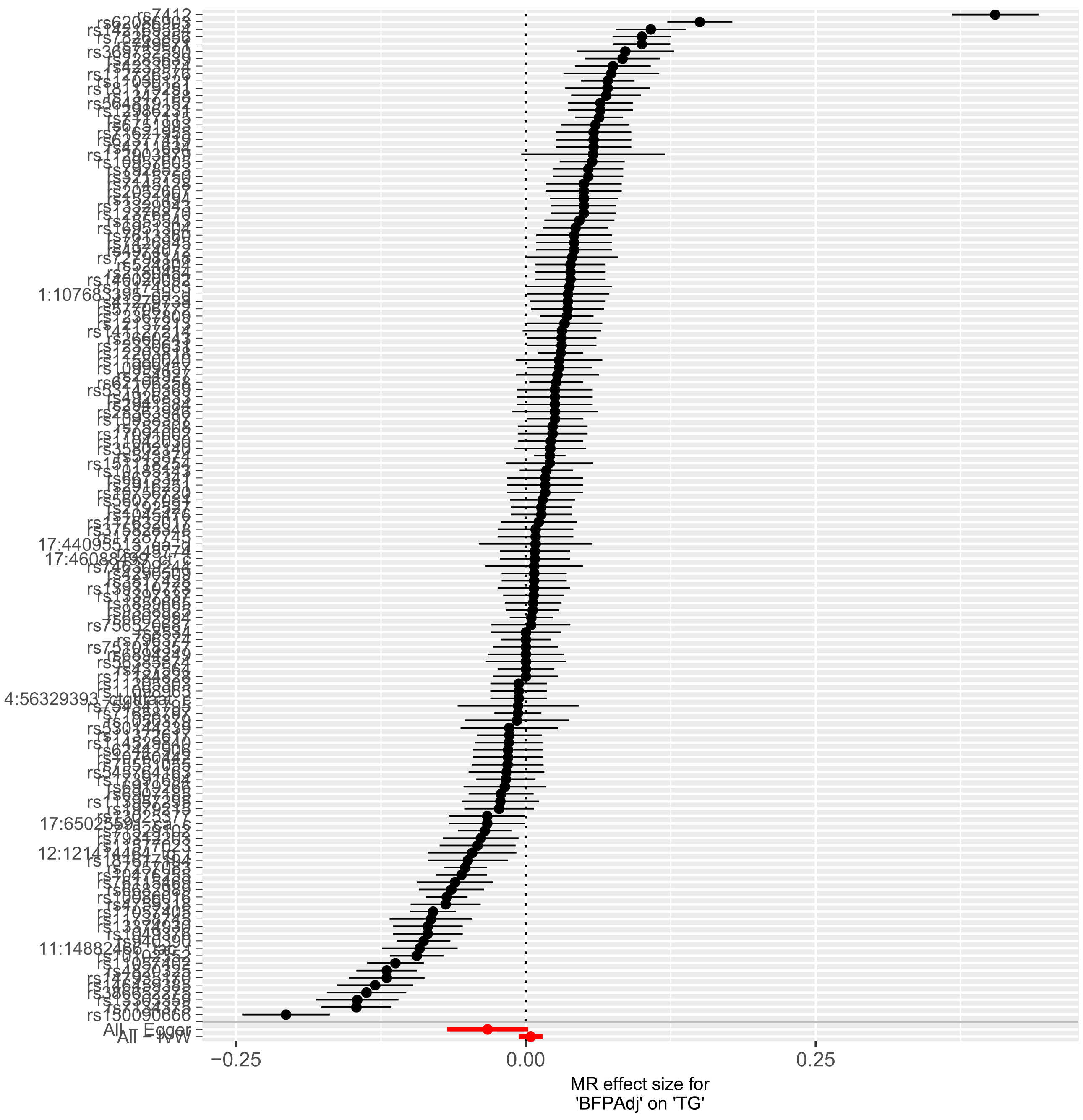

rs7412
rs150090666
All - Egger
All - IVW
−0.25
0.00
0.25
MR effect size for
'BFPAdj' on 'TG'

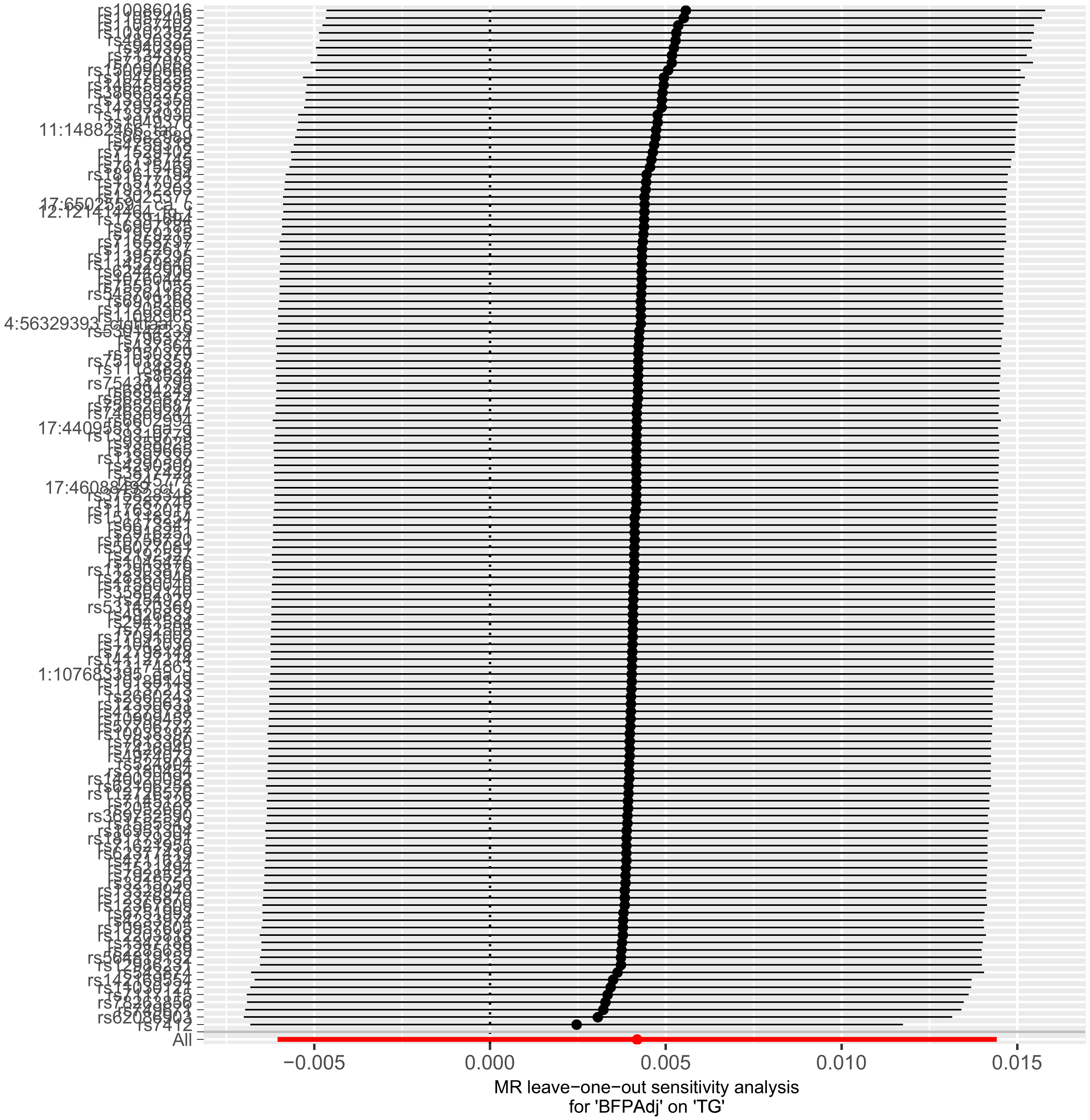
rs10086016
rs7412
All
−0.005
0.000
0.005
0.010
0.015
MR leave−one−out sensitivity analysis
for 'BFPAdj' on 'TG'

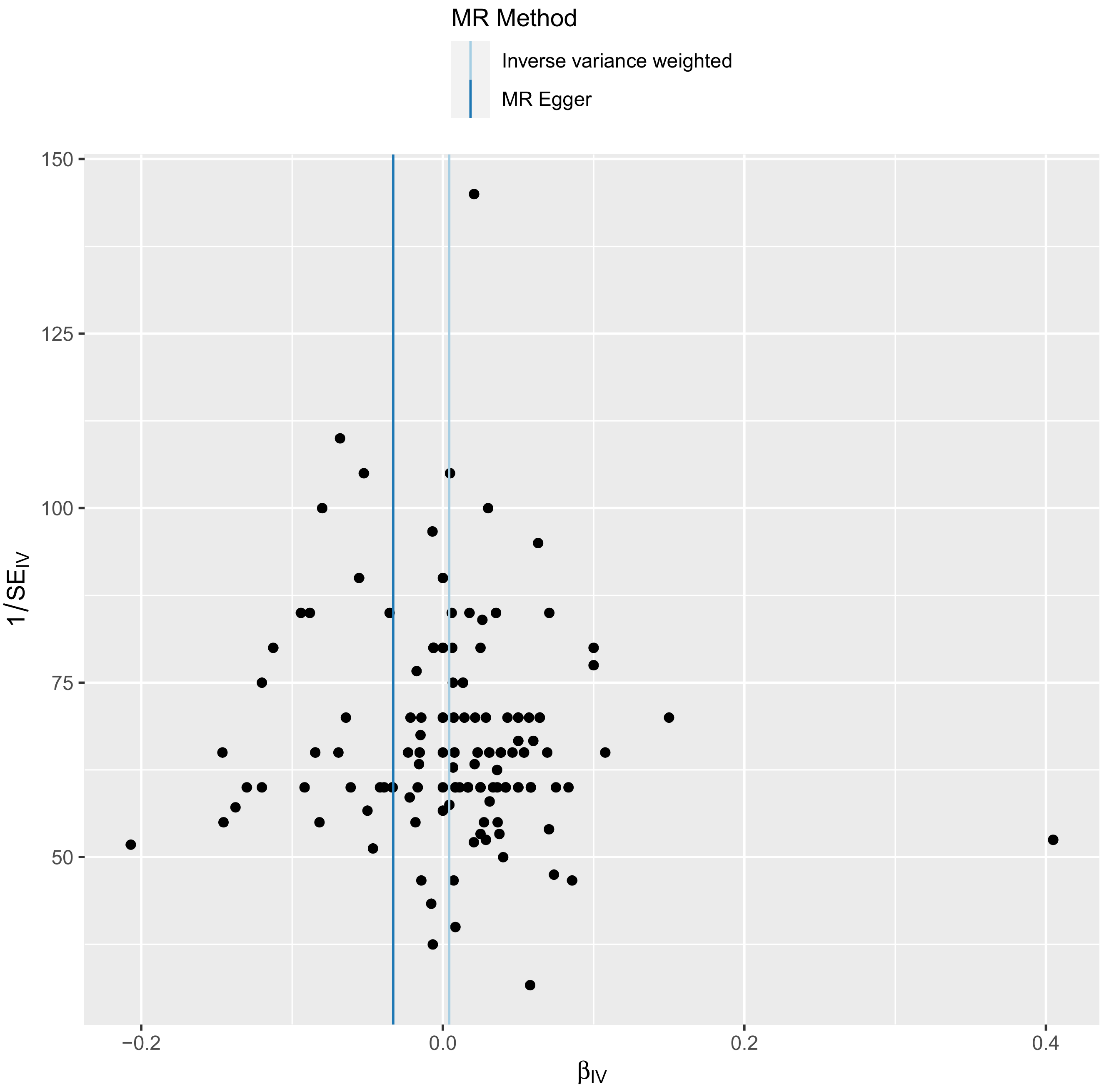

MR Method
Inverse variance weighted
MR Egger
150
125
100
75
50
1/SE_IV
−0.2
0.0
0.2
0.4
β_IV
